# Healthcare Access and Quality Index based on mortality from causes amenable to personal health care in 195 countries and territories, 1990–2015: a novel analysis from the Global Burden of Disease Study 2015

**DOI:** 10.1016/S0140-6736(17)30818-8

**Published:** 2017-07-15

**Authors:** GBD 2015 Healthcare Access and Quality Collaborators, Ryan M Barber, Nancy Fullman, Reed J D Sorensen, Thomas Bollyky, Martin McKee, Ellen Nolte, Amanuel Alemu Abajobir, Kalkidan Hassen Abate, Cristiana Abbafati, Kaja M Abbas, Foad Abd-Allah, Abdishakur M Abdulle, Ahmed Abdulahi Abdurahman, Semaw Ferede Abera, Biju Abraham, Girmatsion Fisseha Abreha, Kelemework Adane, Ademola Lukman Adelekan, Ifedayo Morayo O Adetifa, Ashkan Afshin, Arnav Agarwal, Sanjay Kumar Agarwal, Sunilkumar Agarwal, Anurag Agrawal, Aliasghar Ahmad Kiadaliri, Alireza Ahmadi, Kedir Yimam Ahmed, Muktar Beshir Ahmed, Rufus Olusola Akinyemi, Tomi F Akinyemiju, Nadia Akseer, Ziyad Al-Aly, Khurshid Alam, Noore Alam, Sayed Saidul Alam, Zewdie Aderaw Alemu, Kefyalew Addis Alene, Lily Alexander, Raghib Ali, Syed Danish Ali, Reza Alizadeh-Navaei, Ala'a Alkerwi, François Alla, Peter Allebeck, Christine Allen, Rajaa Al-Raddadi, Ubai Alsharif, Khalid A Altirkawi, Elena Alvarez Martin, Nelson Alvis-Guzman, Azmeraw T Amare, Erfan Amini, Walid Ammar, Joshu Amo-Adjei, Yaw Ampem Amoako, Benjamin O Anderson, Sofia Androudi, Hossein Ansari, Mustafa Geleto Ansha, Carl Abelardo T Antonio, Johan Ärnlöv, Al Artaman, Hamid Asayesh, Reza Assadi, Ayalew Astatkie, Tesfay Mehari Atey, Suleman Atique, Niguse Tadele Atnafu, Sachin R Atre, Leticia Avila-Burgos, Euripide Frinel G Arthur Avokpaho, Beatriz Paulina Ayala Quintanilla, Ashish Awasthi, Nebiyu Negussu Ayele, Peter Azzopardi, Huda Omer Ba Saleem, Till Bärnighausen, Umar Bacha, Alaa Badawi, Amitava Banerjee, Aleksandra Barac, Miguel A Barboza, Suzanne L Barker-Collo, Lope H Barrero, Sanjay Basu, Bernhard T Baune, Kaleab Baye, Yibeltal Tebekaw Bayou, Shahrzad Bazargan-Hejazi, Neeraj Bedi, Ettore Beghi, Yannick Béjot, Aminu K Bello, Derrick A Bennett, Isabela M Bensenor, Adugnaw Berhane, Eduardo Bernabé, Oscar Alberto Bernal, Addisu Shunu Beyene, Tariku Jibat Beyene, Zulfiqar A Bhutta, Sibhatu Biadgilign, Boris Bikbov, Sait Mentes Birlik, Charles Birungi, Stan Biryukov, Donal Bisanzio, Habtamu Mellie Bizuayehu, Dipan Bose, Michael Brainin, Michael Brauer, Alexandra Brazinova, Nicholas J K Breitborde, Hermann Brenner, Zahid A Butt, Rosario Cárdenas, Lucero Cahuana-Hurtado, Ismael Ricardo Campos-Nonato, Josip Car, Juan Jesus Carrero, Daniel Casey, Valeria Caso, Carlos A Castañeda-Orjuela, Jacqueline Castillo Rivas, Ferrán Catalá-López, Pedro Cecilio, Kelly Cercy, Fiona J Charlson, Alan Z Chen, Adrienne Chew, Mirriam Chibalabala, Chioma Ezinne Chibueze, Vesper Hichilombwe Chisumpa, Abdulaal A Chitheer, Rajiv Chowdhury, Hanne Christensen, Devasahayam Jesudas Christopher, Liliana G Ciobanu, Massimo Cirillo, Megan S Coggeshall, Leslie Trumbull Cooper, Monica Cortinovis, John A Crump, Koustuv Dalal, Hadi Danawi, Lalit Dandona, Rakhi Dandona, Paul I Dargan, Jose das Neves, Gail Davey, Dragos V Davitoiu, Kairat Davletov, Diego De Leo, Liana C Del Gobbo, Borja del Pozo-Cruz, Robert P Dellavalle, Kebede Deribe, Amare Deribew, Don C Des Jarlais, Subhojit Dey, Samath D Dharmaratne, Daniel Dicker, Eric L Ding, Klara Dokova, E Ray Dorsey, Kerrie E Doyle, Manisha Dubey, Rebecca Ehrenkranz, Christian Lycke Ellingsen, Iqbal Elyazar, Ahmadali Enayati, Sergey Petrovich Ermakov, Babak Eshrati, Alireza Esteghamati, Kara Estep, Thomas Fürst, Imad D A Faghmous, Fanuel Belayneh Bekele Fanuel, Emerito Jose Aquino Faraon, Talha A Farid, Carla Sofia e Sa Farinha, Andre Faro, Maryam S Farvid, Farshad Farzadfar, Valery L Feigin, Andrea B Feigl, Seyed-Mohammad Fereshtehnejad, Jefferson G Fernandes, João C Fernandes, Tesfaye Regassa Feyissa, Florian Fischer, Christina Fitzmaurice, Thomas D Fleming, Nataliya Foigt, Kyle J Foreman, Mohammad H Forouzanfar, Richard C Franklin, Joseph Frostad, Tsegaye Tewelde G/hiwot, Emmanuela Gakidou, Ketevan Gambashidze, Amiran Gamkrelidze, Wayne Gao, Alberto L Garcia-Basteiro, Teshome Gebre, Amanuel Tesfay Gebremedhin, Mengistu Welday Gebremichael, Alemseged Aregay Gebru, Amha Admasie Gelaye, Johanna M Geleijnse, Ricard Genova-Maleras, Katherine B Gibney, Ababi Zergaw Giref, Melkamu Dedefo Gishu, Giorgia Giussani, William W Godwin, Audra Gold, Ellen M Goldberg, Philimon N Gona, Amador Goodridge, Sameer Vali Gopalani, Atsushi Goto, Nicholas Graetz, Felix Greaves, Max Griswold, Peter Imre Guban, Harish Chander Gugnani, Prakash C Gupta, Rahul Gupta, Rajeev Gupta, Tanush Gupta, Vipin Gupta, Tesfa Dejenie Habtewold, Nima Hafezi-Nejad, Demewoz Haile, Alemayehu Desalegne Hailu, Gessessew Bugssa Hailu, Alex Hakuzimana, Randah Ribhi Hamadeh, Mitiku Teshome Hambisa, Samer Hamidi, Mouhanad Hammami, Graeme J Hankey, Yuantao Hao, Hilda L Harb, Habtamu Abera Hareri, Josep Maria Haro, Mohammad Sadegh Hassanvand, Rasmus Havmoeller, Roderick J Hay, Simon I Hay, Delia Hendrie, Ileana Beatriz Heredia-Pi, Hans W Hoek, Masako Horino, Nobuyuki Horita, H Dean Hosgood, Aung Soe Htet, Guoqing Hu, Hsiang Huang, John J Huang, Bethany M Huntley, Chantal Huynh, Kim Moesgaard Iburg, Bogdan Vasile Ileanu, Kaire Innos, Asnake Ararsa Irenso, Nader Jahanmehr, Mihajlo B Jakovljevic, Peter James, Spencer Lewis James, Mehdi Javanbakht, Sudha P Jayaraman, Achala Upendra Jayatilleke, Panniyammakal Jeemon, Vivekanand Jha, Denny John, Catherine Johnson, Sarah C Johnson, Jost B Jonas, Knud Juel, Zubair Kabir, Yogeshwar Kalkonde, Ritul Kamal, Haidong Kan, Andre Karch, Corine Kakizi Karema, Seyed M Karimi, Amir Kasaeian, Nicholas J Kassebaum, Anshul Kastor, Srinivasa Vittal Katikireddi, Konstantin Kazanjan, Peter Njenga Keiyoro, Laura Kemmer, Andrew Haddon Kemp, Andre Pascal Kengne, Amene Abebe Kerbo, Maia Kereselidze, Chandrasekharan Nair Kesavachandran, Yousef Saleh Khader, Ibrahim Khalil, Abdur Rahman Khan, Ejaz Ahmad Khan, Gulfaraz Khan, Young-Ho Khang, Abdullah Tawfih Abdullah Khoja, Irma Khonelidze, Jagdish Khubchandani, Getiye Dejenu Kibret, Daniel Kim, Pauline Kim, Yun Jin Kim, Ruth W Kimokoti, Yohannes Kinfu, Niranjan Kissoon, Miia Kivipelto, Yoshihiro Kokubo, Anneli Kolk, Dhaval Kolte, Jacek A Kopec, Soewarta Kosen, Parvaiz A Koul, Ai Koyanagi, Michael Kravchenko, Sanjay Krishnaswami, Kristopher J Krohn, Barthelemy Kuate Defo, Burcu Kucuk Bicer, Ernst J Kuipers, Veena S Kulkarni, G Anil Kumar, Fekede Asefa Kumsa, Michael Kutz, Hmwe H Kyu, Anton Carl Jonas Lager, Aparna Lal, Dharmesh Kumar Lal, Ratilal Lalloo, Tea Lallukka, Qing Lan, Sinead M Langan, Van C Lansingh, Heidi J Larson, Anders Larsson, Dennis Odai Laryea, Asma Abdul Latif, Alicia Elena Beatriz Lawrynowicz, Janet L Leasher, James Leigh, Mall Leinsalu, Cheru Tesema Leshargie, Janni Leung, Ricky Leung, Miriam Levi, Xiaofeng Liang, Stephen S Lim, Margaret Lind, Shai Linn, Steven E Lipshultz, Patrick Liu, Yang Liu, Loon-Tzian Lo, Giancarlo Logroscino, Alan D Lopez, Scott A Lorch, Paulo A Lotufo, Rafael Lozano, Raimundas Lunevicius, Ronan A Lyons, Erlyn Rachelle King Macarayan, Mark T Mackay, Hassan Magdy Abd El Razek, Mohammed Magdy Abd El Razek, Mahdi Mahdavi, Azeem Majeed, Reza Malekzadeh, Deborah Carvalho Malta, Lorenzo G Mantovani, Tsegahun Manyazewal, Chabila C Mapoma, Wagner Marcenes, Guy B Marks, Neal Marquez, Jose Martinez-Raga, Melvin Barrientos Marzan, João Massano, Manu Raj Mathur, Pallab K Maulik, Mohsen Mazidi, Colm McAlinden, John J McGrath, Claire McNellan, Peter A Meaney, Alem Mehari, Man Mohan Mehndiratta, Toni Meier, Alemayehu B Mekonnen, Kidanu Gebremariam Meles, Ziad A Memish, Melkamu Merid Mengesha, Desalegn Tadese Mengiste, Mubarek Abera Mengistie, Bereket Gebremichael Menota, George A Mensah, Seid Tiku Mereta, Atte Meretoja, Tuomo J Meretoja, Haftay Berhane Mezgebe, Renata Micha, Anoushka Millear, Edward J Mills, Shawn Minnig, Mojde Mirarefin, Erkin M Mirrakhimov, Charles N Mock, Karzan Abdulmuhsin Mohammad, Shafiu Mohammed, Sanjay K Mohanty, Ali H Mokdad, Glen Liddell D Mola, Mariam Molokhia, Lorenzo Monasta, Marcella Montico, Maziar Moradi-Lakeh, Paula Moraga, Lidia Morawska, Rintaro Mori, Mark Moses, Ulrich O Mueller, Srinivas Murthy, Kamarul Imran Musa, Jean B Nachega, Chie Nagata, Gabriele Nagel, Mohsen Naghavi, Aliya Naheed, Luigi Naldi, Vinay Nangia, Bruno Ramos Nascimento, Ionut Negoi, Sudan Prasad Neupane, Charles R Newton, Marie Ng, Frida Namnyak Ngalesoni, Josephine Wanjiku Ngunjiri, Grant Nguyen, Dina Nur Anggraini Ningrum, Sandra Nolte, Marika Nomura, Ole F Norheim, Bo Norrving, Jean Jacques N Noubiap, Carla Makhlouf Obermeyer, Felix Akpojene Ogbo, In-Hwan Oh, Anselm Okoro, Olanrewaju Oladimeji, Andrew Toyin Olagunju, Pedro R Olivares, Helen E Olsen, Bolajoko Olubukunola Olusanya, Jacob Olusegun Olusanya, John Nelson Opio, Eyal Oren, Alberto Ortiz, Richard H Osborne, Majdi Osman, Mayowa O Owolabi, Mahesh PA, Amanda W Pain, Smita Pakhale, Elizabeth Palomares Castillo, Adrian Pana, Christina Papachristou, Mahboubeh Parsaeian, Tejas Patel, George C Patton, Deepak Paudel, Vinod K Paul, Neil Pearce, David M Pereira, Rogelio Perez-Padilla, Fernando Perez-Ruiz, Norberto Perico, Konrad Pesudovs, Max Petzold, Michael Robert Phillips, David M Pigott, Julian David Pillay, Christine Pinho, Suzanne Polinder, Constance D Pond, V Prakash, Manorama Purwar, Mostafa Qorbani, D Alex Quistberg, Amir Radfar, Anwar Rafay, Kazem Rahimi, Vafa Rahimi-Movaghar, Mahfuzar Rahman, Mohammad Hifz Ur Rahman, Rajesh Kumar Rai, Usha Ram, Saleem M Rana, Zane Rankin, Paturi Vishnupriya Rao, Puja C Rao, Salman Rawaf, Maria Albertina Santiago Rego, Marissa Reitsma, Giuseppe Remuzzi, Andre M N N Renzaho, Serge Resnikoff, Satar Rezaei, Mohammad Sadegh Rezai, Antonio L Ribeiro, Hirbo Shore Roba, Mohammad Bagher Rokni, Luca Ronfani, Gholamreza Roshandel, Gregory A Roth, Dietrich Rothenbacher, Nawal K Roy, Perminder S Sachdev, Ben Benasco Sackey, Mohammad Yahya Saeedi, Saeid Safiri, Rajesh Sagar, Mohammad Ali Sahraian, Muhammad Muhammad Saleh, Joshua A Salomon, Abdallah M Samy, Juan Ramon Sanabria, Maria Dolores Sanchez-Niño, Logan Sandar, Itamar S Santos, João Vasco Santos, Milena M Santric Milicevic, Rodrigo Sarmiento-Suarez, Benn Sartorius, Maheswar Satpathy, Miloje Savic, Monika Sawhney, Mete I Saylan, Ben Schöttker, Aletta E Schutte, David C Schwebel, Soraya Seedat, Abdulbasit Musa Seid, Canaan Negash Seifu, Sadaf G Sepanlou, Berrin Serdar, Edson E Servan-Mori, Tesfaye Setegn, Katya Anne Shackelford, Amira Shaheen, Saeid Shahraz, Masood Ali Shaikh, Marina Shakh-Nazarova, Mansour Shamsipour, Sheikh Mohammed Shariful Islam, Jayendra Sharma, Rajesh Sharma, Jun She, Sara Sheikhbahaei, Jiabin Shen, Peilin Shi, Mika Shigematsu, Min-Jeong Shin, Rahman Shiri, Haitham Shoman, Mark G Shrime, Ephrem Lejore Sibamo Sibamo, Inga Dora Sigfusdottir, Diego Augusto Santos Silva, Dayane Gabriele Alves Silveira, Shireen Sindi, Abhishek Singh, Jasvinder A Singh, Om Prakash Singh, Prashant Kumar Singh, Virendra Singh, Abiy Hiruye Sinke, Aklilu Endalamaw Sinshaw, Vegard Skirbekk, Karen Sliwa, Alison Smith, Eugene Sobngwi, Samir Soneji, Joan B Soriano, Tatiane Cristina Moraes Sousa, Luciano A Sposato, Chandrashekhar T Sreeramareddy, Vasiliki Stathopoulou, Nicholas Steel, Caitlyn Steiner, Sabine Steinke, Mark Andrew Stokes, Saverio Stranges, Mark Strong, Konstantinos Stroumpoulis, Lela Sturua, Muawiyyah Babale Sufiyan, Rizwan Abdulkader Suliankatchi, Jiandong Sun, Patrick Sur, Soumya Swaminathan, Bryan L Sykes, Rafael Tabarés-Seisdedos, Karen M Tabb, Getachew Redae Taffere, Roberto Tchio Talongwa, Musharaf Tarajia, Mohammad Tavakkoli, Nuno Taveira, Stephanie Teeple, Teketo Kassaw Tegegne, Arash Tehrani-Banihashemi, Tesfalidet Tekelab, Dejen Yemane Tekle, Girma Temam Shifa, Abdullah Sulieman Terkawi, Azeb Gebresilassie Tesema, JS Thakur, Alan J Thomson, Taavi Tillmann, Tenaw Yimer Tiruye, Ruoyan Tobe-Gai, Marcello Tonelli, Roman Topor-Madry, Miguel Tortajada, Christopher Troeger, Thomas Truelsen, Abera Kenay Tura, Uche S Uchendu, Kingsley N Ukwaja, Eduardo A Undurraga, Chigozie Jesse Uneke, Olalekan A Uthman, Job F M van Boven, Rita Van Dingenen, Santosh Varughese, Tommi Vasankari, Narayanaswamy Venketasubramanian, Francesco S Violante, Sergey K Vladimirov, Vasiliy Victorovich Vlassov, Stein Emil Vollset, Theo Vos, Joseph A Wagner, Tolassa Wakayo, Stephen G Waller, Judd L Walson, Haidong Wang, Yuan-Pang Wang, David A Watkins, Elisabete Weiderpass, Robert G Weintraub, Chi-Pang Wen, Andrea Werdecker, Joshua Wesana, Ronny Westerman, Harvey A Whiteford, James D Wilkinson, Charles Shey Wiysonge, Belete Getahun Woldeyes, Charles D A Wolfe, Sungho Won, Abdulhalik Workicho, Shimelash Bitew Workie, Mamo Wubshet, Denis Xavier, Gelin Xu, Ajit Kumar Yadav, Mohsen Yaghoubi, Bereket Yakob, Lijing L Yan, Yuichiro Yano, Mehdi Yaseri, Hassen Hamid Yimam, Paul Yip, Naohiro Yonemoto, Seok-Jun Yoon, Mustafa Z Younis, Chuanhua Yu, Zoubida Zaidi, Maysaa El Sayed Zaki, Carlos Zambrana-Torrelio, Tomas Zapata, Zerihun Menlkalew Zenebe, Sanjay Zodpey, Leo Zoeckler, Liesl Joanna Zuhlke, Christopher J L Murray

## Abstract

**Background:**

National levels of personal health-care access and quality can be approximated by measuring mortality rates from causes that should not be fatal in the presence of effective medical care (ie, amenable mortality). Previous analyses of mortality amenable to health care only focused on high-income countries and faced several methodological challenges. In the present analysis, we use the highly standardised cause of death and risk factor estimates generated through the Global Burden of Diseases, Injuries, and Risk Factors Study (GBD) to improve and expand the quantification of personal health-care access and quality for 195 countries and territories from 1990 to 2015.

**Methods:**

We mapped the most widely used list of causes amenable to personal health care developed by Nolte and McKee to 32 GBD causes. We accounted for variations in cause of death certification and misclassifications through the extensive data standardisation processes and redistribution algorithms developed for GBD. To isolate the effects of personal health-care access and quality, we risk-standardised cause-specific mortality rates for each geography-year by removing the joint effects of local environmental and behavioural risks, and adding back the global levels of risk exposure as estimated for GBD 2015. We employed principal component analysis to create a single, interpretable summary measure–the Healthcare Quality and Access (HAQ) Index–on a scale of 0 to 100. The HAQ Index showed strong convergence validity as compared with other health-system indicators, including health expenditure per capita (*r*=0·88), an index of 11 universal health coverage interventions (*r*=0·83), and human resources for health per 1000 (*r*=0·77). We used free disposal hull analysis with bootstrapping to produce a frontier based on the relationship between the HAQ Index and the Socio-demographic Index (SDI), a measure of overall development consisting of income per capita, average years of education, and total fertility rates. This frontier allowed us to better quantify the maximum levels of personal health-care access and quality achieved across the development spectrum, and pinpoint geographies where gaps between observed and potential levels have narrowed or widened over time.

**Findings:**

Between 1990 and 2015, nearly all countries and territories saw their HAQ Index values improve; nonetheless, the difference between the highest and lowest observed HAQ Index was larger in 2015 than in 1990, ranging from 28·6 to 94·6. Of 195 geographies, 167 had statistically significant increases in HAQ Index levels since 1990, with South Korea, Turkey, Peru, China, and the Maldives recording among the largest gains by 2015. Performance on the HAQ Index and individual causes showed distinct patterns by region and level of development, yet substantial heterogeneities emerged for several causes, including cancers in highest-SDI countries; chronic kidney disease, diabetes, diarrhoeal diseases, and lower respiratory infections among middle-SDI countries; and measles and tetanus among lowest-SDI countries. While the global HAQ Index average rose from 40·7 (95% uncertainty interval, 39·0–42·8) in 1990 to 53·7 (52·2–55·4) in 2015, far less progress occurred in narrowing the gap between observed HAQ Index values and maximum levels achieved; at the global level, the difference between the observed and frontier HAQ Index only decreased from 21·2 in 1990 to 20·1 in 2015. If every country and territory had achieved the highest observed HAQ Index by their corresponding level of SDI, the global average would have been 73·8 in 2015. Several countries, particularly in eastern and western sub-Saharan Africa, reached HAQ Index values similar to or beyond their development levels, whereas others, namely in southern sub-Saharan Africa, the Middle East, and south Asia, lagged behind what geographies of similar development attained between 1990 and 2015.

**Interpretation:**

This novel extension of the GBD Study shows the untapped potential for personal health-care access and quality improvement across the development spectrum. Amid substantive advances in personal health care at the national level, heterogeneous patterns for individual causes in given countries or territories suggest that few places have consistently achieved optimal health-care access and quality across health-system functions and therapeutic areas. This is especially evident in middle-SDI countries, many of which have recently undergone or are currently experiencing epidemiological transitions. The HAQ Index, if paired with other measures of health-system characteristics such as intervention coverage, could provide a robust avenue for tracking progress on universal health coverage and identifying local priorities for strengthening personal health-care quality and access throughout the world.

**Funding:**

Bill & Melinda Gates Foundation.

## Introduction

Quantifying how much personal health care can improve population health and ultimately health-system performance is a crucial undertaking, particularly following the inclusion of universal health coverage (UHC) in the Sustainable Development Goals (SDGs).^1^ Mortality from causes considered amenable to personal health care serve as an important proxy of health-care access and quality (panel),^4^, ^6^, ^7^, ^8^ and thus can be used to benchmark dimensions of health-system performance and to identify untapped potential for advancing personal health-care access and quality.^9^, ^10^, ^11^, ^12^ Much debate exists concerning the relative contributions of personal health care, population-level health initiatives, and social determinants to population health.^13^, ^14^, ^15^, ^16^ Studies show that access to high-quality health care substantially improves many health outcomes, including infectious diseases (eg, tuberculosis and measles);^17^, ^18^, ^19^ maternal and neonatal disorders;^20^, ^21^ several cancers (eg, testicular, skin, and cervical cancers);^22^, ^23^ and many non-communicable diseases (NCDs) such as cerebrovascular disease (stroke),^24^ diabetes,^25^ and chronic kidney disease.^26^ Consequently, assessing mortality rates from these conditions, which are considered amenable to personal health care,^4^, ^6^, ^7^, ^8^ can provide vital insights into access to and quality of health care worldwide. Assessments of both mortality and disease burden attributable to risk factors modifiable through public health programmes and policy (eg, tobacco taxation), combined with access to high-quality personal health care, can provide a more complete picture of the potential avenues for health improvement.

Panel **Context and definitions**

With the present analysis, we use the Global Burden of Diseases, Injuries, and Risk Factors Study (GBD) to approximate average levels of personal health-care access and quality for 195 countries and territories from 1990 to 2015. Here we define key concepts frequently used in the literature focused on assessing health-care quality and how they relate to GBD terminology:

**Avertable burden** refers to disease burden that could be avoided in the presence of high-quality personal health care in addition to disease burden that could be prevented through effective public health (ie, non-personal) interventions.

**Amenable burden** entails disease burden that could be avoided in the presence of high-quality personal health care.^2^, ^3^ To be considered a cause amenable to personal health care, effective interventions must exist for the disease.^4^ The most widely used and cited list of causes amenable to health care is that of Nolte and McKee.

**Preventable burden** involves disease burden that could be avoided through public health programmes or policies focused on wider determinants of health, such as behavioural and lifestyle influences, environmental factors, and socioeconomic status.^2^, ^3^ For some causes, both personal health care and public health programmes and policies can reduce burden.

Within the GBD framework, we have two related terms: attributable and avoidable burden.^5^

**Attributable burden** refers to the difference in disease burden observed at present and burden that would have been observed in a population if past exposure was at the lowest level of risk.

**Avoidable burden** concerns the reduction in future disease burden if observed levels of risk factor exposure today were decreased to a counterfactual level.

For this study, we use the definition of amenable burden and focus on amenable mortality to provide a signal on approximate average levels of national personal health-care access and quality. Future analyses facilitated through the GBD study aim to provide more comprehensive assessments of health systems using amenable burden and preventable burden.

**Garbage codes** refer to causes certified by physicians on death certificates that cannot or should not be considered the actual underlying causes of death. Examples include risk factors like hypertension, non-fatal conditions like yellow nails, and causes that are on the final steps of a disease pathway (eg, certifying cardiopulmonary arrest as the cause when ischaemic heart disease is the true underlying cause of death). A vital strength of the GBD Study is its careful identification of garbage codes by cause, over time, and across locations, and subsequent redistribution to underlying causes based on the GBD cause list.

**Risk-standardisation** involves removing the joint effects of environmental and behavioural risk exposure on cause-specific mortality rates at the country or territory level for each year of analysis, and then adding back the global average of environmental and behavioural risk exposure for every geography-year. The goal of risk-standardisation is to eliminate geographic or temporal differences in cause-specific mortality due to variations in risk factors that are not immediately targeted by personal health care—and thus provide comparable measures of outcomes amenable to personal health-care access and quality over place and time.

**Frontier analysis** refers to the approach used for ascertaining the highest achieved values on the Healthcare Access and Quality Index (HAQ Index) on the basis of development status, as measured by the Socio-demographic Index (SDI). The HAQ Index frontier delineates the maximum HAQ Index reached by a location as it relates to SDI; if a country or territory falls well below the frontier value given its level SDI, this finding suggests that greater gains in personal health-care access and quality should be possible based on the country or territory's place on the development spectrum.

**Research in context**

**Evidence before this study**

In the last several decades, various studies have used measures of amenable mortality, or deaths that could be avoided in the presence of high-quality personal health care, to garner signals about health-system delivery, effectiveness, and performance. Rutstein and colleagues developed an initial list of conditions from which death was “unnecessary and untimely” during the late 1970s, while Charlton and colleagues were the first to apply this concept to population-level analyses in England and Wales. Although variations of amenable cause lists exist today, the most widely used cause list of 33 conditions was developed and further honed by Nolte and McKee during the early-to-mid 2000s. Such analyses of health-care access and quality, as approximated by amenable mortality, have been limited to Europe, Organisation for Economic Co-operation and Development (OECD) countries, and country-specific assessments, including the USA, Australia, and New Zealand. These studies acknowledge several methodological challenges that may impede the policy utility and applications of their results. Heterogeneity in cause of death certification and misclassification, even for countries with complete vital registration systems, can hinder comparability of results over time and place. Further, researchers commonly acknowledge that variations in measured amenable mortality rates may be more reflective of differences in underlying risk factor exposure rather than true differences in personal health-care access and quality.

**Added value of this study**

The Global Burden of Diseases, Injuries, and Risk Factors Study (GBD) provides an appropriate analytic framework through which these main challenges in approximating personal health-care access and quality can be addressed. First, the extensive cause of death data processing and standardisation that occur within GBD allow for the systematic identification and redress of cause of death certification errors or misclassification. These adjustments are conducted across all geographies and over time, accounting for known misclassification patterns and applying well established redistribution algorithms for causes designated to so-called garbage codes, or causes of death that could not or should not be classified as underlying causes of death. Second, we draw on GBD's comparative risk assessment analyses to risk-standardise national cause-specific mortality rates to global levels of risk exposure; this step helps to remove variations in death rates due to risk exposure rather than differences in personal health-care access and quality. Third, we construct the Healthcare Access and Quality (HAQ) Index based on risk-standardised cause-specific death rates to facilitate comparisons over time and by geography. Finally, we produced a HAQ Index frontier to enable a better understanding of the maximum observed levels of the HAQ Index across the development spectrum, and what untapped potential for improving personal health-care access and quality may exist given a country or territory's current resources.

**Implications of all the available evidence**

Our results point to substantive gains for advancing personal health-care access and quality throughout the world since 1990. However, the gap between places with the highest and lowest HAQ Index in 1990 increased by 2015, suggesting that geographic inequalities in personal health-care access and quality might be on the rise. In 2015, countries in western Europe generally had the highest HAQ Index values while geographies in sub-Saharan Africa and Oceania mainly saw the lowest, further emphasising these disparities. A number of countries achieved improvements in the HAQ Index that exceeded the average found for their development level, identifying possible success stories in markedly advancing personal health-care access and quality at the national level. Based on our frontier analysis, many countries and territories currently experience untapped potential for improving health-care access and quality, on the basis of their development, a finding that could be transformative for prioritising particular health-sector reforms, pinpointing cause-specific therapeutic areas that require more policy attention, and monitoring overall progress toward universal health coverage.

In the late 1970s, Rutstein and colleagues first introduced the idea of “unnecessary, untimely deaths”, proposing a list of causes from which death should not occur with timely and effective medical care.^6^ Eventually termed “amenable mortality”, this approach has been modified and extended since, with researchers refining the list of included conditions by accounting for advances in medical care, the introduction of new interventions, and improved knowledge of cause-specific epidemiology.^7^, ^8^, ^27^, ^28^, ^29^ Numerous studies have subsequently assessed amenable mortality trends over time, by sex, and across ages in different populations;^2^, ^10^, ^11^, ^30^, ^31^, ^32^, ^33^ examples include analyses showing variations in amenable mortality within the European Union and Organisation for Economic Co-operation and Development (OECD),^3^, ^34^ and how much the US health system has lagged behind other higher-income countries.^30^, ^31^ Some studies also extended the set of amenable conditions to include those targeted by public health programmes.^31^ The most widely cited and utilised list of causes amenable to personal health care is that of Nolte and McKee,^4^ which has been extensively used in Europe, the USA, and other OECD countries.^9^, ^11^, ^30^, ^31^, ^35^

Previously, several technical challenges have emerged concerning the quantification of mortality from conditions amenable to personal health care and its use for understanding overall health-care access and quality. First, discrepancies in cause of death certification practices and misclassification over time and across geographies affect comparisons of amenable mortality.^4^, ^36^ Second, observed geographic and temporal variations in deaths from selected amenable causes (eg, stroke and heart disease) might be attributed partly differences in risk factor exposure (eg, diet, high BMI, and physical activity) rather than actual differences in access to quality personal health care. Public health programmes and policies might modify these risks in well-functioning health systems, but risk variation can still confound the measurement of personal health-care access and quality. Third, much of this work has occurred in higher-income settings, with few studies applying the concept of amenable mortality as a mechanism for assessing access and quality to personal health care in lower-resource settings. Other critiques involve weak correlations between observed trends and variations in amenable mortality and indicators of health-care provision and spending, although this result could occur if health-care quality is heterogeneous within countries.^37^, ^38^, ^39^, ^40^ Additionally, existing lists might exclude causes for which health care can avert death, such as the effects of trauma care on various injuries,^4^, ^41^, ^42^ and the ages at which personal health care can reduce mortality, namely beyond the age of 75.^43^, ^44^

The goal of this analysis is to use estimates of mortality amenable to personal health care from the Global Burden of Diseases, Injuries, and Risk Factors Study 2015 (GBD 2015) to approximate access to and quality of personal health care in 195 countries and territories from 1990 to 2015. Quantifying access to and quality of personal health care has many policy uses, and no consistent measures of personal health-care access and quality currently list across the development spectrum; for instance, the World Bank coverage index only includes three interventions,^45^ and the 2010–11 International Labour Organization's indicator of formal health coverage covered 93 countries, with substantial data missingness for sub-Saharan Africa.^46^ The highly standardised cause of death estimates generated through GBD,^47^ along with risk factor exposure,^48^ can address several limitations associated with previous studies of amenable mortality. GBD provides comprehensive, comparable estimates of cause-specific death rates by geography, year, age, and sex through its extensive data correction processes to account for variations in cause of death certification.^47^ The quantification of risk exposure and risk-attributable deaths due to 79 risk factors through GBD allows us to account for variations in risk exposure across geographies and time,^48^ and thus helps to isolate variations in death rates due to personal health-care access and quality. We also examine the relationship between our measure of health-care access and quality, as defined by risk-standardised mortality rates amenable to health care, across development levels, as reflected by the Socio-demographic Index (SDI). Finally, we produce a frontier of maximum levels of personal health-care access and quality observed on the basis of SDI, which allows us to quantify the potential for further improvement in relation to development status.

## Methods

### Overview

We employed the most widely cited and used framework for assessing mortality amenable to personal health care.^4^, ^9^, ^11^, ^30^, ^31^, ^35^ The Nolte and McKee cause list does not include all possible causes for which health care can improve survival; however, it does provide a set of conditions for which there is a reasonable consensus that personal health care has a major effect (table 1). Starting with this list, our analysis followed four steps: mapping the Nolte and McKee cause list to GBD causes; risk-standardising mortality rates to remove variations in death rates not easily addressed through personal health care; computing a summary measure of personal health-care access and quality using principal component analysis (PCA); and assessing the highest recorded levels of health-care access and quality across the development spectrum.

**Table 1:** Causes for which mortality is amenable to health care mapped to GBD 2015 causes

		**Amenable age range (years)**
**Communicable, maternal, neonatal, and nutritional diseases**		
Tuberculosis		0–74
Diarrhoea, lower respiratory, and other common infectious diseases		
	Diarrhoeal diseases	0–14
	Lower respiratory infections	0–74
	Upper respiratory infections	0–74
	Diphtheria	0–74
	Whooping cough	0–14
	Tetanus	0–74
	Measles	1–14
Maternal disorders		0–74
Neonatal disorders		0–74
**Non-communicable diseases**		
Neoplasms		
	Colon and rectum cancer	0–74
	Non-melanoma skin cancer (squamous-cell carcinoma)	0–74
	Breast cancer	0–74
	Cervical cancer	0–74
	Uterine cancer	0–44
	Testicular cancer	0–74
	Hodgkin's lymphoma	0–74
	Leukaemia	0–44
Cardiovascular diseases		
	Rheumatic heart disease	0–74
	Ischaemic heart disease	0–74
	Cerebrovascular disease	0–74
	Hypertensive heart disease	0–74
Chronic respiratory diseases		1–14
Digestive diseases		
	Peptic ulcer disease	0–74
	Appendicitis	0–74
	Inguinal, femoral, and abdominal hernia	0–74
	Gallbladder and biliary diseases	0–74
Neurological disorders		
	Epilepsy	0–74
Diabetes, urogenital, blood, and endocrine diseases		
	Diabetes mellitus	0–49
	Chronic kidney disease	0–74
Other non-communicable diseases		
	Congenital heart anomalies	0–74
**Injuries**		
Unintentional injuries		
	Adverse effects of medical treatment	0–74

The age groups for which mortality is regarded as amenable to health care are listed. Causes are ordered on the basis of the GBD cause list and corresponding cause group hierarchies. GBD=Global Burden of Disease.

This study draws from GBD 2015 results; further detail on GBD 2015 data and methods are available elsewhere.^47^, ^48^, ^49^, ^50^ For the present analysis, a vital strength of GBD is its careful evaluation and correction of cause of death certification problems and misclassification at the national level. In the GBD, we systematically identified causes of death that could not or should not be underlying causes of death (so-called garbage codes), and applied established statistical algorithms to correct for and redistribute these deaths.^51^

Our study complies with the Guidelines for Accurate and Transparent Health Estimates Reporting (GATHER);^52^ additional information on the data and modelling strategies used can be found in the appendix.

### Mapping the Nolte and McKee amenable cause list to the GBD cause list

Drawing from Nolte and McKee's list of 33 causes amenable to personal health care,^4^, ^9^, ^11^, ^30^, ^31^, ^35^ we mapped these conditions to the GBD cause list based on corresponding International Classification of Diseases (ICD) codes (appendix p 18). In GBD, thyroid diseases and benign prostatic hyperplasia are part of a larger residual category and thus were excluded. Diphtheria and tetanus are separate causes in GBD so we reported them individually. Because of its extensive processes used to consistently map and properly classify ICD causes over time,^47^, ^53^ GBD supported the assessment of 32 causes on the Nolte and McKee cause list from 1990 to 2015.

### Age-standardised risk-standardised death rates

Some variation in death rates for amenable causes are due to differences in behavioural and environmental risk exposure rather than differences in personal health-care access and quality.^48^, ^54^, ^55^ Using the wide range of risk factors assessed by GBD,^48^ we risk-standardised death rates to the global level of risk exposure.^48^ We did not risk-standardise for variations in metabolic risk factors directly targeted by personal health care: systolic blood pressure, total cholesterol, and fasting plasma glucose. For example, stroke deaths due to high systolic blood pressure are amenable to primary care management of hypertension.

To risk-standardise death rates, we removed the joint effects of national behavioural and environmental risk levels calculated in GBD, and added back the global levels of risk exposure:

$${\text{mr}}_{\text{jascy}}={\text{m}}_{\text{jascy}}(\frac{1-JPAF_{jascy}}{1-JPAF_{jasgy}})$$

where *m_jascy_* is the death rate from cause *j* in age *a*, sex *s*, location *c*, and year *y*; *mr_jascy_* is the risk-standardised death rate; *JPAF_jascy_* is the joint population attributable fraction (PAF) for cause *j*, in age *a*, sex *s*, country *c*, and year *y* for all behavioural and environmental risks included in GBD; and *JPAF_jasgy_* is the joint PAF for cause *j*, in age *a*, sex *s*, and year *y* at the global level.

GBD provides joint PAF estimation for multiple risks combined, which takes into account the mediation of different risks through each other. Further detail on joint PAF computation is available in the appendix (pp 5–8).

We used the GBD world population standard to calculate age-standardised risk-standardised death rates from each cause regarded as amenable to health care.^47^ We did not risk-standardise death rates from diarrhoeal diseases as mortality attributable to unsafe water and sanitation was not computed for high-SDI locations; such standardisation could lead to higher risk-standardised death rates in those countries compared with countries where mortality was attributed to unsafe water and sanitation.^48^ With all causes for which no PAFs are estimated in GBD, such as neonatal disorders and testicular cancer, risk-standardised death rates equalled observed death rates.

The effects of risk-standardisation are highlighted by comparing the log of age-standardised mortality rates to the log of age-standardised risk-standardised mortality rates for amenable causes (appendix p 14). For each SDI quintile, many countries had differing levels of age-standardised mortality rates but their risk-standardised mortality rates were similar, demonstrating how underlying local risk exposure can skew measures of mortality amenable to personal health care.

### Construction of the Healthcare Access and Quality Index based on age-standardised risk standardised death rates

To construct the Healthcare Access and Quality (HAQ) Index, we first rescaled the log age-standardised risk-standardised death rate by cause to a scale of 0 to 100 such that the highest observed value from 1990 to 2015 was 0 and the lowest was 100. To avoid the effects of fluctuating death rates in small populations on rescaling, we excluded populations less than 1 million population from setting minimum and maximum values. Any location with a cause-specific death rate below the minimum or above the maximum from 1990 to 2015 was set to 100 or 0, respectively.

Because each included cause provided some signal on average levels of personal health-care access and quality, we explored four approaches to construct the HAQ Index: PCA, exploratory factor analysis, arithmetic mean, and geometric mean. Details on these four approaches are in the appendix (pp 7, 8, 21, 22). All four measures were highly correlated, with Spearman's rank order correlations exceeding *r*_s_=0·98. We selected the PCA-derived HAQ Index because it provided the strongest correlations with six other currently available cross-country measures of access to care or health-system inputs (table 2). Three indicators came from the GBD Study 2015: health expenditure per capita, hospital beds per 1000, and the UHC tracer intervention index, a composite measure of 11 UHC tracer interventions (four childhood vaccinations; skilled birth attendance; coverage of at least one and four antenatal care visits; met need for family planning with modern contraception; tuberculosis case detection rates; insecticide-treated net coverage; and antiretroviral therapy coverage for populations living with HIV).^56^ Three indicators came from WHO (physicians, nurses, and midwives per 1000),^57^ the International Labour Organization,^46^ and the World Bank (coverage index based on diphtheria-pertussis-tetanus vaccine coverage, coverage of at least four antenatal care visits, and proportion of children with diarrhoea receiving appropriate treatment).^45^ All indicators had correlation coefficients greater than 0·60, and three exceeded 0·80 (health expenditure per capita, the UHC tracer index, and International Labour Organization formal health coverage).

**Table 2:** Correlations between different constructions of the HAQ Index and existing indicators of health-care access or quality

	**Source and year**	**Geographies represented**	**HAQ Index construction**			
			PCA weighted	EFA weighted	Geometric mean	Mean
Health expenditure per capita	GBD 2015	195	0·884	0·880	0·854	0·864
Hospital beds (per 1000)	GBD 2015	195	0·700	0·683	0·625	0·650
UHC tracer index of 11 interventions	GBD 2015	188	0·826	0·820	0·812	0·818
Physicians, nurses, and midwives per 1000	WHO 2010	73	0·769	0·755	0·725	0·732
Proportion of population with formal health coverage	ILO 2010–11	93	0·808	0·798	0·773	0·781
Coverage index of three primary health-care interventions	World Bank 2015	123	0·601	0·589	0·557	0·570

The universal health coverage tracer index of 11 interventions included coverage of four childhood vaccinations (BCG, measles, three doses of diphtheria-pertussis-tetanus, and three doses of polio vaccines); skilled birth attendance; coverage of at least one and four antenatal care visits; met need for family planning with modern contraception; tuberculosis case detection rates; insecticide-treated net coverage; and antiretroviral therapy coverage for populations living with HIV. The World Bank coverage index included coverage of three interventions: three doses of diphtheria-pertussis-tetanus vaccine; at least four antenatal care visits; and children with diarrhoea receiving appropriate treatment. HAQ Index=Healthcare Access and Quality Index. PCA=principal components analysis. EFA=exploratory factor analysis. GBD=Global Burden of Disease. UHC=universal health coverage. ILO=International Labour Organization.

The appendix (pp 21, 22) provides final rescaled PCA weights derived from the first five components that collectively accounted for more than 80% of the variance in cause-specific measures. Colon and breast cancer had negative PCA weights, which implied higher death rates were associated with better access and quality of care; because this cannot be true we set these weights to zero in the final PCA-derived HAQ Index. The appendix (p 15) compares each geography's HAQ Index in 2015 with the log of its age-standardised risk-standardised mortality rates.

### Quantifying maximum levels of the HAQ Index across the development spectrum

To better understand maximum levels of personal health-care access and quality potentially achievable across the development spectrum, we produced a frontier based on the relationship between the HAQ Index and SDI. We tested both stochastic frontier analysis models and data envelopment analysis; however, the relationship between SDI and the HAQ Index did not fit standard stochastic frontier analysis models,^58^ and data envelopment analysis cannot account for measurement error and is sensitive to outliers.^59^ To generate a frontier fit that closely follows the observed HAQ Index and allowed for measurement error, we used free disposal hull analysis on 1000 bootstrapped samples of the data.^58^ Every bootstrap included a subset of locations produced by randomly sampling (with replacement) from all GBD geographies. The final HAQ Index value was drawn from the uncertainty distribution for each location-year, with outliers removed by excluding super-efficient units; additional methodological detail can be found in the appendix (pp 9–12). Last, we used a Loess regression to produce a smooth frontier for each five-year interval from 1990 to 2015. For every geography, we report the maximum possible HAQ Index value on the basis of SDI in 1990 and 2015, while values for all years can be found in the appendix (pp 23–28).

### Uncertainty analysis

GBD aims to propagate all sources of uncertainty through its estimation process,^47^, ^48^ which results in uncertainty intervals (UIs) accompanying each point estimate of death by cause, geography, year, age group, and sex. We computed the HAQ Index for each geography-year based on 1000 draws from the posterior distribution for each included cause of death. We report 95% UIs based on the ordinal 25th and 975th draws for each quantity of interest.

### Role of the funding source

The funder of the study had no role in study design, data collection, data analysis, data interpretation, or writing of the report. The corresponding author had full access to all the data in the study and had final responsibility for the decision to submit for publication.

## Results

**Figure 1:**
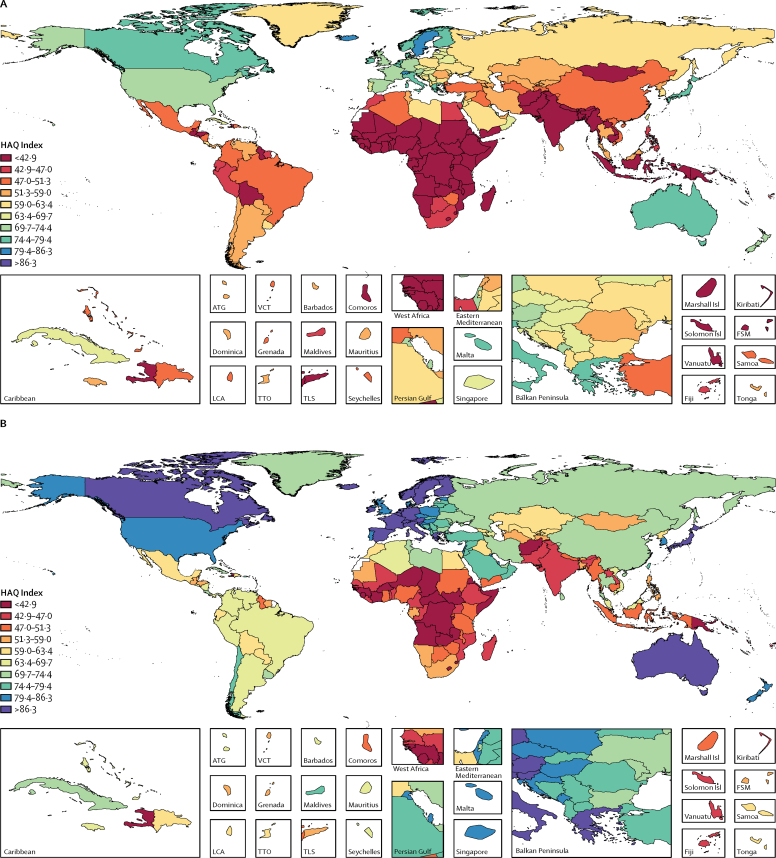
Map of HAQ Index values, by decile, in 1990 (A) and 2015 (B) Deciles were based on the distribution of HAQ Index values in 2015 and then were applied for 1990. HAQ Index = Healthcare Access and Quality Index. ATG=Antigua and Barbuda. VCT=Saint Vincent and the Grenadines. LCA=Saint Lucia. TTO=Trinidad and Tobago. TLS=Timor-Leste. FSM=Federated States of Micronesia.

Distinct geographic patterns emerged for overall HAQ Index levels and gains from 1990 to 2015 (figure 1). Andorra and Iceland had the highest HAQ Index in 1990, whereas most of sub-Saharan Africa and south Asia and several countries in Latin America and the Caribbean were in the first decile. By 2015, nearly all countries and territories saw increases in their HAQ Index, yet the gap between the highest and lowest HAQ Index levels was wider in 2015 (66·0) than in 1990 (61·6). The tenth decile included many countries in western Europe, Canada, Japan, and Australia, while the UK and the USA were in the ninth decile. Latin America and the Caribbean had varied HAQ Index levels, spanning from Haiti (first decile) to Chile (seventh decile). By 2015, Vietnam and Malaysia reached the sixth decile; China and Thailand rose to the seventh decile; and Turkey and several countries in the Middle East and Eastern Europe improved to the eighth decile. In sub-Saharan Africa, Cape Verde (fifth decile), Namibia, South Africa, Gabon, and Mauritania (fourth decile) had the highest HAQ Index levels in 2015, rising from their positions since 1990. At the same time, many sub-Saharan African countries remained in the first decile in 2015, including the Democratic Republic of the Congo, Niger, and Zambia. In Asia and the Pacific, a number of countries also experienced relatively low HAQ Index levels: Afghanistan and Papua New Guinea (first decile); Pakistan and India (second decile); and Indonesia, Cambodia, and Myanmar (third decile).

**Figure 2:**
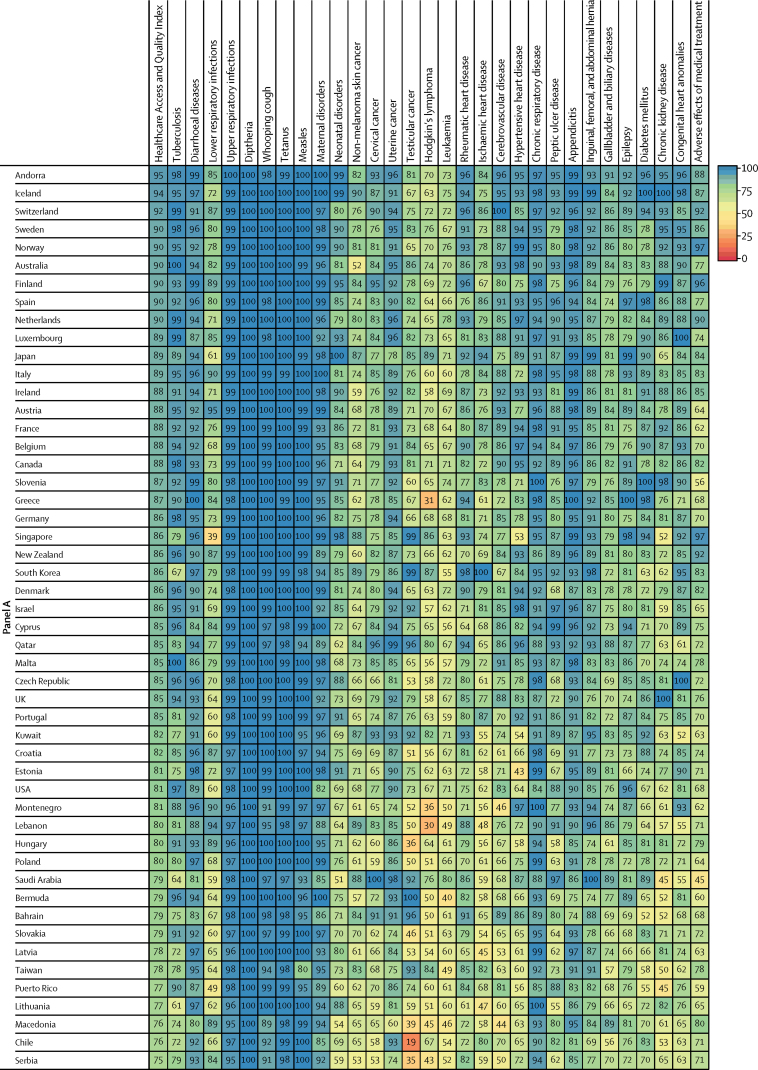

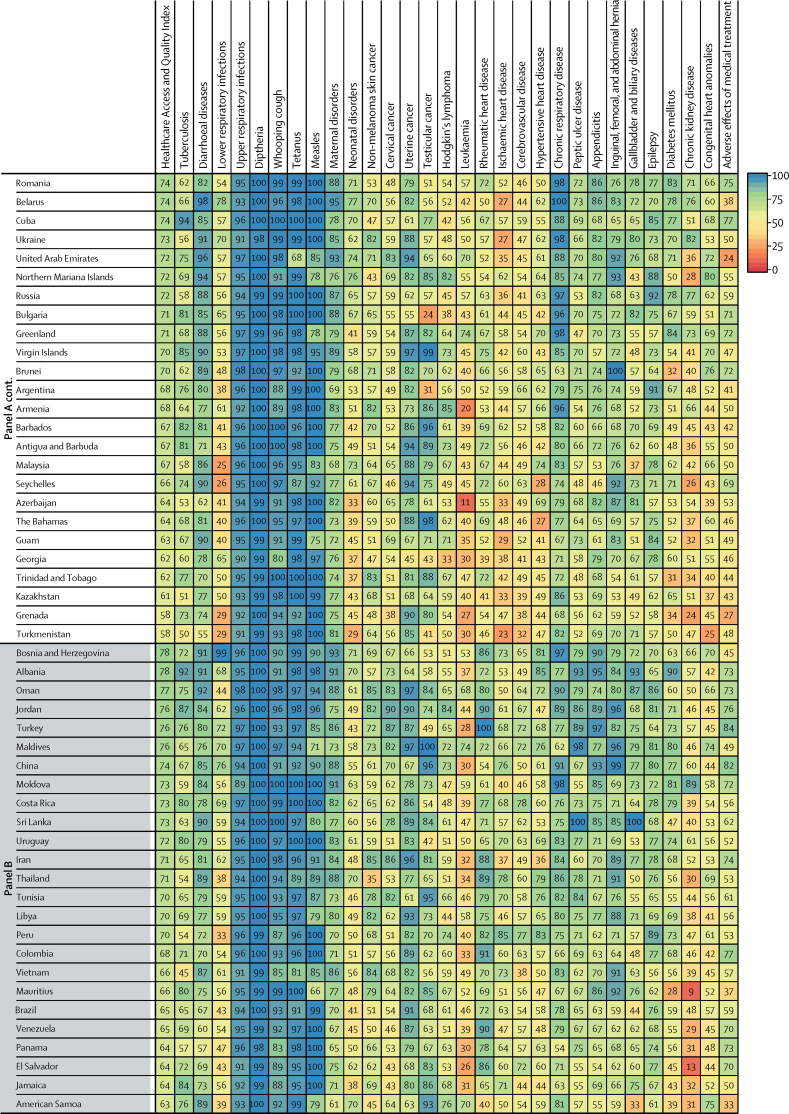

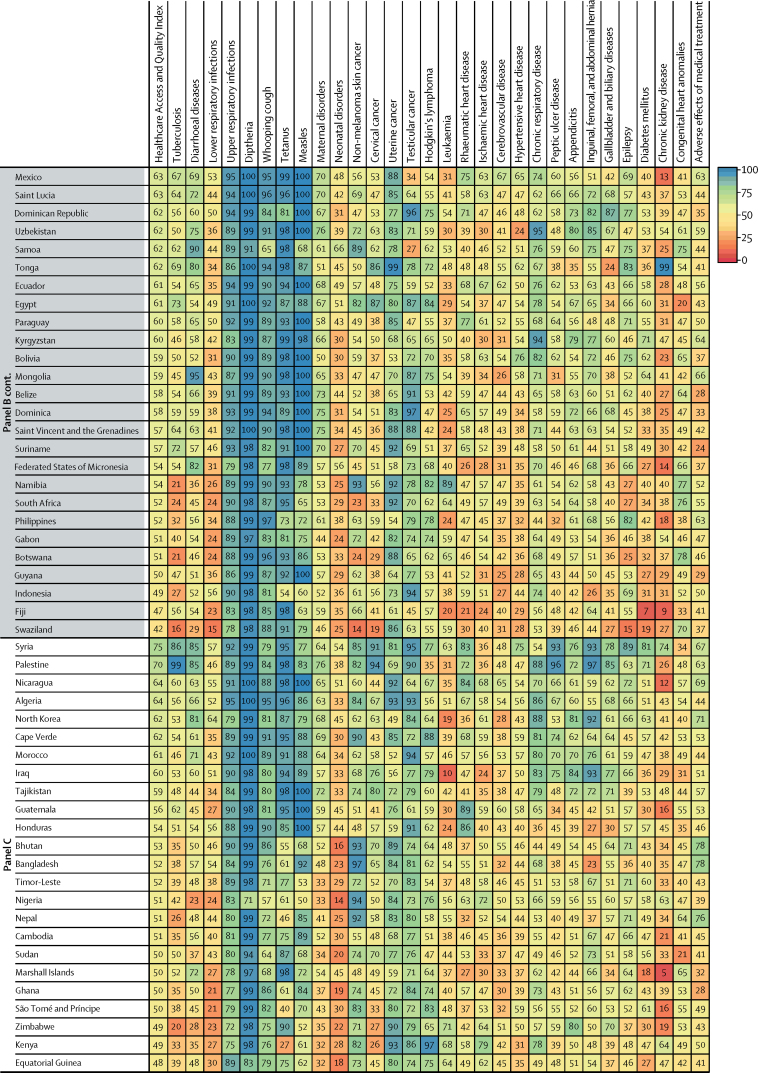

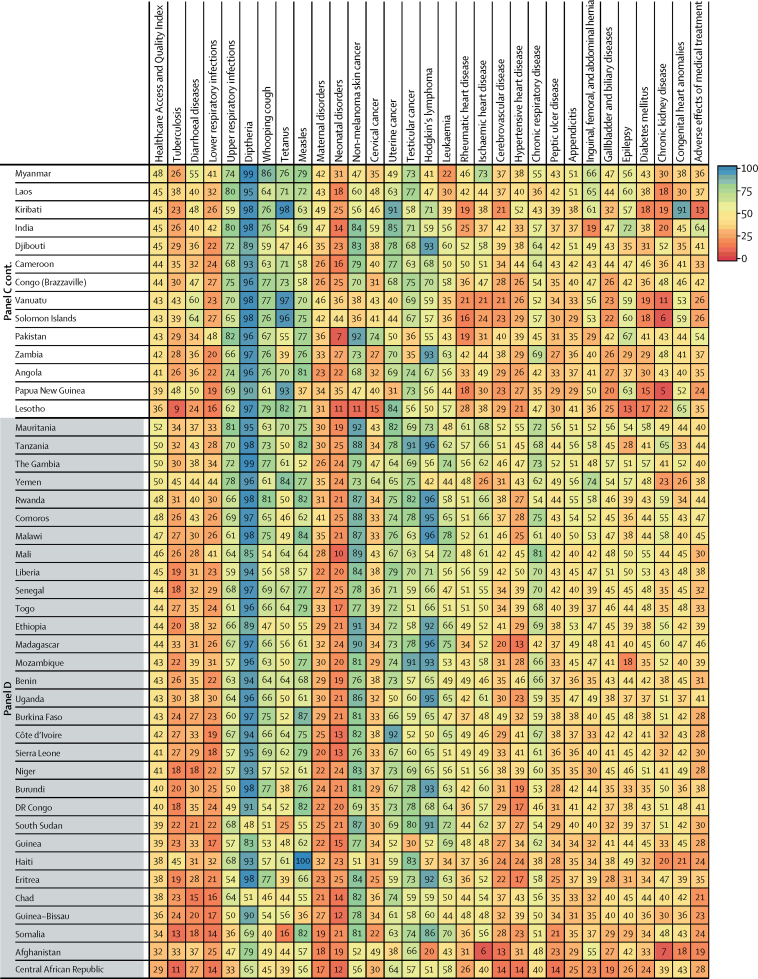
Performance of the HAQ Index and 25 individual causes by the fourth SDI quartile (A), third SDI quartile (B), second SDI quartile (C), and first SDI quartile (D) in 2015 In addition to the HAQ Index, all causes presented in this figure are scaled 0 to 100, with 100 being the “best” value (ie, lowest observed age-standardised risk-standardised mortality rate by cause) and 0 being the “worst value” (ie, highest observed age-standardised risk-standardised mortality rate by cause) between 1990 and 2015. Within each SDI quartile, countries and geographies are ordered by their HAQ Index in 2015. HAQ Index=Healthcare Access and Quality Index. SDI=Socio-demographic Index.

Comparing the overall HAQ Index with its component parts showed substantial heterogeneity in 2015, even within similar SDI quartiles (figure 2). Within the fourth SDI quartile, most geographies performed well on several vaccine-preventable diseases, including measles, diphtheria, tetanus, and whooping cough, yet some experienced lower values for communicable conditions such as lower respiratory infections. Geographies in the fourth SDI quartile generally performed worse for cancers, but many recorded values exceeding 90 for cervical and uterine cancers. Nearly all geographies in the fourth SDI quartile surpassed 90 for maternal disorders, while geographies in the third and second SDI quartiles showed far more diverse results. A similar pattern emerged in causes for which routine surgeries can easily avert mortality (eg, appendicitis and hernias) among third and second SDI quartile geographies, with some countries performing fairly well for such causes (eg, China, Turkey, Sri Lanka) while others lagged behind (eg, Mexico, Indonesia, South Africa). Many geographies in the third and second SDI quartiles not only had fairly low values for NCDs such as diabetes, chronic kidney disease, and hypertensive heart disease, but also fared poorly on a subset of infectious diseases (ie, tuberculosis, lower respiratory infections, and diarrhoeal diseases) and neonatal disorders. In the first SDI quartile, neonatal and maternal disorders, tuberculosis, lower respiratory infections, and diarrhoeal diseases often led to the lowest scaled values, while most geographies experienced relatively better performances for a subset of cancers. Notably, several countries in the first SDI quartile recorded fairly high values for vaccine-preventable diseases. By contrast, nearly all of these countries and territories saw values lower than 50 for causes associated with routine surgeries and more complex case management (eg, epilepsy, diabetes, and chronic kidney disease).

**Figure 3:**
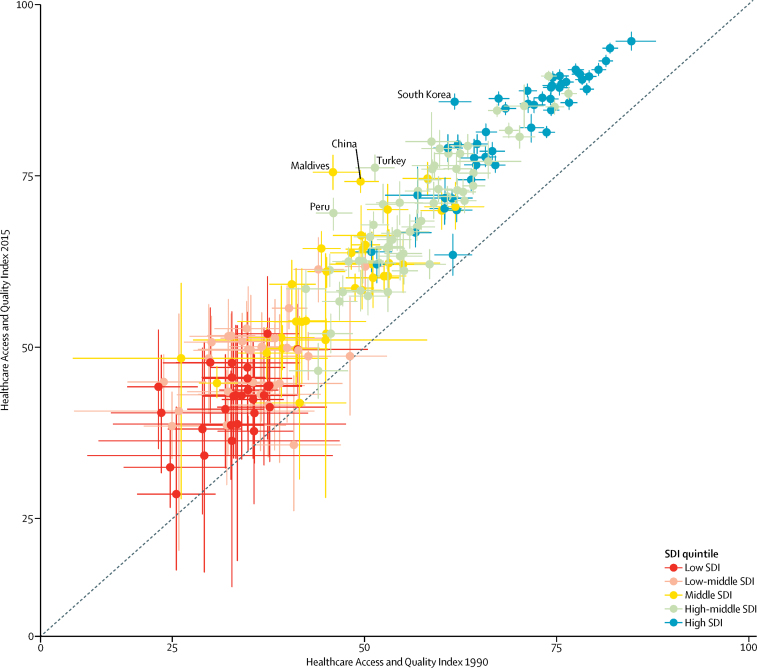
Comparison of 1990 and 2015 HAQ Index estimates, with uncertainty, by country or territory Geographies with the largest improvement in the HAQ Index from 1990 to 2015 are labelled. All countries and territories are colour-coded by SDI quintile in 2015. HAQ Index=Healthcare Access and Quality Index. SDI=Socio-demographic Index.

For nearly all countries and territories, the HAQ Index has markedly improved since 1990, with 167 recording statistically significant increases by 2015 (figure 3). Because of incomplete data systems, uncertainty bounds were relatively large for lower-SDI countries, whereas uncertainty for higher-SDI countries—places where data systems are more complete and of high quality—was much smaller. Five countries with the largest significant increases for the HAQ Index were South Korea (high SDI), Turkey and Peru (high-middle SDI), and China and the Maldives (middle SDI). Among low-middle-SDI and low-SDI countries, Laos and Ethiopia saw among the greatest improvements in the HAQ Index; however, these gains were less pronounced due to wide uncertainty bounds.

**Figure 4:**
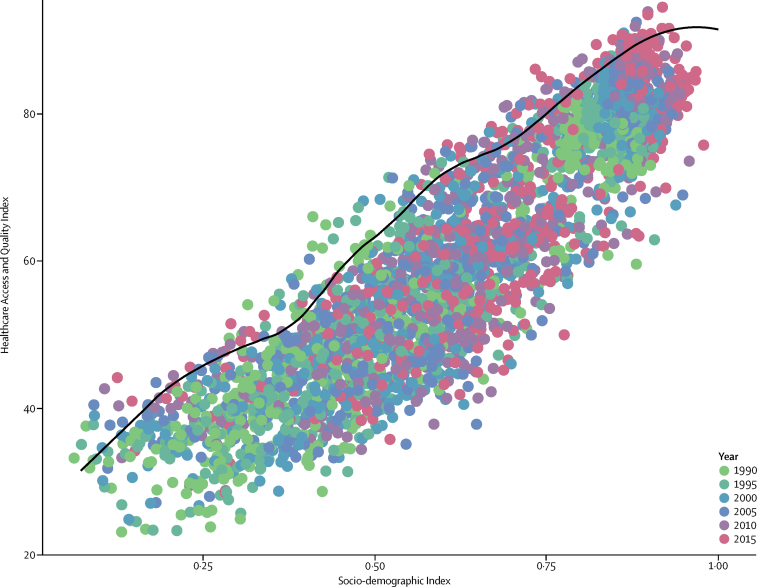
Defining the HAQ Index frontier on the basis of SDI Each circle represents the HAQ Index and level of SDI for a given geography-year, and circles are colour-coded by year from 1990 to 2015. The black line represents the HAQ Index frontier, or the highest observed HAQ Index value, at a given level of SDI across years. HAQ Index=Healthcare Access and Quality Index. SDI=Socio-demographic Index.

Based on a frontier analysis, we found that, as SDI increases, the highest observed HAQ Index values also rose (figure 4). Further, maximum HAQ Index levels achieved generally improved since 1990 across levels of SDI. Table 3 details each geography's HAQ Index values for 5-year intervals from 1990 to 2015, as well as their frontier HAQ Index levels on the basis of a location's SDI. Measuring the distance between a geography's observed HAQ Index in 1990 and 2015 and its frontier for these years provides a benchmark for potential gains in health-care access and quality—a metric that also considers the geography's relative resources on the basis of SDI. Additionally, comparing how differences between a given place's observed HAQ Index and frontier change over time can show where personal health-care access and quality have improved in parallel with changes in development.

**Table 3:** Global, regional, and national or territory-level estimates of the HAQ Index for each 5-year interval from 1990 to 2015, frontier values in 1990 and 2015 on the basis of SDI, and the difference between observed HAQ Index and frontier values in 1990 and 2015

			**HAQ Index (95% UI)**						**HAQ Index frontier**		**Difference between observed and frontier HAQ Index values**	
			1990	1995	2000	2005	2010	2015	1990	2015	1990	2015
Global			40·7 39·0–42·8	42·7 41·0–44·5	44·8 43·2–46·7	47·8 46·3–49·6	51·0 49·5–52·7	53·7 52·2–55·4	61·9	73·8	21·2	20·1
Southeast Asia, east Asia, and Oceania			44·8 42·8–47·1	48·1 46·1–50·3	51·1 49·3–53·0	55·4 53·7–57·3	60·0 58·3–61·7	63·5 61·7–65·4	57·6	75·0	12·8	11·5
	East Asia		49·5 47·5–51·9	53·8 51·8–55·9	57·8 56·0–59·7	63·6 61·8–65·4	69·7 68·0–71·3	73·8 72·2–75·4	57·1	75·4	7·6	1·6
		China	49·5 47·4–51·8	53·7 51·7–55·9	57·8 55·9–59·7	63·7 61·9–65·6	69·9 68·2–71·5	74·2 72·5–75·8	56·2	75·4*	6·7	1·2
		North Korea	53·1 46·2–59·8	55·8 49·5–61·6	56·0 50·2–61·6	57·5 52·8–62·3	60·4 56·0–64·6	62·3 57·2–67·1	70·6	69·1	17·5	6·9
		Taiwan	64·2 62·5–66·4	66·8 65·1–68·8	70·1 68·5–71·9	73·6 72·1–75·4	77·1 75·6–78·6	77·6 74·7–80·4	76·8	88·6	12·6	11·0
	Southeast Asia		38·6 35·8–41·4	40·9 38·4–43·4	43·0 40·7–45·4	46·0 43·7–48·5	49·4 47·1–51·8	52·1 49·5–54·7	59·0	74·0	20·4	21·9
		Cambodia	30·0 26·5–33·6	31·3 28·1–34·9	34·1 30·8–37·4	40·7 37·6–44·0	45·5 42·2–48·5	50·7 47·2–54·6	45·4	62·3	15·3	11·6
		Indonesia	37·2 33·4–41·4	39·7 36·3–43·7	41·3 38·3–44·5	43·8 40·9–46·7	46·8 43·7–49·9	49·2 45·3–52·9	60·6	74·4	23·4	25·2
		Laos	23·8 19·1–29·3	25·7 21·5–30·4	28·7 25·0–32·7	33·6 30·4–36·9	39·6 36·4–42·8	44·9 40·7–48·9	46·3	63·9	22·5	19·0
		Malaysia	54·2 52·2–56·5	57·2 55·3–59·2	60·3 58·5–62·1	63·2 61·5–64·9	63·9 62·3–65·7	66·6 64·1–69·2	69·3	81·5	15·1	14·9
		Maldives	45·9 43·3–49·2	51·6 49·4–54·3	59·3 57·3–61·3	67·1 65·4–69·0	72·9 71·1–74·6	75·5 73·0–78·0	50·1	73·2*	4·3	..
		Mauritius	53·6 51·6–56·1	57·3 55·6–59·1	59·8 58·2–61·8	61·8 60·1–63·5	63·3 61·6–65·1	65·7 64·0–67·5	68·6	79·0	15·0	13·3
		Myanmar	29·7 22·4–37·5	31·6 23·9–39·5	34·7 26·9–43·1	39·5 31·6–48·1	44·4 36·2–52·6	48·4 40·3–56·3	45·8	64·9	16·1	16·5
		Philippines	45·0 43·2–47·1	46·1 44·3–48·2	47·5 45·7–49·5	47·6 45·8–49·6	49·6 47·8–51·5	52·0 49·9–54·2	63·8	74·1	18·8	22·1
		Sri Lanka	56·9 55·2–58·5	59·5 57·5–61·3	60·4 58·3–62·2	63·8 62·0–65·4	68·9 67·5–70·3	72·8 69·5–76·0	66·3	76·8	9·4	4·1
		Seychelles	50·7 48·6–53·2	55·1 52·9–57·4	58·5 56·4–60·6	61·1 59·1–63·1	63·9 61·7–65·7	66·1 63·6–68·5	72·2	80·9	21·5	14·8
		Thailand	52·4 50·1–54·9	55·9 53·7–58·1	59·3 57·3–61·6	64·8 62·6–67·0	68·4 66·1–70·5	70·8 68·0–73·8	66·5	76·8	14·1	5·9
		Timor-Leste	32·2 27·2–39·6	35·4 30·9–41·3	38·2 33·7–45·2	42·6 38·7–48·1	48·4 44·4–53·2	51·6 46·9–57·0	46·6	59·1	14·3	7·5
		Vietnam	49·5 45·8–54·0	52·4 48·8–56·9	55·8 52·7–59·2	59·6 55·4–64·2	63·0 58·5–67·7	66·3 62·1–70·6	52·2*	73·5	2·7	7·2
	Oceania		33·8 28·4–39·8	34·8 29·1–40·7	35·4 29·8–41·7	36·1 30·0–42·6	37·7 31·4–43·9	40·3 33·5–46·4	51·3	62·9	17·5	22·7
		American Samoa	54·6 51·8–57·5	55·6 52·4–58·6	60·2 57·7–62·8	61·7 59·0–64·2	63·1 60·5–65·7	63·3 59·8–66·7	74·0	77·4	19·4	14·1
		Federated States of Micronesia	41·1 33·4–50·2	43·6 35·9–51·9	46·8 38·3–54·9	49·8 40·9–57·6	51·5 42·0–59·2	53·8 44·5–61·5	62·5	73·3	21·5	19·5
		Fiji	43·9 40·1–47·8	44·1 40·6–47·7	44·7 41·7–47·7	45·0 41·9–48·1	45·4 42·0–48·9	46·6 42·5–50·6	69·2	76·1	25·3	29·6
		Guam	61·5 59·1–64·0	63·6 61·3–66·0	67·0 64·9–69·2	66·4 64·1–68·5	63·7 61·4–65·9	63·4 60·5–66·5	81·7	89·8	20·2	26·3
		Kiribati	35·4 31·8–39·3	36·5 33·0–40·0	39·7 36·3–43·4	41·1 37·0–44·6	43·6 39·0–47·7	44·9 40·0–49·7	53·3	61·7	17·9	16·8
		Marshall Islands	41·2 37·4–45·2	43·2 39·3–47·1	43·2 39·0–48·1	44·3 40·2–48·6	46·8 42·3–51·4	49·8 45·7–54·2	56·8	71·4	15·7	21·7
		Northern Mariana Islands	60·5 56·7–64·0	65·5 62·7–68·6	68·9 66·6–71·2	71·4 69·4–73·3	72·7 70·6–75·0	71·8 68·6–74·7	82·2	87·0	21·7	15·3
		Papua New Guinea	32·0 24·9–39·7	33·0 25·7–40·7	33·4 26·2–41·6	34·0 26·5–42·7	35·8 27·7–43·7	38·6 29·9–46·6	48·7	58·8	16·7	20·2
		Samoa	50·9 45·4–55·4	53·6 48·3–58·6	56·5 51·8–62·1	58·6 54·5–63·5	60·7 56·9–65·1	62·1 57·6–66·8	63·4	73·8	12·4	11·7
		Solomon Islands	33·4 25·5–41·6	35·7 27·6–44·3	38·3 30·3–46·6	38·0 30·0–46·6	40·1 31·7–48·8	43·1 34·5–51·8	48·5	60·2	15·1	17·1
		Tonga	55·0 50·9–58·6	56·3 53·0–59·4	57·3 54·3–60·2	58·4 55·2–61·2	60·1 56·7–63·4	62·1 58·0–65·6	63·8	73·2	8·8	11·1
		Vanuatu	36·4 28·3–44·3	37·4 28·5–46·3	38·3 30·0–47·4	38·4 30·4–46·1	41·1 32·7–49·0	43·1 34·9–50·2	52·8	66·4	16·3	23·2
Central Europe, eastern Europe, and central Asia			57·5 56·2–59·0	55·9 54·5–57·5	58·4 57·1–60·0	60·8 59·4–62·4	65·4 64·1–66·8	68·5 67·3–69·9	76·7	85·3	19·2	16·7
	Central Asia		50·7 49·2–52·5	49·2 47·6–51·0	50·7 48·9–52·6	52·4 50·8–54·3	56·6 55·0–58·4	59·9 58·4–61·6	72·3	78·2	21·6	18·3
		Armenia	56·8 54·8–59·1	55·6 53·5–58·0	59·0 56·9–61·5	60·0 57·9–62·6	63·7 61·7–66·2	67·5 65·5–70·0	71·7	80·6	14·9	13·1
		Azerbaijan	52·9 50·8–55·1	51·8 49·7–54·0	53·6 51·4–55·6	55·4 53·5–57·5	59·9 57·9–62·1	64·5 62·6–66·5	74·0	83·3	21·1	18·8
		Georgia	58·4 56·4–60·6	60·2 58·0–62·2	60·9 58·4–63·0	60·7 58·7–62·6	60·8 58·8–62·9	62·1 60·0–64·2	76·1	81·1	17·7	19·1
		Kazakhstan	55·1 53·5–56·9	49·9 48·2–51·9	51·3 49·6–53·3	51·9 50·1–54·0	57·9 56·3–59·8	61·1 59·2–63·3	75·5	84·7	20·4	23·6
		Kyrgyzstan	52·5 50·6–54·5	51·7 49·8–53·9	52·4 50·6–54·5	54·6 52·6–56·6	57·7 55·9–59·6	60·4 58·4–62·2	71·5	73·6	19·0	13·2
		Mongolia	42·3 39·8–45·0	42·1 39·7–44·9	44·7 42·3–47·4	50·9 47·6–53·5	54·9 51·1–57·4	58·5 54·8–61·2	64·4	76·8	22·1	18·3
		Tajikistan	48·7 46·8–51·2	48·1 45·8–50·3	48·6 46·4–51·2	51·7 49·7–54·1	55·4 53·4–57·8	58·6 56·7–60·7	63·8	70·0	15·1	11·4
		Turkmenistan	47·1 44·8–49·5	47·7 45·4–50·1	48·8 46·5–51·1	51·1 48·8–53·7	54·5 52·4–57·0	58·1 56·0–60·4	71·7	82·6	24·6	24·6
		Uzbekistan	51·9 49·9–54·1	51·7 49·5–53·9	54·0 51·9–56·2	56·1 53·9–58·4	59·2 57·1–61·6	62·3 60·2–64·5	67·1	76·5	15·3	14·2
	Central Europe		60·6 59·3–62·2	63·4 62·1–64·8	67·5 66·2–68·8	70·5 69·3–71·8	73·8 72·6–74·9	77·1 76·0–78·2	76·2	86·4	15·6	9·3
		Albania	62·4 60·6–64·2	65·1 63·3–67·0	68·5 66·7–70·4	71·6 69·7–73·4	75·3 73·1–77·2	78·2 76·0–80·2	69·7	79·1*	7·3	0·9
		Bosnia and Herzegovina	60·9 59·2–62·9	62·1 60·4–64·0	68·8 67·1–70·5	72·1 70·2–73·6	75·3 73·0–76·9	78·2 75·9–79·9	62·5*	79·3*	1·6	1·0
		Bulgaria	63·0 61·7–64·5	63·2 61·9–64·7	64·8 63·5–66·3	66·7 65·3–68·2	67·9 66·4–69·3	71·4 69·6–73·1	76·6	84·8	13·6	13·5
		Croatia	68·8 67·5–70·0	70·4 69·2–71·8	73·4 72·2–74·6	76·8 75·6–77·9	78·9 77·9–80·0	81·6 80·5–82·7	75·0	82·9	6·2	1·2
		Czech Republic	68·3 67·1–69·7	73·2 72·1–74·3	77·1 75·8–78·1	79·2 78·0–80·3	81·8 80·9–82·8	84·8 83·9–85·7	83·0	90·2	14·7	5·3
		Hungary	64·6 63·4–66·0	67·2 66·0–68·5	71·0 69·9–72·2	73·7 72·6–74·8	76·1 75·1–77·2	79·6 78·2–81·0	78·4	87·6	13·8	8·0
		Macedonia	61·9 60·3–63·9	63·4 61·9–65·3	67·0 65·7–68·5	69·9 68·4–71·3	74·2 72·7–75·5	76·0 73·8–78·0	74·1	81·1	12·2	5·1
		Montenegro	70·2 68·0–72·2	70·2 68·7–71·9	69·9 68·3–71·4	74·4 73·0–75·8	78·2 76·9–79·6	80·7 79·0–82·3	75·8	84·0	5·6	3·3
		Poland	62·1 60·8–63·6	65·4 64·2–66·8	70·8 69·5–72·0	73·8 72·4–75·0	76·3 75·1–77·5	79·6 78·2–81·0	76·8	88·8	14·7	9·2
		Romania	58·3 56·8–60·0	60·0 58·6–61·6	64·2 62·8–65·7	67·2 65·6–68·6	71·1 69·7–72·5	74·4 72·7–76·0	76·1	84·0	17·8	9·6
		Serbia	64·2 62·1–66·4	64·4 62·8–66·3	66·6 65·1–68·3	69·4 67·9–71·0	73·1 71·8–74·3	75·4 74·2–76·7	74·4	81·9	10·2	6·5
		Slovakia	66·6 65·0–68·2	69·4 67·8–70·9	70·8 69·2–72·2	72·2 70·7–73·6	74·9 73·6–76·2	78·6 77·3–79·9	77·3	88·5	10·7	9·9
		Slovenia	71·2 70·1–72·4	72·8 71·6–74·2	76·4 75·3–77·5	79·8 78·8–80·8	84·3 83·4–85·3	87·4 86·5–88·4	80·4	88·0*	9·2	0·6
	Eastern Europe		61·7 60·4–63·1	58·2 56·7–59·8	60·4 59·0–62·0	63·4 61·9–65·0	68·9 67·7–70·2	71·9 70·6–73·2	79·4	87·2	17·7	15·3
		Belarus	63·9 62·1–65·7	63·2 61·4–65·1	65·6 63·4–67·4	68·0 66·0–69·7	71·7 70·2–73·3	74·4 72·8–76·1	75·8	87·4	11·9	13·0
		Estonia	65·8 64·5–67·2	62·5 61·0–64·1	67·4 66·0–68·9	71·4 70·1–72·8	77·5 76·4–78·6	81·4 80·1–82·6	77·3	88·3	11·5	6·9
		Latvia	65·7 64·5–67·1	62·4 61·0–64·0	68·7 67·5–70·1	70·7 69·4–72·0	74·4 73·2–75·7	77·7 76·3–79·3	79·0	88·4	13·2	10·6
		Lithuania	67·0 65·8–68·3	64·2 62·8–65·6	69·6 68·5–71·0	70·2 69·0–71·6	73·6 72·5–74·9	76·6 75·5–77·9	77·6	87·0	10·6	10·4
		Moldova	59·6 58·1–61·4	56·0 54·3–57·9	60·8 59·0–62·5	64·8 62·9–66·7	66·8 65·1–68·4	73·1 71·2–74·9	72·8	76·7	13·2	3·6
		Russia	61·4 60·0–62·9	57·6 56·1–59·4	59·7 58·3–61·3	62·9 61·4–64·6	68·5 67·1–69·9	71·7 70·3–73·1	81·1	88·1	19·8	16·4
		Ukraine	62·8 61·3–64·5	59·4 57·8–61·2	62·0 60·6–63·8	64·4 62·9–66·1	70·5 69·2–72·0	72·7 70·8–74·5	76·7	85·0	13·9	12·4
High-income			71·1 70·2–72·2	74·6 73·6–75·6	77·2 76·3–78·2	79·6 78·8–80·5	81·6 80·8–82·4	83·1 82·3–83·8	83·3	90·0	12·2	7·0
	High-income Asia Pacific		72·6 71·3–73·8	77·2 76·2–78·3	79·8 78·8–80·8	83·3 82·4–84·2	86·0 85·2–86·8	87·4 86·7–88·2	82·6	90·0	10·0	2·6
		Brunei	62·0 60·1–64·0	64·3 62·4–66·2	66·7 65·1–68·6	68·6 67·0–70·3	69·5 68·0–71·2	70·0 68·3–71·8	83·0	91·3	21·0	21·3
		Japan	78·3 77·4–79·2	80·7 79·8–81·6	82·9 82·1–83·7	85·6 84·9–86·3	87·8 87·2–88·6	89·0 88·3–89·8	84·5	90·4	6·2	1·3
		South Korea	61·7 59·6–63·9	70·5 69·0–72·2	74·6 73·4–76·0	80·4 79·3–81·6	83·9 82·9–84·9	85·8 84·7–87·0	77·3	89·0	15·6	3·2
		Singapore	67·4 66·2–68·8	72·8 71·6–74·1	77·3 76·2–78·5	80·8 79·8–81·9	84·2 83·3–85·1	86·3 85·3–87·3	77·3	89·6	9·9	3·3
	Australasia		77·2 76·2–78·2	80·1 79·2–81·1	82·9 82·1–83·8	85·3 84·6–86·1	87·4 86·7–88·1	89·1 88·4–89·8	84·5	90·8	7·3	1·7
		Australia	78·0 77·0–79·1	80·8 79·8–81·8	83·7 82·9–84·6	86·2 85·4–86·9	88·2 87·5–88·9	89·8 89·1–90·6	84·7	91·0	6·7	1·1
		New Zealand	74·2 73·2–75·2	77·6 76·6–78·6	80·0 79·2–80·9	82·5 81·7–83·4	84·2 83·4–85·0	86·2 85·4–87·2	83·4	89·9	9·2	3·6
	Western Europe		73·2 72·3–74·2	76·8 76·0–77·8	79·9 79·0–80·8	82·9 82·1–83·7	84·9 84·2–85·6	86·8 86·0–87·5	80·9	88·8	7·7	2·1
		Andorra	84·7 82·7–87·9	88·0 86·0–92·2	91·1 89·4–93·5	92·6 91·1–94·4	94·0 92·5–95·5	94·6 93·3–96·0	85·8*	91·2*	1·1	..
		Austria	74·4 73·2–75·5	78·2 77·1–79·2	81·6 80·7–82·5	84·2 83·4–85·0	86·1 85·3–86·9	88·2 87·3–89·0	83·2	90·0	8·8	1·8
		Belgium	75·4 74·3–76·6	78·8 77·9–79·8	81·6 80·6–82·6	84·6 83·6–85·4	86·3 85·4–87·1	87·9 86·8–88·8	82·6	89·6	7·2	1·8
		Cyprus	72·0 70·6–73·5	73·4 72·2–74·7	76·4 75·0–77·5	79·2 78·1–80·3	82·6 81·6–83·7	85·3 84·2–86·4	78·2	89·5	6·1	4·2
		Denmark	76·6 75·5–77·7	77·2 76·1–78·2	79·5 78·4–80·5	81·9 81·0–82·8	83·6 82·7–84·6	85·7 84·7–86·7	85·6	90·9	9·0	5·2
		Finland	75·4 74·3–76·6	78·8 77·8–79·9	82·0 81·0–82·9	84·5 83·5–85·3	87·2 86·4–88·1	89·6 88·6–90·5	83·0	90·2*	7·6	0·6
		France	74·3 73·2–75·4	77·5 76·5–78·5	80·3 79·4–81·3	84·2 83·4–85·1	85·7 84·9–86·5	87·9 86·9–88·9	79·0	86·7*	4·8	..
		Germany	73·1 72·0–74·3	77·1 76·1–78·2	80·6 79·4–81·6	83·2 82·0–84·1	84·9 83·9–85·7	86·4 85·4–87·3	83·5	90·6	10·4	4·3
		Greece	76·5 75·6–77·6	79·5 78·7–80·5	82·2 81·4–83·0	84·4 83·6–85·2	85·2 84·4–86·0	87·0 86·1–87·9	77·0*	85·9*	0·5	..
		Iceland	81·9 81·0–83·0	84·2 83·3–85·2	87·1 86·1–88·0	89·8 88·9–90·6	92·1 91·2–92·8	93·6 92·9–94·4	83·1	90·3*	1·2	..
		Ireland	75·6 74·5–76·7	78·4 77·3–79·5	79·6 78·5–80·7	83·5 82·6–84·4	86·0 85·2–86·9	88·4 87·5–89·3	79·0	90·0	3·3	1·6
		Israel	71·3 70·2–72·5	74·3 73·2–75·3	77·5 76·2–78·6	80·3 79·0–81·4	83·7 82·5–84·6	85·5 84·2–86·5	80·3	87·1	9·0	1·6
		Italy	76·2 75·3–77·2	79·1 78·2–80·0	82·5 81·7–83·4	85·5 84·7–86·3	87·5 86·8–88·2	88·7 87·8–89·6	81·8	88·1*	5·5	..
		Luxembourg	74·5 73·4–75·7	78·8 77·9–79·9	82·2 81·3–83·1	84·9 84·1–85·8	87·5 86·6–88·3	89·3 88·4–90·2	83·6	90·9	9·1	1·6
		Malta	74·7 73·5–76·0	76·8 75·6–78·0	78·9 77·8–80·1	81·4 80·3–82·6	82·9 81·8–83·9	85·1 84·0–86·1	74·9*	84·5*	0·1	..
		Netherlands	79·2 78·2–80·2	80·8 79·7–81·8	82·1 81·0–83·1	85·1 84·1–86·0	88·2 87·4–88·9	89·5 88·6–90·4	83·2	90·3*	4·0	0·8
		Norway	77·5 76·5–78·6	81·0 80·1–82·0	82·8 81·8–83·7	85·9 85·1–86·8	88·0 87·2–88·9	90·5 89·6–91·4	85·9	91·6	8·4	1·1
		Portugal	67·2 66·1–68·6	71·5 70·4–72·7	74·7 73·7–75·8	79·7 78·7–80·7	81·9 81·1–82·9	84·5 83·6–85·5	71·4	80·5*	4·1	..
		Spain	73·9 73·0–75·0	78·1 77·3–79·0	81·4 80·6–82·3	84·1 83·4–84·9	87·2 86·5–87·8	89·6 88·8–90·3	74·7*	85·7*	0·8	..
		Sweden	80·4 79·5–81·4	83·9 83·0–84·8	85·7 84·9–86·6	87·0 86·2–87·9	88·7 87·9–89·7	90·5 89·6–91·4	82·9	90·2*	2·4	..
		Switzerland	81·4 80·5–82·3	83·4 82·6–84·3	85·4 84·7–86·2	88·2 87·5–88·9	90·1 89·5–90·8	91·8 90·9–92·6	86·4	91·4*	5·0	..
		UK	74·3 73·2–75·3	76·6 75·7–77·6	78·4 77·5–79·3	80·6 79·8–81·4	82·7 82·0–83·5	84·6 83·8–85·4	83·4	90·3	9·2	5·7
	Southern Latin America		57·6 56·3–59·3	61·1 59·8–62·6	64·7 63·3–66·1	66·7 65·4–68·1	68·1 66·9–69·4	70·0 68·8–71·3	73·4	82·6	15·7	12·5
		Argentina	57·4 56·0–59·1	60·3 59·0–61·9	63·5 62·1–65·0	65·3 64·0–66·8	66·6 65·3–67·9	68·4 67·1–69·7	73·3	81·9	15·9	13·5
		Chile	58·8 57·4–60·4	64·2 62·7–65·6	69·2 67·7–70·5	72·0 70·5–73·3	73·9 72·6–75·1	76·0 74·5–77·4	73·8	84·5	15·0	8·5
		Uruguay	60·8 59·4–62·2	63·1 61·9–64·6	67·1 65·7–68·4	68·7 67·4–70·0	70·6 69·2–71·9	72·0 70·5–73·5	72·5	79·7	11·7	7·7
	High-income North America		74·0 73·0–75·1	76·3 75·3–77·3	78·1 77·3–79·0	79·2 78·3–80·1	80·9 80·1–81·7	81·8 81·0–82·7	88·5	91·5	14·4	9·7
		Canada	78·9 78·0–79·9	80·7 79·8–81·6	83·2 82·2–84·0	84·7 83·9–85·5	86·3 85·5–87·1	87·6 86·8–88·5	88·1	91·6	9·2	4·0
		Greenland	59·0 56·5–62·0	62·3 59·8–65·2	64·3 62·0–67·5	65·8 63·5–68·9	68·1 66·0–71·3	71·0 68·7–74·1	72·5	81·0	13·6	10·0
		USA	73·7 72·7–74·8	76·0 75·0–77·0	77·8 76·9–78·7	78·8 77·9–79·7	80·5 79·6–81·3	81·3 80·5–82·2	88·5	91·5	14·8	10·2
Latin America and Caribbean			46·5 44·9–48·5	50·8 49·2–52·6	54·6 53·0–56·4	57·4 55·8–59·1	59·6 58·0–61·3	61·9 60·4–63·5	65·4	75·3	18·9	13·4
	Caribbean		42·4 40·2–44·9	45·9 43·9–48·1	48·8 46·6–51·0	50·8 48·6–53·3	52·3 49·9–54·7	54·5 51·9–57·2	68·5	74·9	26·1	20·4
		Antigua and Barbuda	56·6 54·6–58·7	57·8 55·9–59·9	60·3 58·4–62·5	62·5 60·5–64·6	64·9 63·0–66·9	66·7 64·6–68·9	79·2	87·0	22·6	20·3
		The Bahamas	50·9 48·2–53·6	54·1 51·7–56·6	57·0 54·6–59·4	59·9 57·5–62·2	62·1 59·7–64·5	63·9 61·0–66·7	77·9	86·6	27·0	22·7
		Barbados	55·9 53·7–58·4	59·5 57·5–61·9	62·6 60·5–64·8	64·3 62·4–66·1	65·5 63·6–67·5	66·8 64·4–69·3	75·6	82·8	19·7	16·0
		Belize	49·7 47·3–52·2	50·5 48·2–53·1	50·6 48·1–53·2	53·7 51·3–56·4	56·3 53·9–58·7	58·3 55·6–61·3	61·4	74·8	11·7	16·5
		Bermuda	60·8 58·9–62·8	64·8 62·9–66·6	71·6 69·7–73·5	74·1 72·4–75·8	77·0 75·2–78·6	79·0 77·0–81·0	86·9	91·1	26·0	12·1
		Cuba	64·1 62·7–65·7	65·2 63·9–66·8	67·7 66·4–69·0	70·5 69·3–71·8	72·1 71·0–73·4	73·5 72·3–74·9	74·8	81·5	10·7	7·9
		Dominica	53·0 50·7–55·4	54·6 52·3–57·0	57·7 55·5–59·9	59·0 56·8–61·3	58·5 56·1–60·8	58·1 55·2–61·0	72·0	80·4	19·0	22·3
		Dominican Republic	47·9 45·8–50·2	52·2 50·1–54·4	56·6 54·4–58·7	58·6 56·4–60·5	60·8 58·5–62·6	62·5 60·2–64·5	64·1	75·6	16·2	13·1
		Grenada	49·4 46·3–52·5	52·8 50·3–55·4	54·0 51·5–56·5	55·1 52·5–57·5	57·0 54·5–59·6	58·3 55·2–61·2	67·3	80·5	17·9	22·2
		Guyana	39·3 36·9–42·0	41·3 38·9–44·1	42·5 39·9–45·1	44·7 42·2–47·8	47·0 44·2–50·0	49·8 46·7–53·2	62·7	74·4	23·4	24·6
		Haiti	24·9 21·2–28·9	29·2 25·7–33·1	32·7 29·0–36·8	34·8 30·4–39·0	35·9 31·5–40·3	38·5 33·7–43·5	48·2	54·6	23·3	16·1
		Jamaica	55·0 52·6–57·4	58·0 55·5–60·5	59·5 56·8–61·9	62·2 59·1–64·8	62·9 59·7–65·5	63·7 60·1–66·5	71·2	77·7	16·2	14·1
		Puerto Rico	64·5 62·9–66·2	66·1 64·7–67·6	69·8 68·6–71·3	72·6 71·3–73·9	74·0 72·9–75·3	76·6 75·1–78·1	81·7	89·6	17·2	13·0
		Saint Lucia	49·7 47·1–52·5	53·0 50·8–55·4	56·0 53·9–58·2	58·5 56·2–60·7	61·1 58·7–63·5	62·5 59·3–65·4	69·9	79·4	20·2	16·9
		Saint Vincent and the Grenadines	50·4 47·9–53·1	51·4 49·0–53·7	53·0 50·8–55·3	55·2 53·2–57·6	56·7 54·5–59·0	57·5 54·7–60·2	71·0	80·0	20·5	22·5
		Suriname	46·7 44·4–49·0	48·6 46·2–51·0	49·5 47·2–52·1	51·0 48·4–53·5	53·5 51·0–56·0	56·7 53·9–59·5	67·5	76·7	20·8	20·0
		Trinidad and Tobago	51·6 49·6–53·7	53·5 51·5–55·6	55·8 53·8–57·8	58·6 56·7–60·7	60·5 58·4–62·6	62·1 59·4–64·6	75·5	86·4	23·9	24·4
		Virgin Islands, USA	60·4 58·5–62·6	63·1 61·3–65·1	65·8 63·9–67·7	68·8 66·9–70·6	69·8 67·7–71·7	70·2 67·9–72·4	81·2	89·9	20·8	19·7
	Andean Latin America		43·2 41·2–45·6	47·0 45·1–49·3	53·5 51·5–55·5	58·1 56·1–60·1	60·9 59·1–62·9	64·1 62·1–65·9	64·9	75·6	21·7	11·5
		Bolivia	40·5 37·6–43·6	43·8 41·2–46·8	48·5 46·2–51·2	53·3 50·7–55·8	56·4 53·5–59·2	59·2 55·9–62·7	56·6	72·6	16·1	13·4
		Ecuador	45·4 43·5–47·7	48·7 46·8–50·9	53·5 51·3–55·6	56·1 53·9–58·3	58·6 56·3–60·7	61·2 58·8–63·6	66·2	75·7	20·8	14·5
		Peru	45·9 43·6–48·4	49·8 47·5–52·2	57·1 54·8–59·3	62·7 60·5–64·7	65·9 63·7–67·9	69·6 67·0–71·8	66·7	76·8	20·8	7·2
	Central Latin America		47·6 45·9–49·6	52·2 50·5–54·1	55·8 54·1–57·5	58·3 56·5–59·9	60·2 58·5–61·9	62·4 60·8–64·1	66·6	76·1	19·0	13·7
		Colombia	51·1 49·5–53·2	54·9 53·3–56·6	58·4 56·8–60·1	61·6 59·9–63·2	64·4 62·5–66·2	67·8 65·8–69·7	67·1	76·5	16·0	8·6
		Costa Rica	62·1 60·4–63·6	62·8 61·1–64·4	65·4 63·8–67·1	68·6 67·0–70·1	70·8 69·1–72·3	72·9 70·9–74·6	68·6	78·0	6·5	5·1
		El Salvador	44·3 41·9–46·8	49·0 46·8–51·3	55·3 53·1–57·6	59·6 56·8–61·8	62·2 58·8–64·6	64·4 61·2–66·9	57·2	73·0	12·9	8·6
		Guatemala	40·1 37·9–42·5	44·4 42·3–46·8	48·7 46·6–51·0	51·3 48·9–53·5	53·2 50·8–55·5	55·7 52·7–58·8	50·7	66·9	10·6	11·2
		Honduras	42·3 40·0–44·8	45·7 42·9–48·6	47·6 43·8–51·2	49·5 45·3–53·4	51·8 47·7–56·0	53·9 49·9–57·8	51·5	69·5	9·1	15·6
		Mexico	49·2 47·6–51·2	54·1 52·4–55·9	57·4 55·6–59·1	59·1 57·4–60·7	60·5 58·9–62·1	62·6 61·0–64·2	68·9	77·6	19·7	15·1
		Nicaragua	49·7 47·5–52·1	51·7 49·7–53·8	55·5 53·4–57·6	58·8 56·5–60·9	61·8 59·3–63·9	64·3 61·8–66·7	53·1	68·9	3·4	4·7
		Panama	52·9 50·7–55·2	56·7 54·6–58·7	59·8 57·6–61·8	61·3 58·9–63·5	62·2 59·7–64·3	64·4 61·4–67·0	71·6	79·9	18·7	15·5
		Venezuela	53·1 51·4–54·9	55·6 53·8–57·5	59·3 57·5–61·0	62·3 60·7–64·0	64·2 62·5–65·8	64·7 62·2–66·9	71·4	78·5	18·3	13·8
	Tropical Latin America		50·1 48·3–52·0	53·7 52·0–55·6	56·9 55·3–58·7	59·8 58·3–61·4	62·5 61·0–64·1	64·7 63·2–66·5	63·7	74·7	13·7	10·0
		Brazil	50·1 48·3–52·0	53·8 52·0–55·6	57·0 55·4–58·7	59·9 58·4–61·6	62·6 61·2–64·3	64·9 63·4–66·7	63·8	74·7	13·8	9·8
		Paraguay	53·0 50·7–55·4	55·1 52·9–57·5	56·6 54·0–58·9	57·4 54·8–59·9	58·6 56·1–61·0	60·4 57·4–63·2	61·7	74·0	8·7	13·6
North Africa and Middle East			43·8 41·9–46·0	46·5 44·5–48·6	49·9 48·0–51·9	52·8 51·0–54·8	55·7 53·9–57·7	58·4 56·5–60·5	55·7	72·3	11·9	13·8
		Afghanistan	24·7 18·6–32·0	24·5 18·5–31·6	24·8 18·7–31·7	27·1 21·2–33·5	29·4 23·5–35·7	32·5 26·6–38·6	38·0	47·6	13·3	15·1
		Algeria	48·2 45·5–51·1	52·6 49·9–55·2	56·2 53·7–58·7	59·6 57·3–62·1	62·2 59·9–64·3	63·7 61·3–66·3	58·3	71·4	10·1	7·6
		Bahrain	59·7 57·4–62·2	63·2 60·6–65·6	67·3 64·9–69·7	71·3 69·0–73·4	77·2 75·1–79·1	79·0 76·2–81·7	71·8	82·3	12·1	3·3
		Egypt	45·0 42·5–47·5	49·3 47·1–51·7	54·4 52·4–56·4	56·9 54·8–58·7	58·2 56·2–60·1	61·0 58·7–63·1	58·0	73·0	12·9	12·0
		Iran	54·6 51·5–57·6	59·6 56·5–62·3	63·5 60·9–65·6	65·7 62·6–68·6	68·9 65·4–72·3	71·1 67·9–74·2	60·0	77·5	5·4	6·4
		Iraq	51·1 47·5–54·4	50·7 47·4–54·0	51·8 48·4–55·0	54·4 50·4–58·0	57·2 53·2–61·3	60·1 55·8–64·3	53·2*	70·1	2·1	10·0
		Jordan	59·1 56·2–61·9	62·0 59·2–64·8	65·0 62·6–67·3	68·4 66·4–70·3	74·3 72·8–75·8	76·5 74·4–78·4	63·1	76·3*	4·0	..
		Kuwait	71·7 70·1–73·3	71·4 70·0–72·8	74·9 73·6–76·1	75·7 74·5–77·0	77·7 76·4–78·9	82·0 79·9–84·0	76·0	88·5	4·3	6·4
		Lebanon	58·7 55·3–62·2	63·4 60·1–66·5	68·5 65·2–71·7	73·1 69·7–76·4	77·0 73·4–80·9	80·0 76·0–84·3	69·5	80·5*	10·9	0·6
		Libya	60·0 57·3–62·8	63·7 61·2–66·4	65·4 63·1–67·8	67·1 64·9–69·3	69·7 67·4–72·0	69·9 67·2–72·6	61·5*	74·0	1·4	4·1
		Morocco	44·0 40·7–47·3	47·8 44·7–51·1	52·6 49·2–56·0	55·6 51·4–59·1	58·7 54·1–62·8	61·3 56·6–66·0	49·4	63·0*	5·4	1·7
		Palestine	61·8 57·8–65·5	65·6 62·3–68·6	68·3 66·2–70·4	68·7 66·6–70·5	69·0 66·3–71·5	70·5 67·2–74·1	55·8*	69·4*	..	..
		Oman	66·1 62·0–70·4	71·4 67·6–74·9	74·4 71·3–77·0	76·2 74·2–78·0	72·9 71·0–75·1	77·1 74·6–80·1	54·3*	78·6*	..	1·5
		Qatar	70·8 68·1–73·3	71·3 68·8–73·8	73·1 70·9–75·6	77·5 75·0–79·7	83·1 80·7–85·3	85·2 82·0–88·3	72·9*	84·5*	2·1	..
		Saudi Arabia	63·4 61·1–65·8	66·8 64·8–68·8	71·2 69·6–72·7	74·2 72·8–75·6	77·0 75·6–78·4	79·4 77·7–81·1	65·4*	81·0*	2·0	1·6
		Sudan	36·6 32·4–41·0	38·8 34·1–43·2	42·0 37·2–46·7	44·9 40·1–49·6	47·4 42·6–52·5	50·1 45·0–55·1	46·6	56·4	10·0	6·4
		Syria	58·2 54·9–61·2	63·1 59·5–66·2	68·2 65·3–70·7	71·4 69·0–73·4	73·8 71·9–75·5	74·6 72·1–77·0	52·1*	70·4*	..	..
		Tunisia	53·0 50·3–55·7	56·9 54·2–59·4	61·4 59·0–64·0	64·5 61·6–67·4	67·4 64·3–70·7	70·1 66·6–73·8	59·1	74·3	6·1	4·3
		Turkey	51·3 48·8–53·9	55·4 53·0–57·8	62·4 60·3–64·4	68·6 66·7–70·6	74·3 72·4–75·9	76·2 74·3–78·1	65·2	75·9*	13·9	..
		United Arab Emirates	56·9 52·7–61·0	60·8 57·3–64·3	64·7 61·9–67·8	69·0 66·7–71·3	71·4 68·1–74·6	72·2 68·0–76·3	73·7	89·2	16·8	17·1
		Yemen	35·2 27·7–43·5	38·0 30·3–46·6	41·3 32·8–50·0	44·5 36·1–53·3	47·8 38·8–56·0	49·6 40·4–57·6	37·0*	54·1*	1·8	4·5
South Asia			30·7 28·5–33·3	32·9 30·8–35·2	35·1 32·9–37·6	38·1 36·0–40·5	41·1 39·0–43·4	44·4 42·3–46·7	48·5	66·3	17·7	21·9
		Bangladesh	32·6 29·5–35·7	35·8 32·8–38·8	39·6 36·8–42·4	44·3 41·7–47·2	48·7 46·1–51·4	51·7 48·4–54·9	47·0	61·1	14·4	9·4
		Bhutan	34·6 30·4–38·8	37·6 33·9–41·7	42·3 38·6–45·8	46·2 42·4–49·8	50·1 46·4–53·9	52·7 48·5–56·7	47·3	65·9	12·7	13·2
		India	30·7 28·4–33·5	33·1 30·8–35·6	35·3 32·9–37·9	38·2 36·0–40·7	41·2 38·9–43·6	44·8 42·6–47·2	48·8	68·4	18·0	23·6
		Nepal	34·0 30·2–38·1	37·1 33·8–40·5	41·6 38·6–44·6	45·7 42·3–49·1	48·2 44·2–52·1	50·8 46·7–55·0	44·4	55·7	10·4	4·9
		Pakistan	36·5 33·3–40·3	35·6 32·2–39·3	36·3 32·9–40·1	38·1 34·9–41·9	41·2 37·5–45·1	43·1 39·2–47·1	47·2	60·7	10·6	17·7
Sub-Saharan Africa			32·3 29·7–35·2	33·5 31·2–36·1	34·6 32·4–36·9	37·4 35·3–39·7	40·5 38·4–42·9	42·2 39·6–44·9	46·6	52·3	14·3	10·1
	Central sub-Saharan Africa		31·1 25·9–36·8	32·1 27·0–37·4	33·0 27·8–37·8	35·7 30·0–41·0	37·2 30·9–42·7	38·6 31·8–45·6	45·7	48·1	14·6	9·5
		Angola	25·8 12·2–43·4	28·1 12·9–45·2	31·2 15·3–47·8	35·6 18·3–51·1	37·7 18·7–52·1	40·7 20·3–54·9	46·1	55·3	20·3	14·6
		Central African Republic	25·5 20·3–30·6	25·8 19·1–33·9	26·7 18·2–37·7	28·0 18·3–39·8	28·8 18·6–40·9	28·6 17·4–41·3	44·0	47·3	18·6	18·7
		Congo (Brazzaville)	32·2 26·9–38·0	31·4 26·8–36·4	33·3 28·7–38·4	39·3 34·4–44·5	41·2 34·9–47·1	43·5 34·2–52·7	53·6	65·5	21·4	22·0
		DR Congo	35·6 29·2–42·6	36·1 30·7–41·5	36·2 31·1–41·0	38·1 33·0–43·0	39·3 33·8–44·8	40·4 33·1–49·0	44·7	45·2*	9·1	4·8
		Equatorial Guinea	26·1 12·0–45·2	27·5 12·5–46·7	35·4 17·5–50·6	42·9 23·0–55·3	45·6 25·6–57·1	48·4 27·9–59·4	46·4	72·5	20·4	24·1
		Gabon	39·1 34·9–43·4	40·1 36·1–44·4	41·8 37·1–46·3	44·1 39·1–48·9	48·4 42·3–54·2	51·4 42·7–59·0	61·1	74·0	22·0	22·6
	Eastern sub-Saharan Africa		29·6 27·1–32·7	31·2 28·6–34·1	33·8 31·3–36·6	37·5 35·0–40·1	40·5 37·4–43·6	42·4 38·6–46·2	43·1	49·9	13·5	7·5
		Burundi	23·5 17·0–31·9	23·4 18·3–29·1	27·0 22·3–31·7	35·5 30·5–40·3	40·5 34·2–47·3	40·4 31·6–48·9	39·9	45·3*	16·4	4·9
		Comoros	32·7 23·7–41·6	34·3 27·9–40·1	38·4 33·8–42·9	44·1 39·2–48·8	47·2 41·6–52·5	47·7 39·6–55·2	46·0	50·5*	13·3	2·8
		Djibouti	38·9 30·8–47·1	38·8 29·0–48·0	39·4 28·6–50·4	40·9 29·7–51·4	43·2 31·3–54·1	44·7 33·1–54·8	48·9	60·2	10·0	15·4
		Eritrea	28·9 24·4–33·9	35·3 29·8–41·2	38·0 29·2–47·1	38·8 27·6–49·5	37·8 26·4–48·5	38·1 25·6–49·9	41·5	48·9*	12·7	10·9
		Ethiopia	23·1 19·1–28·2	26·8 22·8–31·5	30·6 26·3–35·8	34·9 30·9–39·6	40·4 34·3–47·0	44·2 35·2–52·6	36·8	48·1*	13·7	3·9
		Kenya	42·6 39·3–45·6	42·3 39·1–45·7	44·0 40·7–47·4	46·4 43·1–49·6	47·5 44·3–50·6	48·7 45·2–52·2	49·5	61·1	6·8	12·4
		Madagascar	34·8 31·0–38·7	36·5 32·6–43·3	38·7 34·1–47·3	41·6 36·5–49·4	42·5 35·5–50·3	43·7 34·9–53·1	46·4	50·8*	11·6	7·1
		Malawi	34·7 29·9–39·6	35·4 28·8–42·4	36·5 29·1–43·1	40·6 34·1–46·8	44·3 37·8–50·5	47·0 38·4–55·1	42·4	48·4*	7·7	1·4
		Mozambique	33·2 29·0–37·5	35·1 30·9–39·3	36·4 31·4–41·8	39·6 33·4–46·2	40·9 33·9–48·9	43·0 33·7–53·2	31·5*	47·1*	..	4·1
		Rwanda	29·9 25·4–34·4	23·3 18·5–27·7	30·4 25·4–35·0	42·6 37·4–47·9	47·0 40·4–53·6	47·8 39·0–55·8	43·3	51·0*	13·4	3·2
		Somalia	29·1 13·9–45·8	29·3 14·8–46·3	30·1 14·9–47·3	31·8 15·9–49·6	33·3 16·0–50·0	34·2 17·2–50·8	35·5*	38·6*	6·4	4·4
		South Sudan	33·4 17·2–47·6	34·7 18·0–49·6	37·5 19·8–52·3	39·0 20·5–53·5	38·8 17·9–53·6	38·8 18·8–53·2	38·0*	46·4*	4·6	7·6
		Tanzania	39·9 36·0–44·1	41·0 36·7–45·6	43·1 38·0–48·3	46·7 40·2–52·3	48·8 39·9–56·5	49·9 39·0–59·0	47·0	54·6*	7·2	4·6
		Uganda	34·0 28·8–40·9	33·9 28·1–41·4	35·2 30·2–41·2	38·2 33·6–43·3	41·4 34·9–48·6	42·9 33·6–53·7	43·2	51·3*	9·2	8·3
		Zambia	37·4 32·6–42·2	34·6 29·6–39·9	34·3 29·4–39·0	35·5 31·1–40·2	37·4 32·2–42·6	41·6 33·9–50·1	49·0	60·7	11·6	19·2
	Southern sub-Saharan Africa		44·8 42·2–47·3	46·7 44·0–49·2	43·4 40·6–46·2	43·1 40·2–46·1	46·3 43·5–49·1	49·2 46·6–51·9	65·3	74·8	20·5	25·6
		Botswana	44·9 27·6–58·1	45·4 24·3–59·3	43·7 20·5–59·4	43·9 21·8–60·3	48·6 26·0–62·5	51·1 28·0–63·6	55·4*	73·9	10·5	22·9
		Lesotho	40·8 35·3–46·9	41·8 36·4–48·4	39·4 33·0–45·2	33·2 27·9–38·4	34·4 27·4–41·6	35·7 26·1–45·9	49·1	65·1	8·4	29·3
		Namibia	41·8 38·1–45·6	41·9 37·9–45·7	39·9 34·6–45·2	43·5 37·3–49·2	50·4 43·8–57·3	53·7 44·7–61·5	58·0	72·9	16·2	19·2
		South Africa	45·6 42·7–48·4	47·9 45·4–50·4	44·8 41·8–48·1	45·2 41·6–48·7	49·4 46·0–52·8	52·0 49·2–54·9	69·7	77·1	24·1	25·1
		Swaziland	41·5 35·7–47·6	45·7 38·7–54·2	40·7 33·3–47·5	35·1 27·8–42·1	37·8 28·9–47·8	41·9 30·7–54·5	55·0	73·3	13·5	31·4
		Zimbabwe	48·1 43·8–52·9	49·5 41·1–57·0	45·4 36·8–52·2	41·8 34·4–48·6	42·1 35·8–48·2	48·7 40·1–57·3	56·0	66·6	7·9	17·9
	Western sub-Saharan Africa		35·3 31·3–39·3	36·2 33·1–39·4	37·0 34·3–40·2	39·7 36·9–42·7	43·3 40·2–46·3	44·8 40·9–48·1	46·2	53·3	10·9	8·5
		Benin	36·9 32·9–41·2	37·0 32·8–41·3	37·3 32·7–42·2	40·4 34·5–46·5	41·5 33·0–49·9	43·0 32·8–52·9	42·8	49·7*	5·9	6·7
		Burkina Faso	32·9 28·9–37·4	34·1 29·8–38·7	36·0 31·6–40·7	40·3 35·4–45·2	42·7 36·2–49·4	42·9 33·8–51·5	33·1*	45·2*	0·2	2·3
		Cameroon	38·3 34·6–42·2	37·7 33·5–41·8	37·2 32·3–42·6	41·0 36·1–46·4	42·5 35·6–49·1	44·4 35·0–53·3	48·9	60·4	10·6	16·0
		Cape Verde	50·1 47·4–52·6	49·3 45·3–53·2	50·8 45·5–56·2	53·7 49·2–58·4	57·9 55·6–60·3	61·7 58·1–64·9	48·3*	67·6	..	5·8
		Chad	35·6 30·8–40·6	35·2 30·8–40·3	32·1 27·1–37·4	34·1 26·9–41·1	36·3 26·8–46·1	37·7 27·1–48·2	38·1*	47·5*	2·5	9·8
		Côte d'Ivoire	35·5 31·4–39·4	33·2 28·5–38·3	34·4 29·2–39·7	37·6 32·4–42·5	40·7 34·2–47·2	42·4 33·7–50·8	46·3	51·5	10·8	9·2
		The Gambia	41·3 32·1–50·4	42·4 35·2–49·7	43·3 38·6–48·2	45·6 41·6–50·1	47·7 43·2–52·5	49·7 43·1–56·3	45·2*	49·0*	4·0	..
		Ghana	34·8 28·3–40·9	38·5 33·9–43·3	40·3 35·4–45·5	44·2 38·5–50·4	47·3 38·8–55·7	49·7 40·0–58·8	49·8	64·2	15·0	14·6
		Guinea	32·6 28·6–36·9	33·6 29·6–37·9	34·0 30·1–38·3	37·0 32·6–41·5	37·6 32·6–43·0	38·6 30·7–46·6	40·4	47·1	7·8	8·5
		Guinea-Bissau	32·7 15·3–46·7	33·1 14·7–47·3	33·6 15·7–48·2	33·3 14·9–48·3	35·1 16·2–49·1	36·3 15·0–50·2	40·8*	47·8*	8·1	11·5
		Liberia	34·7 28·9–40·5	37·1 32·3–41·9	39·5 34·7–44·7	41·7 37·0–46·7	43·2 38·2–48·5	45·4 37·8–52·9	43·9	47·3*	9·2	1·9
		Mali	32·7 28·8–37·0	33·8 29·9–37·9	37·7 33·7–42·0	43·5 39·2–47·8	44·4 39·2–49·9	45·6 38·1–53·2	35·1*	44·8*	2·4	..
		Mauritania	37·3 33·3–41·4	38·9 34·8–43·5	42·9 38·5–47·8	46·9 42·1–52·7	49·6 43·5–55·4	52·0 43·8–60·3	46·6	53·4*	9·2	1·4
		Niger	31·8 26·9–36·9	33·1 28·6–37·9	34·6 30·3–38·9	37·7 33·2–42·3	40·3 34·7–45·5	41·0 32·3–48·9	32·6*	38·2*	0·8	..
		Nigeria	38·3 31·2–45·4	39·7 34·4–45·0	40·6 36·2–45·4	43·1 38·4–47·9	48·8 43·2–54·4	51·3 43·2–57·0	48·2	61·4	9·9	10·1
		São Tomé and Príncipe	41·3 37·8–45·2	41·9 38·0–45·7	42·8 39·3–46·8	44·0 39·3–48·4	47·3 40·9–53·7	49·6 40·7–58·6	48·1	58·8	6·8	9·2
		Senegal	37·6 33·3–41·8	38·1 34·0–42·2	38·9 34·8–43·0	41·5 35·7–47·1	42·9 34·4–51·2	44·4 34·0–54·3	43·5	49·4*	6·0	4·9
		Sierra Leone	37·6 30·8–45·1	37·2 32·1–42·8	35·4 30·7–40·4	36·1 31·8–40·8	38·2 33·1–43·7	41·3 33·3–49·1	41·3*	48·9*	3·6	7·6
		Togo	37·4 33·0–41·8	36·9 32·8–41·3	36·9 32·1–42·6	40·1 34·8–45·6	41·8 36·1–47·9	44·3 36·6–52·5	45·5	50·3*	8·2	6·0

Geographies that exceed the HAQ Index frontier associated with their level of SDI have double dots in place of values in the columns representing the difference between observed and frontier HAQ Index levels. GBD=Global Burden of Disease. HAQ Index=Healthcare Access and Quality Index. SDI=Socio-demographic Index. UI=Uncertainty interval.

* Geographies for which the HAQ Index frontier in 1990 or 2015 is within the 95% UIs of their observed HAQ Index values for those years.

**Figure 5:**
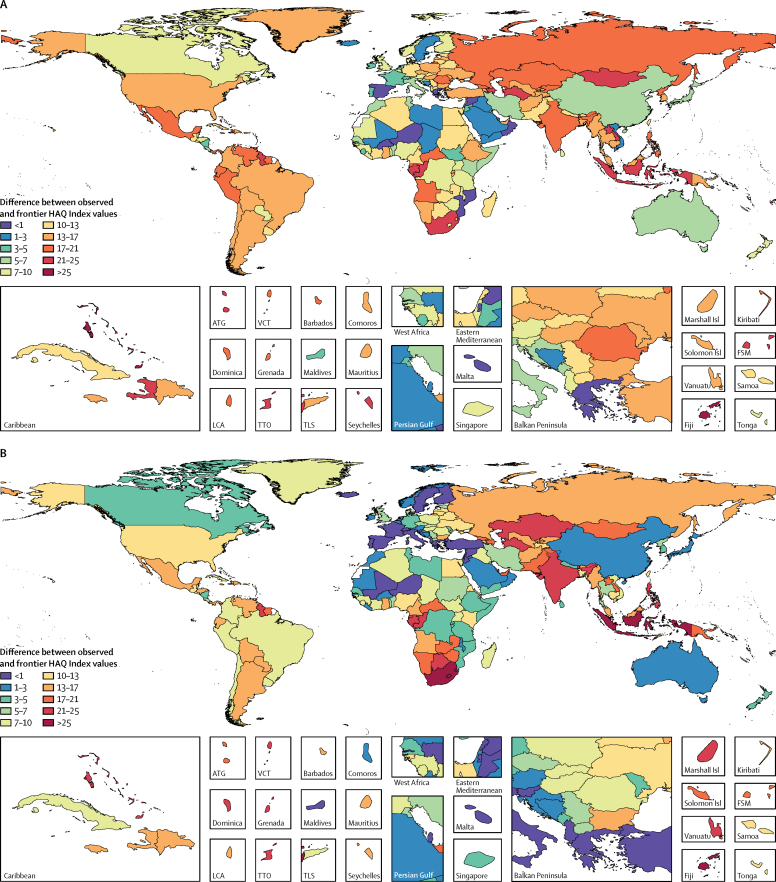
Map of the gap between observed HAQ Index and frontier values in 1990 (A) and 2015 (B) Difference in observed HAQ Index and frontier values were the highest levels achieved by geographies of similar SDI in a given year. HAQ Index=Healthcare Access and Quality Index. SDI=Socio-demographic Index. ATG=Antigua and Barbuda. VCT=Saint Vincent and the Grenadines. LCA=Saint Lucia. TTO=Trinidad and Tobago. TLS=Timor-Leste. FSM=Federated States of Micronesia.

Worldwide, the average HAQ Index values significantly increased, but the average global frontier improved in tandem; subsequently, gaps between the global HAQ Index and frontier changed minimally between 1990 and 2015. While most regions recorded narrowing gaps between average HAQ Index values and maximum levels achieved, a subset saw negligible progress or widening differences (eg, southern sub-Saharan Africa, south Asia, and the Middle East). In 2015, 52 countries and territories had HAQ Index estimates that included the frontier within their uncertainty bounds, indicating these geographies met the maximum levels of personal health-care access and quality attained by locations of similar SDI. Conversely, 62 geographies fell further behind the HAQ Index frontier associated with their level of SDI; this trend was especially pronounced in much of southern sub-Saharan Africa, Iraq, Pakistan, and Honduras (figure 5). This result was in stark contrast with several countries in eastern and western sub-Saharan Africa (eg, Burundi, Comoros, Rwanda), Turkey, Peru, and South Korea, many of which more than halved the differences between their HAQ Index and frontiers given their SDI by 2015.

## Discussion

Drawing from GBD 2015, we constructed a novel measure of personal health-care access and quality—the HAQ Index—by using highly standardised estimates of 32 different causes that are amenable to personal health care. Compared with previous efforts, the HAQ Index provides a clearer signal on personal health-care access and quality over time and place because GBD provides enhanced comparability of cause of death data, helps to account for variation due to behavioural and environmental risk factors, and includes 195 countries and territories over time. Our analysis showed large differences in personal health-care access and quality, spanning from a low of 23·1 in Ethiopia in 1990 to higher than 90 in Andorra, Iceland, Switzerland, Norway, and Sweden in 2015. The global HAQ Index improved from 40·7 in 1990 to 53·7 in 2015, and 167 of 195 countries and territories significantly increased their HAQ Index during this time. Although the HAQ Index and SDI were highly correlated, we noted substantial heterogeneity for geographies at similar SDI. If every location reached the highest observed HAQ Index experienced by level of SDI, our global measure of health-care access and quality could have reached 73·8 in 2015—a clear indicator of untapped potential for health-care improvement worldwide.

While most countries saw progress on the HAQ Index since 1990, the marked improvements recorded for countries including South Korea, Turkey, and China highlight that much more rapid advances are possible. A subset of countries narrowed the gap between observed personal health-care access and quality and what could be expected given their level of development—and then achieved gains beyond what might be anticipated on the basis of SDI. Peru, the Maldives, and Ethiopia are examples of such stand-out geographies for reaching higher-than-expected levels of personal health care and access since 1990. Case studies conducted by the World Bank highlight potential drivers of these countries' successes,^60^ and additional research on how certain health-system attributes, including financing arrangements, provider ownership, and stewardship functions, are related to personal health-care access and quality is warranted. Separating out measures of access from quality of care received would be ideal, especially because programmatic and policy options to address barriers in access and quality can differ across and within countries. Nonetheless, such information measured in a consistent manner is rarely available.^34^, ^46^, ^57^, ^60^

Several geographies had minimal gains in reducing the difference between their observed HAQ Index and the highest levels achieved at a similar SDI—a warning sign that heightened health-care access and quality is not an inevitable product of increased development. Further, a subset of countries in southern sub-Saharan Africa, south Asia, and the Middle East saw widening gaps between HAQ Index values in 2015 and the frontier reached by countries of comparable SDI. These findings could reflect several challenges faced by these countries, including subnational inequalities in both wealth and health-care options; and recent or rapid epidemiological transitions wherein the health-care sector and cause-specific services offered trail behind the diseases afflicting populations. Along with examining the drivers of greater-than-expected gains, future studies should strive to understand why other countries are lagging behind—and how they can pursue advancing health-care access and quality for all individuals.

Improving personal health-care access and quality is an important priority in the SDG era, emphasising the potential utility of the HAQ Index for SDG monitoring. At present, the UHC target—SDG 3.8—focuses mainly on so-called tracer interventions in the areas of maternal and child health, reproductive health, and a subset of infectious diseases,^61^ and thus fails to adequately capture the vital role of personal health care for NCDs and injuries. The HAQ Index provides a more comprehensive measure that reflects health-system capacity for effectively detecting risk for, managing, and preventing early death from a range of conditions. Combining the HAQ Index, coverage of health interventions, and prevalence of risk factors that are modifiable through public health initiatives could provide a more robust mechanism for tracking UHC progress across multiple dimensions of health-system action.

Health systems can provide differential access and quality across therapeutic areas and levels of care. The HAQ Index reflects the average experience as captured by included conditions, and does not currently distinguish between diseases more strongly related to primary or secondary care. Our PCA weights suggest some conditions are less highly correlated with other causes, including colon cancer, breast cancer, testicular cancer, non-melanoma skin cancer, or diphtheria. The comparatively low weights on these outcomes could reflect measurement error, residual challenges in risk standardisation, or health-system heterogeneity by level of care. Subnational work that identifies variation in personal health-care access and quality within a particular health-system structure, and that uses multi-method approaches to view the health system from the perspective of patients and frontline providers,^62^, ^63^ may help elucidate whether some health-system components function distinctly from its average. For example, access to and quality of oncology care might be relatively distinct from other health-system dimensions and, where appropriate treatment is contingent on specialists or particular equipment, such as radiotherapy for cancer, even a temporary loss of these resources may substantially affect outcomes.^64^ Conversely, access to high-quality primary care services, which enable early detection of conditions that are fatal if diagnosed at later stages, can be shaped by different factors, such as flexibility of clinic hours or types of insurance providers accepted.^65^

### Mortality amenable to personal health care and mortality attributable to modifiable behavioural and environmental risk factors

For the present study, we based the HAQ Index on the list of causes established by Nolte and McKee,^4^, ^9^, ^11^, ^30^, ^31^, ^35^ and did not systematically re-examine scientific literature to update causes for which personal health care can significantly improve outcomes. Conducting this kind of systematic review is crucial to identifying additional causes for inclusion in the HAQ Index. Numerous causes should be considered, and would likely result in adding antiretroviral therapy for HIV, artemisinin-based combination therapies for malaria, treatment of hepatitis C, and improvements in emergency and trauma care, among others.^41^, ^42^, ^66^, ^67^, ^68^, ^69^ Expanding the amenable cause list should be determined by clear criteria that define when health care sufficiently reduces cause-specific mortality and thus provides a strong enough signal about access and quality. Such additions will probably improve the HAQ Index, though the nature of PCA estimation and its measurement of common variance across 32 causes may not substantially change future results. This analysis stemmed from existing scientific literature on mortality amenable to health care, but personal health care also can have profound effects on non-fatal health outcomes (eg, hip replacement for oesteoarthritis or surgery for cataracts). Future updates of the HAQ Index should consider incorporating measures of non-fatal conditions amenable to personal health care, which would then capture health-system capacity to deliver health gains through improved functional health status.

Understanding how much mortality or disease burden is avertable based on providing access to high-quality personal health care and modifying behavioural and environmental risks through public health initiatives is of high policy interest. GBD currently assesses mortality and burden attributable to a large set of risk factors, which supplies useful insights on the potential of risk modification to improve health. Quantification of the full potential of personal health care to reduce burden by cause would provide an important additional piece of policy-relevant information. Controlling for other factors through statistical modelling, such as income and educational attainment, we could examine how much cause-specific variation relates to the HAQ Index. Such work would help to pinpoint opportunities for national and subnational progress through health-system improvements, which would likely include public health programmes and policies as well as the organisation and delivery of personal health services across levels of care.

### Moving to performance measurement

In estimating the HAQ Index frontier by SDI,^58^ we quantified the gap between observed personal health-care access and quality and levels potentially achievable at a given level of SDI. With these analyses, we lay the foundation for a refined assessment of health-system performance. The World Health Report 2000, which sought to evaluate health-system performance by country,^70^ estimated the contribution of health systems for improving healthy life expectancy while statistically controlling for other factors. As suggested by Nolte and McKee,^9^, ^71^ using a measure more directly related to health-system actions, such as mortality amenable to personal health care, could reduce the need to control for other factors in health-system performance assessment. The World Health Report 2000 framework used five broad dimensions—average levels of health, inequalities in health, average levels of health-system performance, inequalities in responsiveness, and fair financing—and then compared overall health system attainment based on a frontier for health expenditure per capita.^71^ Our current analysis only focused on the contribution of personal health care to mortality and the potential for improvement in this domain relative to development. In the future, GBD could support examining subnational health inequalities and expanding into health finance quantification of financial risk protection. A stronger empirical basis for assessing these three domains of health-system performance would also facilitate testing a range of efficiency and performance models.

Our frontier analysis showed that the highest observed HAQ Index levels, as achieved by geographies with an SDI of 0·8 or higher, steadily shifted higher over time. This expansion of health-care access and quality may reflect a rising share of GDP allocated to health among high-SDI countries. However, the frontier estimate for GDP per capita spent on health points to a similar shift upward at high expenditure. One explanation of this trend is new medical technologies and programmes, which could be driving an overall upward shift in health-care access and quality achievable in well financed systems. Another potential explanation is innovation in health-care organisation, such as the creation of centralised stroke care units in major cities.^72^ A more detailed examination of these changes may further elucidate how investing in medical innovations can affect health-system performance. In particular, this might shed light on the association between investment in health-care resources and outcomes, a relationship that is unlikely to be linear. For instance, audits have identified three main factors underlying maternal deaths: substandard care, delays in care, and problems with blood transfusions.^73^ Addressing the latter requires a different type of intervention, namely investments in infrastructure, than the former two factors. Such knowledge is of particular importance in the SDG era, as some studies point to advances in medical technology and innovation as the primary pathway for elevating health alongside increasing development.^74^

### HAQ Index compared to other measures of access and quality of care

Detailed results on HAQ Index components seem consistent with previous, albeit limited, studies on health-care performance. Within Europe, Nordic countries performed especially well, corresponding with past work on a composite measure of public health policies.^75^ Country performance on diabetes aligned with earlier work on diabetes mortality and incidence,^25^ wherein country-level differences were largely explained by known health-system changes, such as substantial improvements in several Baltic states during the late 1990s. In Latin America, Costa Rica's relatively high HAQ Index (72·9), as compared with nearby countries (eg, Nicaragua [64·3], Guatemala [55·7]), is consistent with its designation as an original “good health at low cost” country.^76^

In view of the paucity of standard health-care access and quality measures, assessing HAQ Index validity compared to other indicators was challenging. In this analysis, we identified three measures of health-system resources and three measures of intervention coverage that included at least 70 countries. These correlations, which all exceeded 0·60, offer some evidence of convergent validity but do not provide criterion validity.^77^, ^78^ Nonetheless, these results are encouraging and stand in contrast to previous studies done in limited settings.^37^, ^38^, ^39^, ^40^ In comparison with past work,^9^, ^11^, ^31^ the moderately high correlation with other health-care indicators might be due to our efforts to risk-standardise mortality amenable to health care; PCA weighting of different amenable conditions; and the inclusion of a substantively larger, more diverse set of health systems across the development spectrum. Additional validation analyses are needed to compare HAQ Index performance with other measures of health-care access and quality; such validation exercises might be more feasible at the subnational level with greater data density, such as states in the USA.^79^

### Limitations

This analysis has a number of limitations beyond those already described. First, many limitations experienced in GBD cause of death estimation are applicable to this study.^47^ Second, our risk-standardisation procedure might not represent all possible risk-outcome pairs as they pertain to included causes of amenable mortality (eg, determinants of testicular cancer or neonatal disorders).^48^ With its annual updates, GBD aims to improve upon its comparative risk assessment, and thus HAQ Index assessment is likely to be improved alongside advances in risk quantification. Third, two causes received negative weights in the PCA analysis and were subsequently excluded. One potential explanation for this is that joint PAFs for these causes may underestimate risk-attributable mortality in high-SDI countries (eg, the effects of diet, obesity, and physical inactivity for breast cancer). However, given the high Spearman's rank order correlation between the average of all 32 causes and the HAQ Index, excluding these causes from the PCA likely had minimal effect on our results. Fourth, we used PCA to construct the HAQ Index based on age-standardised risk-standardised death rates from the 32 causes. Alternative methods for index construction led to highly correlated results, but exact rankings somewhat varied. We subsequently view exact rank orders as less useful than comparing a given geography's HAQ Index values over time, to countries of similar SDI, and relative to the HAQ Index frontier. Fifth, while the HAQ Index offers a more robust indicator of overall health-care access and quality than currently available measures, it does not directly capture effects of personal health care on causes without substantial mortality (eg, depression, hip oesteoarthritis, and cataracts). The effects of health care on both fatal and non-fatal conditions may be highly correlated, but incorporating how access and quality of care explicitly affect non-fatal outcomes would improve measurement. Sixth, GBD corrections for cause of death misclassifications (so-called garbage codes) varies substantially by geography and thus can affect results. Even among high-SDI countries, GBD showed substantial variation for the proportion of amenable deaths assigned to garbage codes, ranging from 7·9% in Finland to 39·8% in Portugal (appendix p 19). Seventh, for countries with complete or nearly complete vital registration (VR) data and few deaths misclassified based on ICD codes, the HAQ Index may be more robust and less prone to high levels of uncertainty than for countries with lower-quality or non-existent VR data. Mortality estimates that heavily draw from verbal autopsy data or other modelling approaches have larger UIs. Our results for most of sub-Saharan Africa, for example, include wide UIs and thus few countries recorded HAQ Index values that statistically differed from the regional mean. Eighth, we rescale the log age-standardised risk-standardised death rate for each cause from 0 to 100 using the observed range across countries from 1990 to 2015, but achieving 100 does not mean that additional improvement is not possible. Subsequently, the HAQ Index range reported here is relative to national achievements to date, and these thresholds may rise if or when improved personal health-care access and quality occurs for given causes. Ninth, the HAQ Index does not currently capture subnational inequalities in personal health-care access and quality, which might emerge on the basis of geographic location or socioeconomic status, among other factors. Future efforts to quantify these measures with greater geospatial resolution should be prioritised.

## Conclusions

Our analysis demonstrates that a policy-relevant summary measure of personal health-care access and quality can be derived from GBD. This novel measure supports the first-ever comparable assessment of personal health-care access and quality across 195 countries and territories, over time, and along the development spectrum. The HAQ Index considerably advances previous efforts to approximate personal health-care access and quality by systematically adjusting for cause of death certification biases and misclassification, risk-standardising death rates across geographies, and applying PCA to identify common dimensions of health-care access and quality associated with multiple conditions. Globally, most countries and territories recorded gains in personal health-care access and quality from 1990 to 2015, yet many still experienced levels that fell well below what has been achieved by geographies at a similar development status. Amid calls to improve monitoring of UHC and overall health-system performance, the HAQ Index provides a strong basis for benchmarking progress toward greater access and higher-quality personal health care alongside country-level gains in resources to achieve these aims.
