# Supplementary material for: Healthcare Access and Quality Index based on mortality from causes amenable to personal health care in 195 countries and territories, 1990–2015: a novel analysis from the Global Burden of Disease Study 2015
Source: Lancet. 2017 Jul 15;390(10091):231–66. doi: 10.1016/S0140-6736(17)30818-8 (PMC5528124; doi:10.1016/S0140-6736(17)30818-8)
Supplement: Supplementary appendix [file mmc1.pdf]

THE LANCET

Supplementary appendix

This appendix formed part of the original submission and has been peer reviewed. We post it as supplied by the authors.

Supplement to: GBD 2015 Healthcare Access and Quality Collaborators. Healthcare Access and Quality Index based on mortality from causes amenable to personal health care in 195 countries and territories, 1990–2015: a novel analysis from the Global Burden of Disease Study 2015. *Lancet* 2017; published online May 18. http://dx.doi.org/10.1016/S0140-6736(17)30818-8.

Appendix to Healthcare Access and Quality Index based on mortality from causes amenable to personal healthcare in 195 countries and territories, 1990–2015: a novel analysis from the Global Burden of Disease 2015 study

This appendix provides further methodological detail, supplemental figures, and more detailed results for constructing the Healthcare Access and Quality Index. The appendix is organized into broad sections following the structure of the main paper.

Table of Contents

Preamble 3

Part 1. Estimating the Healthcare Access and Quality Index 5

Section 1. Healthcare Access and Quality Index overview

Section 2. Overall modelling strategy for the Healthcare Access and Quality Index

Part 2. Producing the Healthcare Access and Quality Index frontier 9

Section 1. Frontier analysis overview

Section 2. Testing stochastic methods

Part 3. List of abbreviations 13

Figures and Tables

Supplementary Figure 1. Effect of risk-standardisation on amenable causes.

Supplementary Figure 2. Relationship between risk- and age-standardised mortality among amenable causes and the Healthcare Access and Quality Index.

Supplementary Table 1. GATHER checklist of information that should be included in reports of global health estimates, with description of compliance and location of information for Healthcare Access and Quality Index.

Supplementary Table 2. List of ICD codes mapped to GBD 2015 causes for which mortality is amenable to healthcare. ICD=International Classification of Diseases. GBD = Global Burden of Disease.

Supplementary Table 3. Percent of deaths amenable causes from garbage codes among OECD countries reporting ICD9 and ICD10 in 2010.

Supplementary Table 4. Global mean, standard deviation, and skewness of the log of risk-standardised age-standardised death rates by cause amenable to healthcare. These values represent 195 countries and territories in 1990, 1995, 2000, 2005, 2010, and 2015. Skewness reflects the magnitude of asymmetry of values from the mean.

Supplementary Table 5. Factor loadings and weights from principal component analysis and exploratory factor analysis.

Supplementary Table 6. Country and territory-level frontier values of the HAQ Index by five-year interval from 1990 to 2015. Geographies are organised GBD super-region and region. HAQ Index = Healthcare Access and Quality Index. GBD = Global Burden of Disease.

Preamble

This appendix provides methodological detail estimating for the Healthcare Access and Quality Index and its frontier. The appendix is organized into broad sections following the structure of the main paper. This study complies with the Guidelines for Accurate and Transparent Health Estimates Reporting (GATHER) recommendations. It includes detailed indicator modeling write-ups and flowcharts, and information on data sourcing in an effort to maximise transparency in our estimation processes and provide a comprehensive account of analytical steps. We intend this to be a living document, to be updated with each annual iteration of the Global Burden of Diseases, Injuries, and Risk Factors study (GBD).

Part 1. Estimating Healthcare Access and Quality Index

Section 1. Healthcare Access and Quality Index overview

Measuring mortality rates due to causes which are considered amenable to healthcare is way to approximate average levels personal healthcare access and quality. Based on cause of death data and risk exposure data and estimates from the Global Burden of Diseases, Injuries, and Risk Factors study 2015 (GBD 2015),[1,2] we expand previous analyses of mortality amenable to healthcare, namely that of Nolte and McKee,[3–7] and construct the Healthcare Access and Quality Index (HAQ Index) for 195 countries and territories from 1990 to 2015.

Section 2. Overall modelling strategy for the Healthcare Access and Quality Index

The methodology used in estimating the HAQ Index is visualised in the flowchart below.

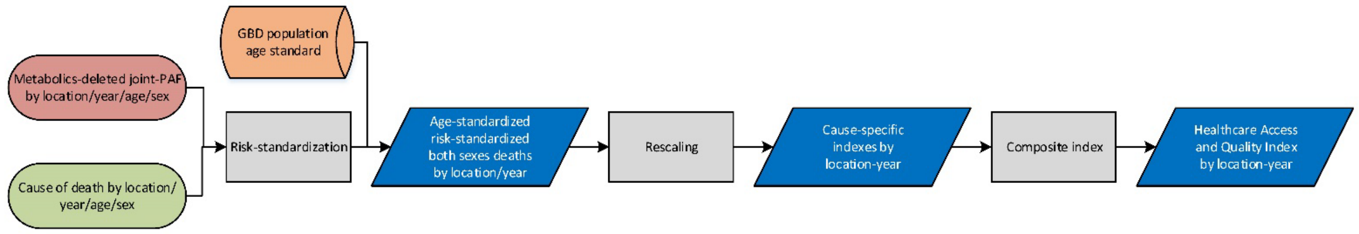

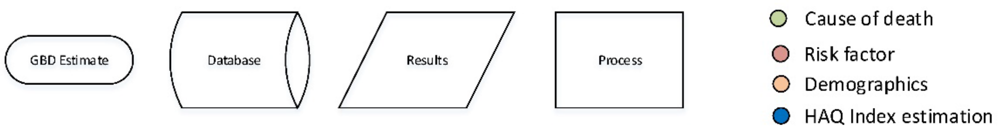

Input data and modelling strategies

The inputs to the HAQ Index were results from the GBD 2015 cause-specific mortality and comparative risk assessment,[1,2] representing 519 distinct geographic units, 20 age groups, two sexes, and six years spanning a 25-year range (1990, 1995, 2000, 2005, 2010, and 2015). For each input, 1,000 draws were used in order to estimate uncertainty.

Input data and modelling strategies for estimating cause-specific mortality and population attributable fractions (PAFs) are detailed in GBD 2015 appendices,[1,2] and both input data and estimates can be downloaded through the Global Health Data Exchange (GHDx): http://ghdx.healthdata.org/gbd-2015 Location-year-age-sex-specific cause of death estimates from GBD 2015 are used directly. We estimated a joint-risk (PAF) for each cause, using all risks except metabolic risks, which are so directly linked to personal healthcare, and thus would not be appropriate for risk-standardisation steps explained later. Assumptions about how one risk factor is mediated through other risk factors are needed in order to estimate the joint-risk factor burden for combinations of behavioural or environmental risks. To accomplish this, for every two risk factors for an outcome, we used published studies to estimate the

fraction of risk that was mediated through the other risk. This resulted in a matrix of parameters containing each possible pairing of risk factors included in the GBD 2015.[2] Using this matrix, we computed the aggregated burden of disease at each level of the GBD 2015 hierarchy and for all risk factors using the following formula:

$$PAF_{J,o,a,s,g,t} = 1 - \prod_{j=1}^{J}\left(1 - PAF_{j,o,a,s,g,t}\prod_{i=1}^{J}(1 - MF_{j,i,o})\right)$$

where J is a set of risk factors for the aggregation; $PAF_{j,o,a,s,g,t}$ is the PAF for risk j for cause o, age group a, sex s, geography g, and year t; and $MF_{j,i,o}$ is the mediation factor for risk j mediated through i for cause o.

Risk-standardisation and rescaling

The risk standardisation process is a key innovation of this work on amenable mortality. The aim of this process is to eliminate the residual effects of localised risk exposure that would otherwise act as a confounding element in our ability to draw inferences about healthcare from mortality due to amenable causes. By imposing a global level of exposure on all geographies, we de-contextualise them and create a measure of mortality that is a more appropriate proxy for healthcare access and quality. Death rates are risk-standardised using the formula:

$$RSD_{l,y,a,s} = D_{l,y,a,s} \times \left(1 - PAF_{l,y,a,s}\right) \times \frac{1}{1 - GPAF_{a,s}}$$

where $RSD_{l,y,a,s}$ is the risk-standardised death rate in location l, year y, age group a, and sex s; $D_{l,y,a,s}$ is the death rate for those specifications; $PAF_{l,y,a,s}$ is the PAF for those specifications; and $GPAF_{a,s}$ is the global PAF over all six estimation years for age group a, and sex s. If for a given cause no risk attribution is present or all deaths are attributed to risks (ie, PAF of 0 or 1), the observed deaths are used. Also, for diarrhoeal diseases, observed deaths are used due to the fact that no PAF is estimated for water and sanitation in high income countries.

If any cause has a maximum observed mean joint-risk PAF greater than 0.9 but less than 1 for a given age and sex, PAFs across all location years are scaled such that the maximum is scaled down to 0.9. Deaths outside of the ages defined in Table 1 in the main text were eliminated, as only deaths in those ages were deemed highly amenable to healthcare. We then aggregated cause-specific mortality rates by sex to both sexes. Using the GBD population age standard, we compiled age-standardised risk-standardised mortality rates for both sexes by location, year, and amenable cause:

$$RSASD_{l,y} = \sum_{a=1}^{n} RSD_{l,y,a} \times PAS_a$$

here $RSASD_{l,y,d}$ is the age-standardised risk-standardised death rate for location l, and year y; $RSD_{l,y,a}$ is the risk-standardised death rate in location l, year y, and age group a; and $PAS_a$is the population age standard for age group a.

In order to have a standardised score of amenable mortality by cause across location-years, we then take the age-standardized risk-standardised death rates and convert them to a 0 to 100 index value:

$$I_{l,y} = \frac{\log(RSASD_{l,y}) - \min(\log(RSASD'_{CY}))}{\max(\log(RSASD'_{CY})) - \min(\log(RSASD'_{CY}))}$$

where $I_{l,y}$ and $RSASD_{l,y}$ are the cause-specific index values and age-standardised risk-standardised death rate for location l and year y, respectively. $RSASD'_{CY}$ is the matrix age-standardised risk-standardised death rates for all countries with populations over one million for each of the six years. We apply the limitation of population size for the purpose of eliminating elongated tails in the location-year distribution, which leads to clustering of the indexed values nearer to the center. In order to eliminate zeroes while maintaining the observed range, we added one death per 1 million population to all values before log transformation.

Creating composite indicator

Using the above methodology, we have created 1,000 draws of location-year index values for 32 causes of death amenable to healthcare. We aimed to create a composite measure using the 32 components, the HAQ Index. We tested four different methods of combination: principal components analysis (PCA), exploratory factor analysis (EFA), arithmetic mean, and geometric mean.

For PCA and EFA, we first took the mean value of the 1,000 draws. We then produced a zero-centered matrix in which rows represented all 519 most-detailed geographic units from GBD and all six estimation years, and columns the 32 causes of death included in analysis. We aimed to use the factor loadings of these causes as weights in creating our summary indicator. The number of factors for which we retained loadings was determined on the basis that their cumulative variance explained exceeded 80%. Using those n factors, we created one composite factor by taking the average loading across factors, weighted by their explained variance. We would perform this analysis two times – after the first PCA or EFA, we then eliminated causes for which the composite loading was in the opposite direction of the majority. In both methods, this resulted in the removal of colon and rectum cancer from consideration. Then, we performed the analysis again using the remaining 31 causes. In the PCA, the second iteration resulted in breast cancer also being eliminated. We next rescaled the composite loading to sum to 1 – these values would be used as weights in generating the summary indicator:

$$SI_{l,y} = \sum_{c=1}^{n} I_{l,y,c} \times weight_c$$

where $SI_{l,y}$, the summary indicator for location l and year y, is equal to the sum of the product of $I_{l,y,c}$ (index value for location l, year y, and cause c) and the weight for cause c across causes.

We then used these four summary indicators and evaluated them on convergence validity. We selected available six measures deemed relevant to healthcare access and quality: two indicators from the Institute for Health Metrics and Evaluation covariate database for GBD, health expenditure per capita and hospital beds per capita that covered 195 geographies and all years under analysis; universal health coverage tracer index composed of 11 individual interventions that covered 188 geographies and all years under analysis;[8] physicians, nurses, and midwives per capita in 2010 for 73 countries from the

WHO;[9] proportion of population with formal health coverage for 93 countries from the International Labour Organization;[10] and the Coverage Index for performance on primary health care service coverage (World Bank, 123 countries in 2015).[11] For each of these, the indicator created from the PCA weights had the highest Spearman rank order correlation coefficient.

References

1 Wang H, Naghavi M, Allen C, *et al.* Global, regional, and national life expectancy, all-cause mortality, and cause-specific mortality for 249 causes of death, 1980–2015: a systematic analysis for the Global Burden of Disease Study 2015. *The Lancet* 2016; **388**: 1459–544.

2 Forouzanfar MH, Afshin A, Alexander LT, *et al.* Global, regional, and national comparative risk assessment of 79 behavioural, environmental and occupational, and metabolic risks or clusters of risks, 1990–2015: a systematic analysis for the Global Burden of Disease Study 2015. *The Lancet* 2016; **388**: 1659–724.

3 Nolte E, McKee M. Measuring the health of nations: analysis of mortality amenable to health care. *BMJ* 2003; **327**: 1129.

4 Nolte E, McKee M. Does healthcare save lives? Avoidable mortality revisited. London, UK: Nuffield Trust, 2004.

5 Nolte E, McKee CM. Measuring The Health Of Nations: Updating An Earlier Analysis. *Health Aff (Millwood)* 2008; **27**: 58–71.

6 Nolte E, McKee M. Variations in amenable mortality--trends in 16 high-income nations. *Health Policy Amst Neth* 2011; **103**: 47–52.

7 Nolte E, McKee CM. In Amenable Mortality—Deaths Avoidable Through Health Care—Progress In The US Lags That Of Three European Countries. *Health Aff (Millwood)* 2012; **31**: 2114–22.

8 Lim SS, Allen K, Bhutta ZA, *et al.* Measuring the health-related Sustainable Development Goals in 188 countries: a baseline analysis from the Global Burden of Disease Study 2015. *The Lancet* 2016; **388**: 1813–50.

9 World Health Organization (WHO). Global Health Observatory. WHO. http://www.who.int/gho/database/en/ (accessed Sept 10, 2015).

10 International Labour Organization (ILO). World Social Security Report 2010/2011: Providing coverage times of crisis and beyond. Geneva, Switzerland: ILO, 2010.

11 World Bank. Coverage index database.

Part 2. Producing the Healthcare Access and Quality Index frontier

Section 1. Frontier analysis overview

We aimed to produce a frontier that describes the maximum achievable level of the Healthcare Access and Quality Index (HAQ Index) across the spectrum of development, as measured by the Socio-demographic index (SDI). We developed a novel approach to frontier analysis after testing several standard methods. Stochastic frontier analysis (SFA) methods did not adequately capture the nonlinear nature of the frontier,[1] and data envelopment analysis (DEA) methods make a strong assumption that the data lack measurement error.[2] Instead, we used a flexible type of DEA called free disposal hull (FDH) analysis and modified it to incorporate measurement error.

Section 2. Testing stochastic methods

SFA can be understood intuitively as an extension of ordinary least squares (OLS) regression. The frontier is defined by a set of parameters $\beta_0 \ldots \beta_n$ generally following the form of:

$$y_i = \beta_0 + \sum_n \beta_n x_{n,i} + v_i - u_i$$

where y is the output of interest, x is an input, v represents the "noise" term, and u represents the inefficiency term. The noise term v_i has the same interpretation as the error term in OLS regression and is assumed to be normally distributed. The inefficiency term u_i describes the difference between each point and its efficient maximum, and may follow any distribution bounded by zero (eg, truncated normal, exponential). Together, v_i minus u_i forms a compound error term that describes the departure of each point from the frontier.

We tested several forms for the production function relating SDI to the HAQ Index, including linear, log-log (Cobb-Douglas), and translog. All functional forms had a problem with heteroscedasticity, especially in the earlier versions of the HAQ Index. Similar to OLS regression, heteroscedasticity in frontier analysis arises when the data have a nonlinear shape that is difficult to capture in a parametric model. For that reason, we tested additional methods that allow for more flexibility in the shape of the frontier.

Input data and modelling strategies

DEA is a non-parametric type of frontier analysis. DEA methods do not require a functional form for the relationship between variables, as SFA does. The frontier is produced from a deterministic algorithm. The figure below shows examples of DEA frontiers using various algorithms.

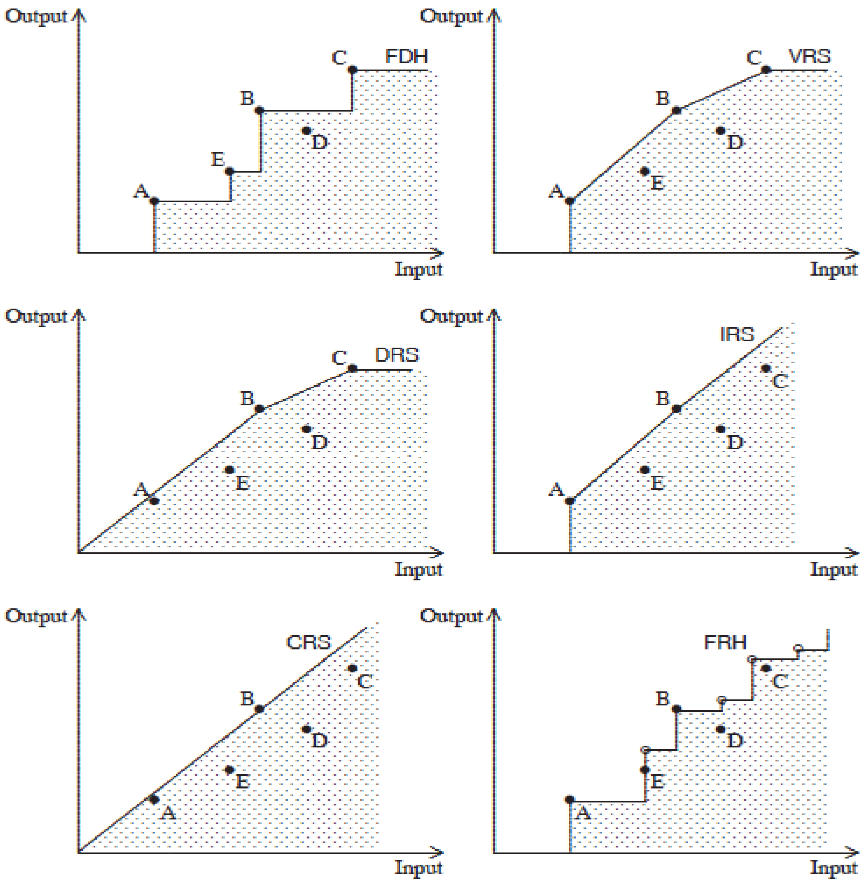

We chose to use the FDH option for this analysis. In contrast to the commonly-used “variable returns to scale” option, FDH relaxes the requirement that the frontier be convex. This is an important property, because the frontier in our data appears convex in some places and concave in others. The figure below shows the FDH method applied to our data.

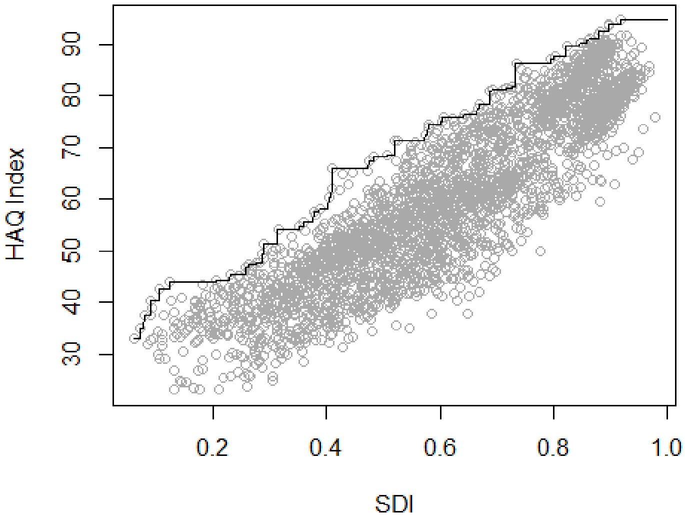

In general, the main limitation of DEA methods is that they assume a lack of stochastic variation.[2] That assumption does not hold for this analysis, which is based upon population-level metrics that inherently have a degree of measurement error. To incorporate stochastic variation into the frontier, we used 1,000 bootstrapped samples of the data. Each bootstrap includes a subset of locations produced by randomly sampling with replacement from all countries and subnational regions in the Global Burden of Disease study. This accounts for autocorrelation of locations over time. The value of the HAQ Index was drawn from the uncertainty distribution surrounding the HAQ Index for each location-year. Outliers were removed by excluding units with super-efficiency greater than 1. We took the mean of the bootstrapped samples at each level of SDI (nearest 0.001). Finally, we used Loess regression on this result to produce a smooth frontier.

The figure below shows year-specific frontiers using the bootstrapped FDH method. Note that the variance of data points above the frontier is not heteroscedastic, and the frontier is allowed to be nonlinear where appropriate.

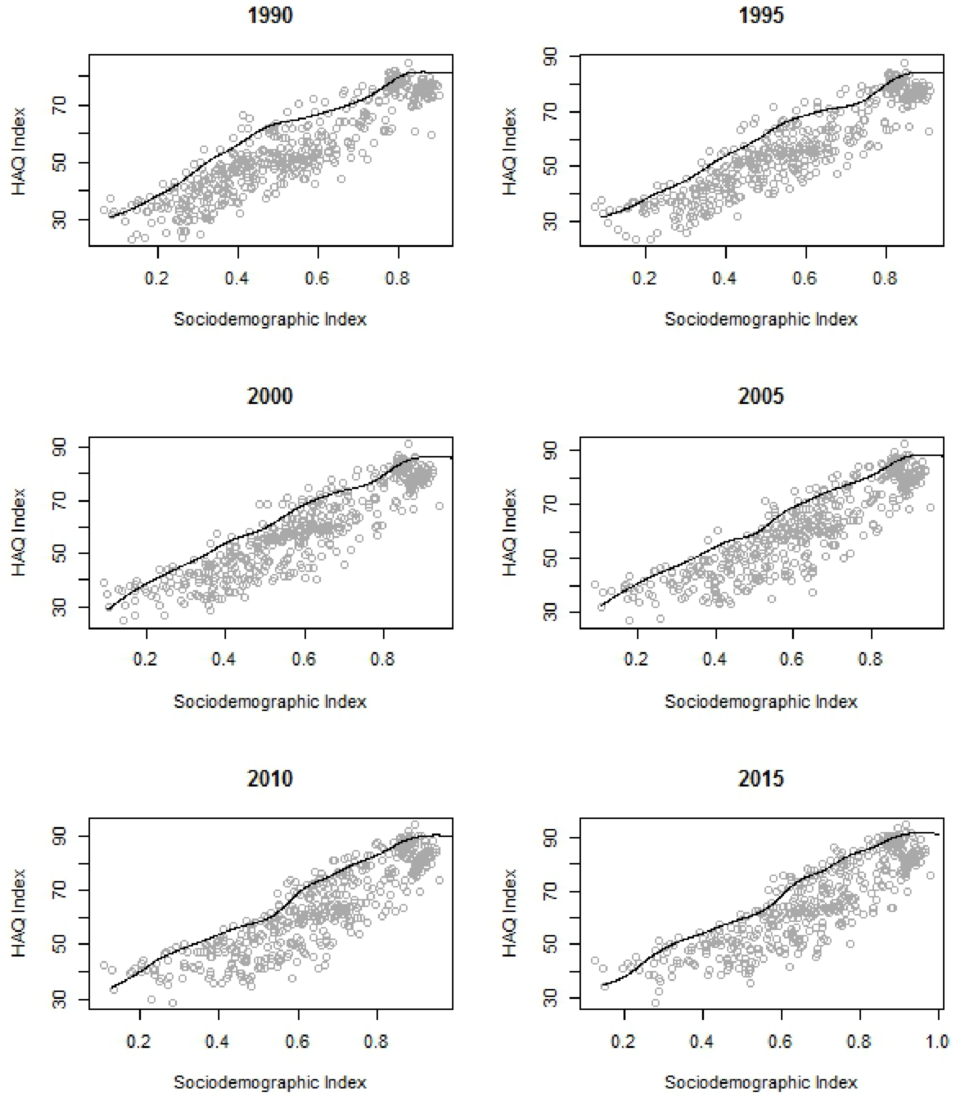

References

1 Bogetoft P, Otto L. Benchmarking with DEA, SFA, and R, 2011 edition. New York, NY, USA: Springer, 2013.

2 Hwang S-N, Lee H-S, Zhu J. Handbook of Operations Analytics Using Data Envelopment Analysis. Springer, 2016.

Part 3. List of abbreviations

BMI	Body-mass index
DALY	Disability-adjusted life-year
DEA	Development envelopment analysis
DPT3	Diphtheria-pertussis-tetanus, three doses
EFA	Exploratory factor analysis
FDH	Free disposal hull
GATHER	Guidelines for Accurate and Transparent Health Estimates Reporting
GBD	Global Burden of Disease
HALE	Healthy life expectancy
HAQ Index	Healthcare Access and Quality Index
ICD	International Classification of Diseases
ILO	International Labour Organization
LRIs	Lower respiratory infections
NCDs	Non-communicable diseases
OLS	Ordinary least squares
PAF	Population attributable fraction
PCA	Principal component analysis
SDGs	Sustainable Development Goals
SDI	Socio-demographic Index
SFA	Stochastic frontier analysis
UHC	Universal health coverage
UI	Uncertainty interval
WHO	World Health Organization
WHR	World Health Report

Supplementary Figure 1. Effect of risk−standardisation on amenable causes

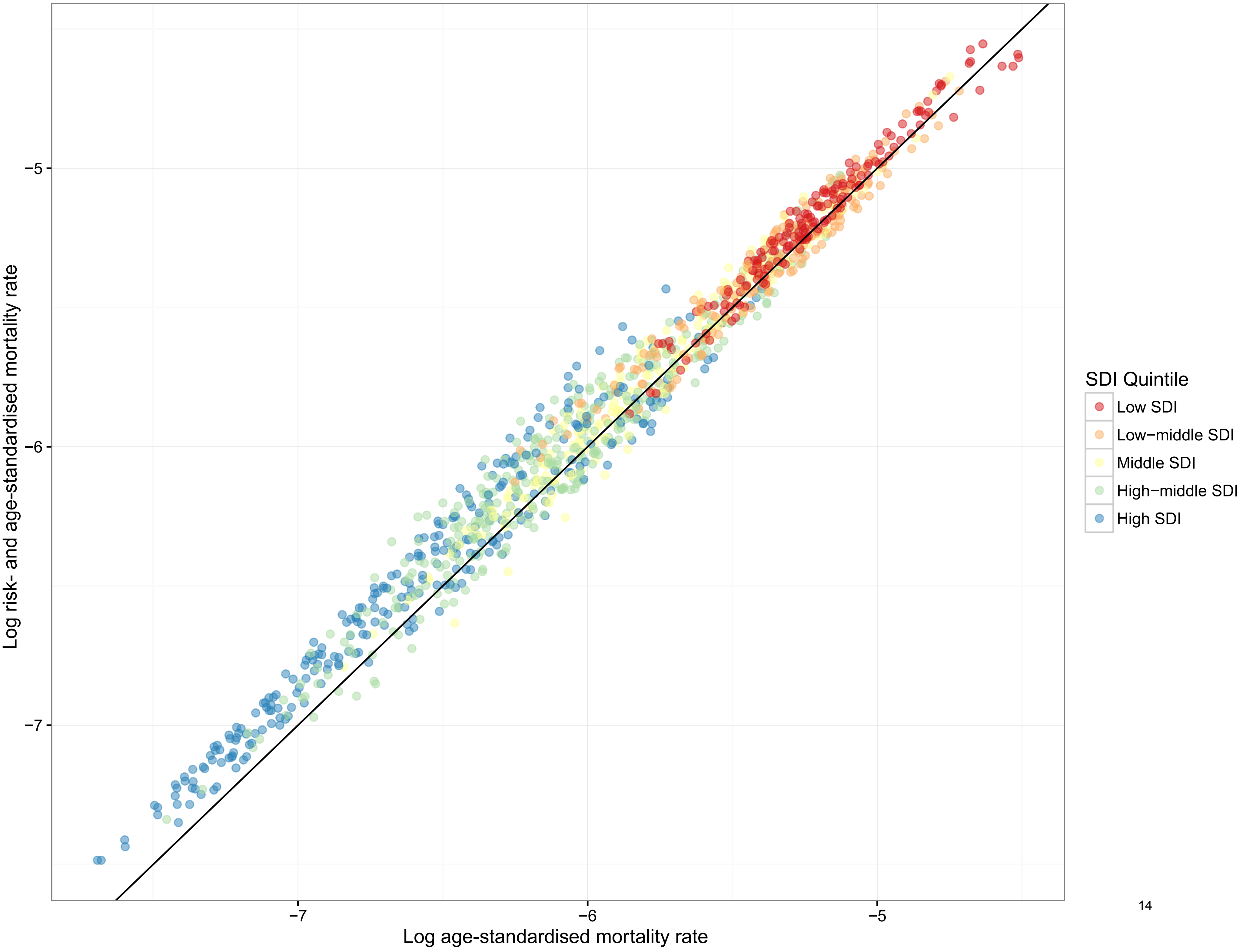

Supplementary Figure 2. Relationship between risk- and age-standardised mortality among amenable causes and the Healthcare Access and Quality Index

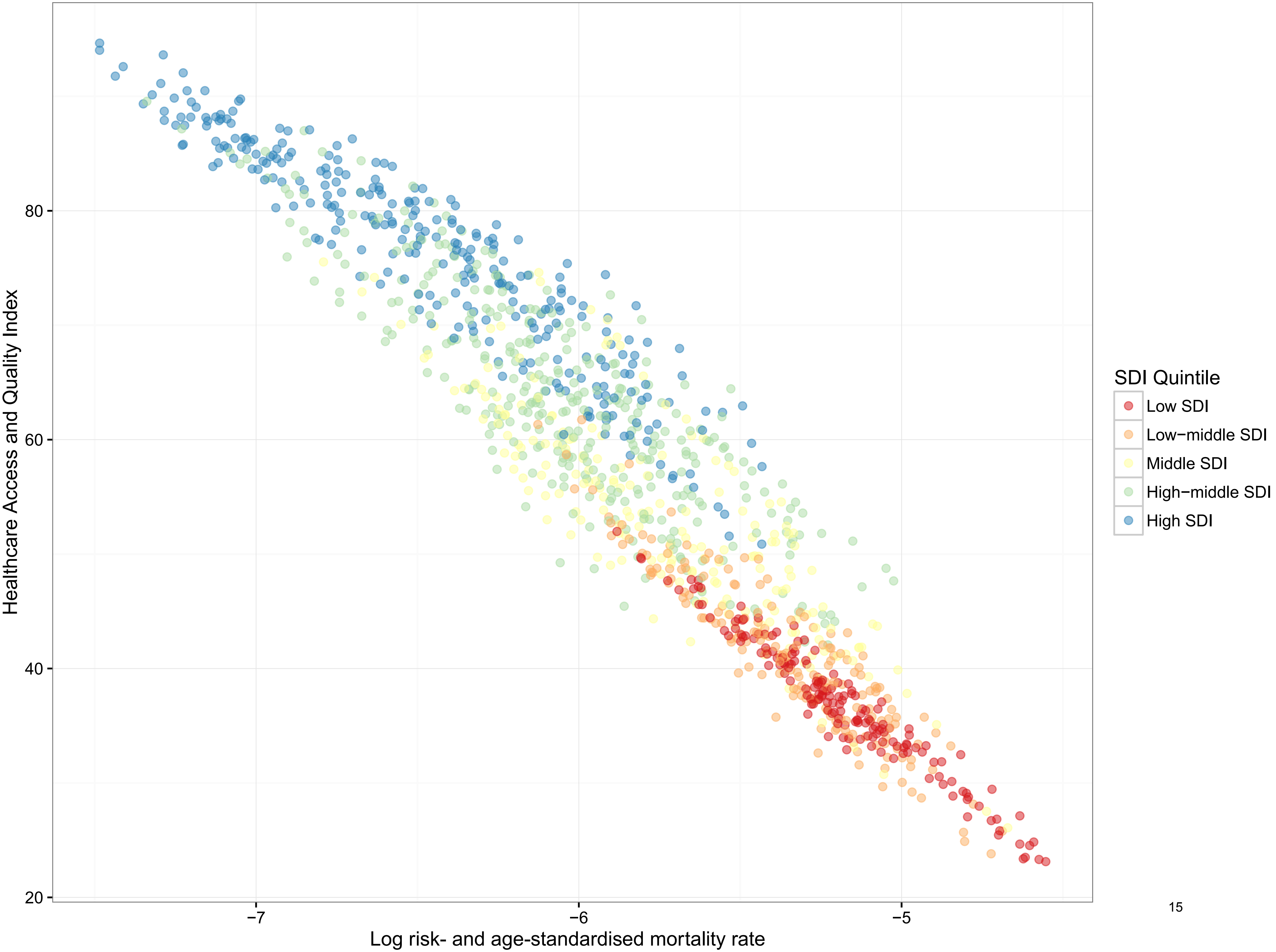

Supplementary Table 1. GATHER checklist of information that should be included in reports of global health estimates, with description of compliance and location of information for Healthcare Access and Quality Index.

#	GATHER checklist item	Description of compliance	Reference
**Objectives and funding**			
1	Define the indicators, populations, and time periods for which estimates were made.	Narrative provided in paper and appendix describing indicators, definitions, and populations.	Summary; Main text; Appendix Part 1
2	List the funding sources for the work.	Funding sources listed in paper.	Main text
**Data Inputs**			
For all data inputs from multiple sources that are synthesized as part of the study:			
3	Describe how the data were identified and how the data were accessed.	Narrative description of data seeking methodology provided.	Main text; Appendix Part 1
4	Specify the inclusion and exclusion criteria. Identify all ad-hoc exclusions.	Narrative about inclusion and exclusion criteria by data type provided.	Main text; Appendix Part 1
5	Provide information on all included data sources and their main characteristics. For each data source used, report reference information or contact name/institution, population represented, data collection method, year(s) of data collection, sex and age range, diagnostic criteria or measurement method, and sample size, as relevant.	List of all data sources provided in submission materials; interactive, online data source tool that provides metadata for data sources by component, geography, cause, risk, or impairment has been developed.	Main text; Appendix Part 1<br><br>http://ghdx.healthdata.org/gbd-2015/data-input-sources
6	Identify and describe any categories of input data that have potentially important biases (e.g., based on characteristics listed in item 5).	Summary of known biases by cause included in methodological appendix.	Appendix Part 1
For data inputs that contribute to the analysis but were not synthesized as part of the study:			
7	Describe and give sources for any other data inputs.	Included in list of all data sources provided in submission materials as well as online data source tool.	http://ghdx.healthdata.org/gbd-2015/data-input-sources
For all data inputs:			
8	Provide all data inputs in a file format from which data can be efficiently extracted (e.g., a spreadsheet as opposed to a PDF), including all relevant meta-data listed in item 5. For any data inputs that cannot be shared due to ethical or legal reasons, such as third-party ownership, provide a contact name or the name of the institution that retains the right to the data.	Downloads of input data will be available through online tools, including data visualization tools and data query tools. Input data not available in tools will be made available upon request.	Online data tools http://ghdx.healthdata.org/; http://ghdx.healthdata.org/gbd-data-tool

**Data analysis**			
9	Provide a conceptual overview of the data analysis method. A diagram may be helpful.	Flow diagrams of the overall methodological processes, as well as cause-specific modelling processes have been provided.	Main text; Appendix Part 1
10	Provide a detailed description of all steps of the analysis, including mathematical formulae. This description should cover, as relevant, data cleaning, data pre-processing, data adjustments and weighting of data sources, and mathematical or statistical model(s).	Flow diagrams and corresponding methodological write-ups for each cause and modelling processes have been provided.	Appendix Part 1
11	Describe how candidate models were evaluated and how the final model(s) were selected.	Provided in the methodological write-ups.	Appendix Part 1
12	Provide the results of an evaluation of model performance, if done, as well as the results of any relevant sensitivity analysis.	Provided in the methodological write-ups.	Appendix Part 1
13	Describe methods for calculating uncertainty of the estimates. State which sources of uncertainty were, and were not, accounted for in the uncertainty analysis.	Provided in the methodological write-ups.	Appendix Part 1
14	State how analytic or statistical source code used to generate estimates can be accessed.	Access statement provided.	This will be available in an online repository that will be released upon publication.
**Results and Discussion**			
15	Provide published estimates in a file format from which data can be efficiently extracted.	Results will be made available through the Global Health Data Exchange.	http://ghdx.healthdata.org/;
16	Report a quantitative measure of the uncertainty of the estimates (e.g. uncertainty intervals).	Uncertainty intervals are provided with all results.	http://ghdx.healthdata.org/;
17	Interpret results in light of existing evidence. If updating a previous set of estimates, describe the reasons for changes in estimates.	Discussion of methodological changes between GBD rounds provided in the narrative of the paper and appendix.	Research in Context; Main text; Appendix Parts 1 and 2
18	Discuss limitations of the estimates. Include a discussion of any modelling assumptions or data limitations that affect interpretation of the estimates.	Discussion of limitations provided in the narrative of the main paper as well as in the methodological write-ups in the appendix.	Main text; Appendix Parts 1 and 2

***Supplementary Table 2.* List of ICD codes mapped to GBD 2015 causes for which mortality is amenable to healthcare.** ICD=International Classification of Diseases. GBD = Global Burden of Disease.

Cause	ICD-10	ICD-9
**Communicable, maternal, neonatal, and nutritional diseases**		
Tuberculosis	A10-A14, A15-A19.9, B90-B90.9, K67.3, K93.0, M49.0, P37.0	A10-A14, A15-A19.9, B90-B90.9, K67.3, K93.0, M49.0, P37.0
Diarrhoea, lower respiratory, and other common infectious diseases		
Diarrhoeal diseases	A00-A00.9, A02-A04.1, A04.3, A04.5-A07, A07.2-A07.4, A08-A09.9, R19.7	001-001.9, 003-006.9, 007.4-007.8, 008.01-008.02, 008.04, 008.2-009.9, 787.91
Lower respiratory infections	A48.1, A70, J09-J15.8, J16-J16.9, J20-J21.9, P23.0-P23.4, U04-U04.9	073.0-073.6, 466-469, 470.0, 480-482.89, 483.0-483.9, 484.1-484.2, 484.6-484.7, 487-489
Upper respiratory infections	J01-J01.91, J04.0, J05-J05.0, J05.11, J36-J36.0	461-461.9, 464.0, 464.01, 464.11-464.2, 464.21, 464.31-464.4, 464.8-464.9, 475-475.9, 476.9
Diphtheria	A36-A36.9	032-032.9
Whooping cough	A37-A37.91	033-033.9, 484.3-484.4
Tetanus	A33-A35.0	037-037.9, 771.3
Measles	B05-B05.9	055-055.9, 323.1, 484.0
Maternal disorders	N96, N98-N98.9, O00-O07.9, O09-O16.9, O20-O26.93, O28-O36.93, O40-O48.1, O60-O77.9, O80-O92.79, O96-O99.91	630-636.92, 638-638.92, 640-679.14
Neonatal disorders	P00-P04.2, P04.5-P05.9, P07-P15.9, P19-P22.9, P24-P29.9, P36-P36.9, P38-P39.9, P50-P61.9, P70, P70.3-P72.9, P74-P78.9, P80-P81.9, P83-P84, P90-P94.9, P96, P96.3-P96.4, P96.8-P96.89	760-760.64, 760.8-768, 768.2-770, 770.1-771, 771.4-775, 775.4-779.34, 779.6-779.89
**Non-communicable diseases**		
Neoplasms		
Colon and rectum cancer	C18-C21.9, D01.0-D01.3, D12-D12.9, D37.3-D37.5	153-154.9, 209.1-209.17, 209.5-209.57, 211.3-211.4, 230.3-230.6
Non-melanoma skin cancer (squamous-cell carcinoma)	C44-C44.99, D04-D04.9, D49.2	173-173.99, 222.4, 232-232.9, 238.2
Breast cancer	C50-C50.929, D05-D05.92, D24-D24.9, D48.6-D48.62, D49.3, N60-N60.99	174-175.9, 217-217.8, 233.0, 238.3, 239.3, 610-610.9
Cervical cancer	C53-C53.9, D06-D06.9, D26.0	180-180.9, 219.0, 233.1
Uterine cancer	C54-C54.9, D07.0-D07.2, N87-N87.9	182-182.8, 233.2
Testicular cancer	C62-C62.92, D29.2-D29.8, D40.1-D40.8	186-186.9, 222.0, 222.3, 236.4
Hodgkin lymphoma	C81-C81.99	201-201.98
Leukaemia	C91-C95.92	204-208.92
Cardiovascular diseases		
Rheumatic heart disease	I01-I01.9, I02.0, I05-I09.9	391-391.9, 392.0, 393-398.99
Ischaemic heart disease	I20-I25.9	410-414.9
Cerebrovascular disease	G45-G46.8, I60-I61.9, I62.0-I62.03, I63-I63.9, I65-I66.9, I67.0-I67.3, I67.5-I67.6, I68.1-I68.2, I69.0-I69.398	430-435.9, 437.0-437.2, 437.5-437.8
Hypertensive heart disease	I11-I11.9	402-402.91
Chronic respiratory diseases	D86-D86.2, D86.89-D86.9, G47.3-G47.39, J30-J35.9, J37-J47.9, J60-J63.8, J65-J68.9, J70-J70.1, J70.8-J70.9, J82, J84-J84.9, J91-J92.9	135-135.9, 136.6, 327.2-327.8, 470, 470.9-474.9, 476-476.1, 477-479, 490-504.9, 506-506.9, 508-509, 515, 516-517.8, 518.6, 518.9, 519.1-519.4, 780.57, 786.03
Digestive diseases		
Peptic ulcer disease	K25-K28.9, K31, K31.1-K31.6, K31.8, K31.82-K31.89	531-534.91
Appendicitis	K35-K37.9, K38.3-K38.9	540-542.9
Inguinal, femoral, and abdominal hernia	K40-K42.9, K44-K46.9	550-551.1, 551.3-552.1, 552.3-553.03, 553.6
Gallbladder and biliary diseases	K80-K83.9	574-576.9
Neurological disorders		
Epilepsy	G40-G41.9	345-345.91
Diabetes, urogenital, blood, and endocrine diseases		
Diabetes mellitus	E10-E10.11, E10.3-E11.1, E11.3-E12.1, E12.3-E13.11, E13.3-E14.1, E14.3-E14.9, P70.0-P70.2, R73-R73.9	250-250.39, 250.5-250.99, 357.2, 775.0-775.1, 790.2-790.22
Chronic kidney disease	D63.1, E10.2-E10.29, E11.2-E11.29, E12.2, E13.2-E13.29, E14.2, I12-I13.9, N02-N08.8, N15.0, N18-N18.9	250.4-250.49, 403-404.93, 581-583.9, 585-585.9, 589-589.9
Other non-communicable diseases		
Congenital heart anomalies	Q20-Q28.9	745-747.9
**Injuries**		
Unintentional injuries		
Adverse effects of medical treatment	Y38.9-Y84.9, Y88-Y88.3	E870-E876.9, E878-E879.9, E930-E949.9

Supplementary Table 3. **Percent of deaths due to amenable causes from garbage codes among OECD countries reporting ICD9 and ICD10 in 2010.** Note that Iceland and Greece did not have ICD9 or ICD10 in 2010. OECD = Organisation for Economic Co-operation and Development. ICD = International Classification of Diseases.

Country	Percent garbage
Portugal	39.8%
Poland	37.9%
Turkey	34.9%
Belgium	34.4%
Luxembourg	32.7%
France	32.7%
Israel	32.0%
South Korea	30.6%
Netherlands	29.9%
Czech Republic	29.2%
Denmark	28.9%
United States	28.4%
Japan	28.1%
Chile	27.4%
Germany	26.5%
Spain	25.9%
Norway	25.9%
Italy	25.4%
Switzerland	23.2%
Canada	21.8%
Sweden	21.3%
Mexico	21.2%
United Kingdom	21.0%
Slovakia	21.0%
Slovenia	20.5%
Australia	20.4%
Ireland	17.6%
Austria	17.6%
Latvia	12.2%
Hungary	10.8%
Estonia	9.8%
New Zealand	9.4%
Finland	7.9%

***Supplementary Table 4.* Global mean, standard deviation, and skewness of the log of risk-standardised age-standardised death rates by cause amenable to healthcare.** These values represent 195 countries and territories in 1990, 1995, 2000, 2005, 2010, and 2015. Skewness reflects the magnitude of asymmetry of values from the mean.

Cause	Mean	Standard deviation	Skewness
**Communicable, maternal, neonatal, and nutritional diseases**			
Tuberculosis	-9.81	1.87	-0.02
Diarrhoea, lower respiratory, and other common infectious diseases			
Diarrhoeal diseases	-10.70	2.02	0.13
Lower respiratory infections	-8.14	0.94	-0.28
Upper respiratory infections	-13.44	0.38	1.50
Diphtheria	-13.53	0.61	3.32
Whooping cough	-12.62	1.36	1.10
Tetanus	-12.55	1.54	1.13
Measles	-12.24	1.84	0.92
Maternal disorders	-10.89	1.79	0.27
Neonatal disorders	-8.68	0.99	-0.52
**Non-communicable diseases**			
Neoplasms			
Colon and rectum cancer	-9.58	0.43	-0.09
Non-melanoma skin cancer (squamous-cell carcinoma)	-12.30	0.48	0.73
Breast cancer	-9.60	0.43	-0.13
Cervical cancer	-10.12	0.89	-0.02
Uterine cancer	-13.32	0.30	1.15
Testicular cancer	-12.96	0.38	0.58
Hodgkin lymphoma	-12.30	0.54	-0.21
Leukaemia	-11.18	0.38	0.00
Cardiovascular diseases			
Rheumatic heart disease	-10.54	0.90	-0.02
Ischaemic heart disease	-7.16	0.51	-0.05
Cerebrovascular disease	-7.54	0.69	-0.43
Hypertensive heart disease	-9.72	1.22	-0.23
Chronic respiratory diseases	-12.44	0.84	0.48
Digestive diseases			
Peptic ulcer disease	-10.47	0.93	-0.08
Appendicitis	-12.03	0.87	0.13
Inguinal, femoral, and abdominal hernia	-12.25	0.71	0.32
Gallbladder and biliary diseases	-11.44	0.69	0.17
Neurological disorders			
Epilepsy	-10.87	0.75	0.55
Diabetes, urogenital, blood, and endocrine diseases			
Diabetes mellitus	-10.99	0.83	0.02
Chronic kidney disease	-9.40	0.86	-0.41
Other non-communicable diseases			
Congenital heart anomalies	-10.25	0.58	-0.15
**Injuries**			
Unintentional injuries			
Adverse effects of medical treatment	-11.08	0.79	-0.14

Supplementary Table 5 . **Factor loadings and weights from principal component analysis and exploratory factor analysis.**

	Iteration # 1						Iteration # 2						Cause weight
	Factor 1	Factor 2	Factor 3	Factor 4	Factor 5	Composite factor	Factor 1	Factor 2	Factor 3	Factor 4	Factor 5	Composite factor	
**Principal component analysis**													
Tuberculosis	-0.2802	0.0708	0.0986	0.1157	-0.0501	-0.1807	-0.2818	0.0711	-0.1537	-0.0320	-0.0787	-0.2090	0.0554
Diarrhoeal diseases	-0.2944	0.0855	0.0269	-0.0921	-0.1309	-0.2087	-0.2957	0.0852	0.0536	0.0006	-0.1528	-0.2061	0.0546
Lower respiratory infections	-0.1745	0.0549	-0.1259	-0.0264	-0.0945	-0.1315	-0.1763	0.0574	0.1138	-0.1021	-0.0201	-0.1195	0.0317
Upper respiratory infections	-0.1453	0.0737	-0.0869	0.1097	0.0965	-0.0899	-0.1472	0.0769	-0.0258	-0.0569	0.1673	-0.0947	0.0251
Diphtheria	-0.0568	0.0125	-0.0031	0.0526	0.1209	-0.0309	-0.0576	0.0137	-0.0480	0.0403	0.1162	-0.0362	0.0096
Whooping cough	-0.1456	0.0216	0.0161	0.1370	0.0689	-0.0896	-0.1469	0.0231	-0.1147	-0.0359	0.1134	-0.1077	0.0285
Tetanus	-0.2706	0.3380	0.0146	0.0789	0.1969	-0.1376	-0.2726	0.3404	-0.0941	0.0943	0.1314	-0.1501	0.0398
Measles	-0.2039	0.2051	0.0913	0.2184	0.2137	-0.0930	-0.2057	0.2070	-0.2466	0.0566	0.1867	-0.1274	0.0337
Maternal disorders	-0.2724	0.1021	-0.0553	-0.0357	0.0014	-0.1873	-0.2745	0.1042	0.0582	0.0038	0.0047	-0.1818	0.0482
Neonatal disorders	-0.2645	-0.0950	0.1676	0.0082	-0.0461	-0.1912	-0.2653	-0.0970	-0.1211	0.0555	-0.0799	-0.2120	0.0562
Colon and rectum cancer	0.1237	-0.0426	-0.4956	0.2632	0.2260	**0.0756**	--	--	--	--	--	--	--
Non-melanoma skin cancer (squamous-cell carcinoma)	0.0002	-0.2868	-0.2286	-0.1226	-0.2112	-0.0659	-0.0005	-0.2851	0.2797	-0.1979	-0.0467	-0.0314	0.0083
Breast cancer	0.0220	-0.1941	-0.3995	-0.1437	0.2320	-0.0316	0.0196	-0.1878	0.3723	-0.0170	0.4142	**0.0282**	--
Cervical cancer	-0.1698	0.0163	-0.3523	-0.1419	-0.1811	-0.1576	-0.1724	0.0215	0.3662	-0.2077	-0.0398	-0.1122	0.0297
Uterine cancer	-0.0488	-0.1228	-0.1089	0.4595	0.0038	-0.0314	-0.0511	-0.1179	-0.2404	-0.3497	0.2441	-0.0743	0.0197
Testicular cancer	-0.0243	-0.2725	0.0260	-0.1780	0.2698	-0.0471	-0.0247	-0.2728	0.0776	0.2939	0.0766	-0.0300	0.0079
Hodgkin lymphoma	-0.0161	-0.4352	0.0994	-0.0055	0.2677	-0.0471	-0.0164	-0.4362	-0.0862	0.2017	0.1857	-0.0547	0.0145
Leukaemia	0.0044	-0.3710	0.0004	0.3005	-0.2952	-0.0388	0.0038	-0.3703	-0.1778	-0.3649	-0.1433	-0.0787	0.0208
Rheumatic heart disease	-0.1835	-0.1360	0.0528	0.2443	0.2728	-0.1188	-0.1857	-0.1336	-0.2364	0.0419	0.2788	-0.1525	0.0404
Ischaemic heart disease	-0.0795	-0.3396	0.1390	-0.0543	0.3311	-0.0780	-0.0799	-0.3411	-0.0870	0.2876	0.2556	-0.0817	0.0216
Cerebrovascular disease	-0.1617	-0.0711	-0.1244	0.2710	-0.0742	-0.1204	-0.1641	-0.0670	-0.0915	-0.2870	0.1383	-0.1418	0.0376
Hypertensive heart disease	-0.2233	0.0014	-0.1160	0.0348	0.0391	-0.1630	-0.2257	0.0047	0.0514	-0.0565	0.1016	-0.1586	0.0420
Chronic respiratory diseases	-0.1642	-0.0949	0.2044	0.0039	0.0111	-0.1150	-0.1646	-0.0972	-0.1583	0.1375	-0.1449	-0.1397	0.0370
Peptic ulcer disease	-0.2329	-0.0278	-0.0904	0.0482	0.1345	-0.1668	-0.2355	-0.0243	0.0125	0.0129	0.1253	-0.1673	0.0443
Appendicitis	-0.2417	-0.0343	0.0030	-0.1882	0.0011	-0.1869	-0.2431	-0.0342	0.1207	0.1400	-0.1293	-0.1712	0.0454
Inguinal, femoral, and abdominal hernia	-0.2383	-0.0471	0.0349	-0.3744	0.1851	-0.1861	-0.2394	-0.0479	0.2077	0.3897	-0.1094	-0.1517	0.0402
Gallbladder and biliary diseases	-0.1717	-0.0469	-0.1972	-0.1353	-0.1244	-0.1539	-0.1736	-0.0440	0.2425	-0.0844	-0.1015	-0.1250	0.0331
Epilepsy	-0.1706	-0.0031	-0.2658	-0.0026	-0.1896	-0.1477	-0.1727	0.0008	0.2177	-0.2803	0.0710	-0.1232	0.0326
Diabetes mellitus	-0.1708	-0.1553	-0.0479	-0.1627	-0.0999	-0.1574	-0.1720	-0.1550	0.1530	0.0277	-0.1498	-0.1395	0.0370
Chronic kidney disease	-0.1342	-0.2493	0.2753	-0.0039	-0.3139	-0.1228	-0.1339	-0.2534	-0.1790	0.0115	-0.5206	-0.1596	0.0423
Congenital heart anomalies	-0.1121	-0.1346	0.1119	0.2601	-0.1724	-0.0826	-0.1129	-0.1349	-0.2461	-0.1996	-0.0937	-0.1272	0.0337
Adverse effects of medical treatment	-0.1458	-0.0765	-0.1409	-0.0521	-0.0953	-0.1296	-0.1472	-0.0746	0.1528	-0.1228	0.0769	-0.1100	0.0291
Variance explained	*0.5791*	*0.0998*	*0.0522*	*0.0448*	*0.0357*	--	*0.5887*	*0.1028*	*0.0487*	*0.0408*	*0.0319*	--	

	Iteration # 1						Iteration # 2						Cause weight
	Factor 1	Factor 2	Factor 3	Factor 4	Factor 5	Composite factor	Factor 1	Factor 2	Factor 3	Factor 4	Factor 5	Composite factor	
**Exploratory factor analysis**													
Tuberculosis	0.8563	0.0121	0.3349	0.1398	--	0.4563	0.8735	0.0399	-0.2761	0.1312	--	0.4532	0.0415
Diarrhoeal diseases	0.8777	0.0179	0.2752	-0.0328	--	0.4257	0.8905	0.0210	-0.0829	-0.0435	--	0.4348	0.0398
Lower respiratory infections	0.8276	-0.1434	0.0991	0.0350	--	0.3647	0.8182	-0.1530	0.0868	0.0463	--	0.3871	0.0354
Upper respiratory infections	0.8192	-0.0655	0.0084	0.1841	--	0.4054	0.8207	-0.0733	-0.0871	0.2320	--	0.4330	0.0396
Diphtheria	0.3744	0.1111	0.0202	0.1094	--	0.2264	0.3773	0.1139	-0.0539	0.1410	--	0.2397	0.0219
Whooping cough	0.6982	0.0415	0.1703	0.2213	--	0.3953	0.7045	0.0418	-0.1280	0.2367	--	0.4003	0.0366
Tetanus	0.8066	-0.1224	0.1293	-0.0191	--	0.3506	0.8304	-0.1181	-0.3034	0.0057	--	0.3676	0.0336
Measles	0.6692	-0.1205	0.2052	0.0953	--	0.3176	0.6850	-0.0991	-0.3111	0.1085	--	0.3197	0.0293
Maternal disorders	0.8947	-0.0559	0.1521	0.0074	--	0.4152	0.8999	-0.0646	-0.0534	0.0131	--	0.4327	0.0396
Neonatal disorders	0.7801	0.1638	0.3105	0.0612	--	0.4389	0.7938	0.1573	-0.1023	0.0142	--	0.4289	0.0392
Colon and rectum cancer	-0.3670	-0.0751	-0.7285	0.2845	--	**-0.1814**	--	--	--	--	--	--	--
Non-melanoma skin cancer (squamous-cell carcinoma)	0.0402	0.3881	-0.1685	0.1486	--	0.1330	0.0085	0.3559	0.6342	0.1548	--	0.1604	0.0147
Breast cancer	0.0081	0.3173	-0.7819	-0.0975	--	0.0017	-0.0432	0.2358	0.7798	-0.0153	--	0.0818	0.0075
Cervical cancer	0.8831	0.0030	-0.1175	0.0159	--	0.4059	0.8666	-0.0166	0.2938	0.0505	--	0.4576	0.0419
Uterine cancer	0.1733	0.0641	-0.0902	0.7896	--	0.2607	0.1490	0.0648	0.0428	0.8065	--	0.2545	0.0233
Testicular cancer	0.0712	0.6487	-0.0271	0.0150	--	0.1925	0.0701	0.6442	0.1317	0.0077	--	0.1963	0.0180
Hodgkin lymphoma	-0.0850	0.8319	-0.0121	0.2135	--	0.2087	-0.0877	0.8167	0.2001	0.1866	--	0.1986	0.0182
Leukaemia	-0.1085	0.3905	0.0783	0.6574	--	0.1932	-0.1288	0.3888	0.1817	0.5871	--	0.1570	0.0144
Rheumatic heart disease	0.6585	0.2359	0.1174	0.3520	--	0.4487	0.6593	0.2250	-0.1467	0.3534	--	0.4430	0.0405
Ischaemic heart disease	0.1921	0.4775	0.0012	0.0397	--	0.2144	0.1845	0.4313	0.1559	0.0232	--	0.2081	0.0190
Cerebrovascular disease	0.7208	-0.0110	0.0354	0.4285	--	0.4279	0.7066	-0.0196	-0.0039	0.4308	--	0.4341	0.0397
Hypertensive heart disease	0.8740	-0.0091	0.0518	0.1264	--	0.4353	0.8713	-0.0358	0.0239	0.1396	--	0.4557	0.0417
Chronic respiratory diseases	0.5900	0.2046	0.3937	0.1264	--	0.3809	0.6003	0.2061	-0.1285	0.0855	--	0.3565	0.0326
Peptic ulcer disease	0.8789	0.1926	0.0782	0.1772	--	0.4998	0.8824	0.1813	-0.0917	0.1940	--	0.5155	0.0472
Appendicitis	0.8666	0.1678	0.2119	-0.0408	--	0.4506	0.8715	0.1533	-0.0379	-0.0530	--	0.4574	0.0418
Inguinal, femoral, and abdominal hernia	0.8268	0.3711	0.1714	-0.1973	--	0.4448	0.8425	0.3587	-0.0819	-0.1983	--	0.4591	0.0420
Gallbladder and biliary diseases	0.8489	0.1260	0.0369	0.0626	--	0.4417	0.8374	0.1054	0.1640	0.0663	--	0.4664	0.0427
Epilepsy	0.7866	-0.0663	-0.0300	0.0789	--	0.3643	0.7739	-0.0775	0.2183	0.1063	--	0.4034	0.0369
Diabetes mellitus	0.7617	0.2025	0.1502	0.0512	--	0.4258	0.7526	0.1713	0.1939	0.0315	--	0.4345	0.0398
Chronic kidney disease	0.4013	0.2230	0.5310	0.2639	--	0.3377	0.4001	0.2333	-0.0872	0.1689	--	0.2825	0.0258
Congenital heart anomalies	0.4280	0.1187	0.2491	0.4332	--	0.3404	0.4303	0.1215	-0.1346	0.3424	--	0.3032	0.0277
Adverse effects of medical treatment	0.6053	-0.0006	-0.0175	0.0022	--	0.2804	0.5888	-0.0326	0.3039	0.0063	--	0.3071	0.0281
Variance explained	*0.4345*	*0.2279*	*0.0696*	*0.2027*	--	--	*0.4463*	*0.2101*	*0.0586*	*0.1800*	--	--	

Supplementary Table 6 **. Country and territory-level frontier values of the HAQ Index by five-year interval from 1990 to 2015.** Geographies are organised by GBD super-region and region. HAQ Index = Healthcare Access and Quality Index. GBD = Global Burden of Disease.

Location	HAQ Index frontier value					
	1990	1995	2000	2005	2010	2015
**Global**	**61.9**	**64.5**	**67.2**	**70.0**	**72.4**	**73.8**
**Southeast Asia, East Asia, and Oceania**	**57.6**	**63.0**	**67.2**	**71.0**	**73.6**	**75.0**
East Asia	57.1	63.1	67.8	71.7	74.0	75.4
China	56.2	62.7	67.3	71.5	73.9	75.4
North Korea	70.6	71.1	68.5	67.1	68.1	69.1
Taiwan	76.8	79.8	82.6	85.7	87.5	88.6
Southeast Asia	59.0	62.9	66.2	69.5	72.4	74.0
Cambodia	45.4	47.3	49.0	51.6	57.7	62.3
Indonesia	60.6	65.0	68.7	70.8	72.9	74.4
Laos	46.3	48.3	50.2	54.1	59.7	63.9
Malaysia	69.3	72.4	74.6	76.7	79.3	81.5
Maldives	50.1	57.2	63.4	68.1	71.5	73.2
Mauritius	68.6	72.1	73.9	75.0	76.7	79.0
Myanmar	45.8	46.8	48.9	53.1	60.8	64.9
Philippines	63.8	65.9	68.2	70.5	72.6	74.1
Sri Lanka	66.3	69.4	72.1	73.5	74.8	76.8
Seychelles	72.2	74.5	75.9	77.3	78.6	80.9
Thailand	66.5	71.0	72.9	74.1	75.2	76.8
Timor-Leste	46.6	47.6	45.3	49.0	54.4	59.1
Vietnam	52.2	58.6	63.7	67.6	71.4	73.5
Oceania	51.3	53.7	56.0	57.5	60.0	62.9
American Samoa	74.0	75.2	77.1	77.3	77.3	77.4
Federated States of Micronesia	62.5	64.8	67.5	70.4	72.2	73.3
Fiji	69.2	71.6	73.1	74.1	75.1	76.1
Guam	81.7	84.0	85.7	87.4	88.8	89.8
Kiribati	53.3	54.2	57.0	59.5	60.6	61.7
Marshall Islands	56.8	61.8	63.0	65.1	68.2	71.4
Northern Mariana Islands	82.2	84.0	86.5	87.7	87.4	87.0
Papua New Guinea	48.7	49.7	50.7	51.5	53.9	58.8
Samoa	63.4	65.8	68.2	71.0	72.7	73.8
Solomon Islands	48.5	50.2	52.5	52.8	55.8	60.2
Tonga	63.8	65.9	68.0	70.5	72.3	73.2
Vanuatu	52.8	55.7	59.2	61.8	64.3	66.4
**Central Europe, Eastern Europe, and Central Asia**	**76.7**	**77.8**	**78.5**	**80.6**	**83.4**	**85.3**
Central Asia	72.3	72.8	72.9	74.2	75.9	78.2
Armenia	71.7	71.3	72.3	74.7	77.6	80.6
Azerbaijan	74.0	74.3	73.9	75.2	80.1	83.3
Georgia	76.1	74.9	74.0	75.6	78.3	81.1
Kazakhstan	75.5	76.7	77.5	79.6	82.2	84.7

Location	HAQ Index frontier value					
	1990	1995	2000	2005	2010	2015
Kyrgyzstan	71.5	71.7	70.9	71.7	72.6	73.6
Mongolia	64.4	68.7	71.6	73.5	74.8	76.8
Tajikistan	63.8	63.5	60.5	63.1	66.7	70.0
Turkmenistan	71.7	73.4	73.9	75.4	79.0	82.6
Uzbekistan	67.1	69.3	71.3	73.0	74.6	76.5
Central Europe	76.2	77.4	79.9	82.3	84.6	86.4
Albania	69.7	70.0	72.9	75.0	77.0	79.1
Bosnia and Herzegovina	62.5	63.6	73.2	75.4	77.4	79.3
Bulgaria	76.6	77.7	78.6	80.4	82.9	84.8
Croatia	75.0	75.2	76.5	79.0	81.3	82.9
Czech Republic	83.0	85.0	86.9	88.0	89.3	90.2
Hungary	78.4	79.8	82.0	84.3	86.1	87.6
Macedonia	74.1	74.6	75.3	76.9	79.3	81.1
Montenegro	75.8	75.8	76.8	79.0	82.0	84.0
Poland	76.8	79.0	82.6	84.8	86.7	88.8
Romania	76.1	76.7	77.5	79.4	82.2	84.0
Serbia	74.4	74.6	75.1	77.4	80.1	81.9
Slovakia	77.3	79.3	82.0	84.3	86.7	88.5
Slovenia	80.4	81.9	84.1	86.0	87.4	88.0
Eastern Europe	79.4	80.8	80.7	82.9	85.5	87.2
Belarus	75.8	76.8	77.9	81.0	84.8	87.4
Estonia	77.3	79.0	81.2	84.0	86.4	88.3
Latvia	79.0	79.7	81.0	83.8	86.5	88.4
Lithuania	77.6	78.3	79.7	82.9	85.3	87.0
Moldova	72.8	72.8	71.8	73.4	74.9	76.7
Russia	81.1	82.6	82.5	84.3	86.6	88.1
Ukraine	76.7	77.7	77.4	79.7	83.1	85.0
**High-income**	**83.3**	**85.0**	**86.7**	**87.9**	**89.0**	**90.0**
High-income Asia Pacific	82.6	85.1	87.0	88.3	89.3	90.0
Brunei	83.0	85.8	89.0	90.2	90.8	91.3
Japan	84.5	86.7	88.1	89.1	89.8	90.4
South Korea	77.3	80.6	84.0	86.4	87.9	89.0
Singapore	77.3	80.8	83.7	86.7	88.6	89.6
Australasia	84.5	86.1	87.9	89.1	90.0	90.8
Australia	84.7	86.3	88.1	89.3	90.2	91.0
New Zealand	83.4	85.0	86.7	87.9	88.8	89.9
Western Europe	80.9	83.0	84.9	86.4	87.7	88.8
Andorra	85.8	87.2	88.4	89.6	90.4	91.2
Austria	83.2	84.8	86.5	88.0	89.2	90.0
Belgium	82.6	84.3	86.0	87.4	88.5	89.6
Cyprus	78.2	81.0	83.4	86.0	88.5	89.5

Location	HAQ Index frontier value					
	1990	1995	2000	2005	2010	2015
Denmark	85.6	86.9	88.6	89.6	90.4	90.9
Finland	83.0	84.3	86.3	87.9	89.3	90.2
France	79.0	80.6	82.2	83.8	85.3	86.7
Germany	83.5	85.8	87.4	88.8	89.9	90.6
Greece	77.0	79.2	81.5	83.5	85.3	85.9
Iceland	83.1	84.6	86.7	88.1	89.5	90.3
Ireland	79.0	81.9	84.8	87.1	88.6	90.0
Israel	80.3	82.2	84.0	85.3	86.2	87.1
Italy	81.8	83.4	84.8	86.0	87.1	88.1
Luxembourg	83.6	85.8	87.9	89.2	90.3	90.9
Malta	74.9	75.8	78.4	81.1	83.0	84.5
Netherlands	83.2	85.0	86.7	88.1	89.5	90.3
Norway	85.9	87.6	89.6	90.6	91.3	91.6
Portugal	71.4	73.5	74.9	76.4	78.4	80.5
Spain	74.7	76.5	78.9	81.4	83.8	85.7
Sweden	82.9	85.0	87.3	88.3	89.3	90.2
Switzerland	86.4	88.1	89.4	90.3	91.0	91.4
United Kingdom	83.4	85.4	87.5	88.9	89.6	90.3
Southern Latin America	73.4	74.7	76.4	77.7	80.1	82.6
Argentina	73.3	74.6	76.1	77.2	79.6	81.9
Chile	73.8	75.3	77.6	79.8	82.3	84.5
Uruguay	72.5	73.7	74.8	75.3	77.0	79.7
High-income North America	88.5	89.2	89.9	90.5	91.1	91.5
Canada	88.1	89.2	90.3	90.9	91.3	91.6
Greenland	72.5	73.3	74.8	75.1	76.8	81.0
United States	88.5	89.2	89.9	90.5	91.1	91.5
**Latin America and Caribbean**	**65.4**	**68.5**	**71.2**	**72.9**	**74.2**	**75.3**
Caribbean	68.5	70.0	71.7	73.0	74.0	74.9
Antigua and Barbuda	79.2	81.1	82.9	84.4	86.2	87.0
The Bahamas	77.9	79.6	82.6	85.0	85.8	86.6
Barbados	75.6	76.8	78.5	80.3	81.8	82.8
Belize	61.4	64.5	68.9	72.4	73.8	74.8
Bermuda	86.9	88.2	89.2	90.2	90.9	91.1
Cuba	74.8	74.9	75.3	76.8	79.3	81.5
Dominica	72.0	73.8	75.3	76.7	78.6	80.4
Dominican Republic	64.1	66.8	70.5	72.7	74.2	75.6
Grenada	67.3	72.7	74.7	76.7	78.8	80.5
Guyana	62.7	65.3	69.0	71.1	72.8	74.4
Haiti	48.2	48.9	49.7	50.7	52.0	54.6
Jamaica	71.2	73.3	74.3	75.2	76.5	77.7
Puerto Rico	81.7	84.0	86.0	87.8	88.9	89.6

Location	HAQ Index frontier value					
	1990	1995	2000	2005	2010	2015
Saint Lucia	69.9	73.0	74.8	76.3	77.7	79.4
Saint Vincent and the Grenadines	71.0	73.4	74.8	76.4	78.4	80.0
Suriname	67.5	69.7	72.0	73.7	75.0	76.7
Trinidad and Tobago	75.5	76.8	79.3	82.4	85.0	86.4
US Virgin Islands	81.2	84.8	87.2	88.3	89.2	89.9
Andean Latin America	64.9	67.6	70.6	72.6	74.2	75.6
Bolivia	56.6	60.3	63.5	66.5	70.0	72.6
Ecuador	66.2	69.3	71.7	73.2	74.5	75.7
Peru	66.7	69.3	72.0	73.6	75.0	76.8
Central Latin America	66.6	69.9	72.3	73.7	74.9	76.1
Colombia	67.1	70.4	72.5	73.7	74.9	76.5
Costa Rica	68.6	71.4	73.5	74.7	76.1	78.0
El Salvador	57.2	61.5	64.8	68.4	71.4	73.0
Guatemala	50.7	53.1	57.5	61.4	64.2	66.9
Honduras	51.5	54.8	59.1	62.7	66.2	69.5
Mexico	68.9	72.0	73.7	74.8	76.1	77.6
Nicaragua	53.1	56.1	60.4	63.4	65.9	68.9
Panama	71.6	73.0	74.2	75.2	77.0	79.9
Venezuela	71.4	73.2	74.1	75.0	76.7	78.5
Tropical Latin America	63.7	66.6	69.5	72.0	73.6	74.7
Brazil	63.8	66.6	69.6	72.1	73.7	74.7
Paraguay	61.7	64.2	67.3	70.1	72.4	74.0
**North Africa and Middle East**	**55.7**	**60.8**	**64.2**	**67.5**	**70.5**	**72.3**
North Africa and Middle East	55.7	60.8	64.2	67.5	70.5	72.3
Afghanistan	38.0	38.8	37.8	41.4	44.7	47.6
Algeria	58.3	63.7	67.5	69.7	70.4	71.4
Bahrain	71.8	74.3	75.7	77.7	81.2	82.3
Egypt	58.0	63.1	67.0	70.4	72.1	73.0
Iran	60.0	67.2	72.7	74.8	76.4	77.5
Iraq	53.2	56.4	61.9	64.8	66.5	70.1
Jordan	63.1	69.2	71.7	73.6	74.9	76.3
Kuwait	76.0	77.9	81.5	83.4	86.7	88.5
Lebanon	69.5	72.1	74.0	75.8	77.8	80.5
Libya	61.5	66.4	70.1	72.4	73.9	74.0
Morocco	49.4	51.1	54.7	58.0	60.7	63.0
Palestine	55.8	61.1	66.8	67.8	67.5	69.4
Oman	54.3	64.9	72.5	74.6	75.8	78.6
Qatar	72.9	74.6	75.7	78.9	82.3	84.5
Saudi Arabia	65.4	69.8	74.0	76.0	78.6	81.0
Sudan	46.6	47.6	48.8	50.2	52.9	56.4
Syria	52.1	57.9	62.1	65.4	70.5	70.4

Location	HAQ Index frontier value					
	1990	1995	2000	2005	2010	2015
Tunisia	59.1	64.4	69.0	72.0	73.5	74.3
Turkey	65.2	68.5	71.4	73.3	74.6	75.9
United Arab Emirates	73.7	78.4	82.7	85.8	88.9	89.2
Yemen	37.0	42.6	46.2	48.8	51.3	54.1
**South Asia**	**48.5**	**49.9**	**52.9**	**58.0**	**62.7**	**66.3**
South Asia	48.5	49.9	52.9	58.0	62.7	66.3
Bangladesh	47.0	48.6	49.9	52.1	56.7	61.1
Bhutan	47.3	49.0	51.9	58.0	62.6	65.9
India	48.8	50.4	54.1	59.5	63.9	68.4
Nepal	44.4	46.4	48.2	49.5	51.5	55.7
Pakistan	47.2	48.9	50.6	53.5	57.5	60.7
**Sub-Saharan Africa**	**46.6**	**47.3**	**47.9**	**48.7**	**49.9**	**52.3**
Central Sub-Saharan Africa	45.7	45.4	44.3	43.1	45.7	48.1
Angola	46.1	47.0	47.8	48.9	51.1	55.3
Central African Republic	44.0	44.8	45.5	46.2	47.3	47.3
Congo	53.6	56.4	58.8	60.8	63.2	65.5
Democratic Republic of the Congo	44.7	43.8	40.6	37.0	40.9	45.2
Equatorial Guinea	46.4	47.5	55.8	65.2	70.0	72.5
Gabon	61.1	63.9	67.2	70.3	72.4	74.0
Eastern Sub-Saharan Africa	43.1	44.0	45.2	46.6	48.4	49.9
Burundi	39.9	41.0	40.7	42.2	43.8	45.3
Comoros	46.0	47.1	48.1	49.0	49.7	50.5
Djibouti	48.9	49.7	50.9	52.8	56.7	60.2
Eritrea	41.5	44.2	47.1	48.3	48.4	48.9
Ethiopia	36.8	36.9	38.2	41.1	45.2	48.1
Kenya	49.5	51.6	53.1	54.6	57.6	61.1
Madagascar	46.4	47.0	47.9	48.9	49.8	50.8
Malawi	42.4	42.5	44.3	44.9	46.8	48.4
Mozambique	31.6	31.6	36.8	41.8	45.0	47.1
Rwanda	43.3	43.7	45.0	46.6	48.8	51.0
Somalia	35.5	34.6	34.4	35.0	36.9	38.6
South Sudan	38.0	39.0	41.5	43.3	45.1	46.4
Tanzania	47.0	47.8	48.5	49.4	51.0	54.6
Uganda	43.2	44.5	46.2	47.7	49.4	51.3
Zambia	49.0	49.4	49.9	51.1	55.5	60.7
Southern Sub-Saharan Africa	65.3	68.2	70.8	72.6	73.7	74.8
Botswana	55.4	62.3	66.2	70.3	72.6	73.9
Lesotho	49.1	50.6	53.4	57.9	61.8	65.1
Namibia	58.0	61.7	64.8	68.0	70.8	72.9
South Africa	69.7	71.7	73.0	74.2	75.5	77.1
Swaziland	55.0	60.8	65.0	67.1	71.0	73.3

Location	HAQ Index frontier value					
	1990	1995	2000	2005	2010	2015
Zimbabwe	56.0	60.7	63.5	64.0	63.6	66.6
Western Sub-Saharan Africa	46.2	47.1	48.1	49.2	50.7	53.3
Benin	42.8	44.4	45.8	47.4	48.6	49.7
Burkina Faso	33.1	35.0	37.5	40.2	43.1	45.2
Cameroon	48.9	49.7	50.8	53.3	56.8	60.4
Cape Verde	48.3	49.9	54.6	61.0	64.8	67.6
Chad	38.1	39.2	40.3	43.1	45.7	47.5
Cote d'Ivoire	46.3	47.4	48.6	49.2	49.9	51.5
The Gambia	45.2	45.7	46.7	47.6	48.3	49.0
Ghana	49.8	51.7	54.6	57.9	61.1	64.2
Guinea	40.4	42.4	43.7	44.9	46.0	47.1
Guinea-Bissau	40.8	42.9	44.6	45.7	46.8	47.8
Liberia	43.9	40.1	41.0	43.9	45.4	47.3
Mali	35.1	37.3	39.3	41.4	43.3	44.8
Mauritania	46.6	47.5	48.5	49.3	50.7	53.4
Niger	32.6	33.5	34.3	35.3	36.4	38.2
Nigeria	48.2	48.9	49.7	52.1	57.1	61.4
Sao Tome and Principe	48.1	49.0	49.8	51.5	55.0	58.8
Senegal	43.5	45.1	46.4	47.7	48.5	49.4
Sierra Leone	41.3	42.6	43.0	45.2	47.0	48.9
Togo	45.5	46.7	47.8	48.5	49.2	50.3
